# Abstract Profiles of Structural Stability Point to Universal Tendencies, Family-Specific Factors, and Ancient Connections between Languages

**DOI:** 10.1371/journal.pone.0045198

**Published:** 2012-09-20

**Authors:** Dan Dediu, Stephen C. Levinson

**Affiliations:** 1 Language and Genetics, Max Planck Institute for Psycholinguistics, Nijmegen, The Netherlands; 2 Language and Cognition, Max Planck Institute for Psycholinguistics, Nijmegen, The Netherlands; 3 Donders Institute for Brain, Cognition and Behaviour, Radboud University Nijmegen, Nijmegen, The Netherlands; 4 Radboud University Nijmegen, Nijmegen, The Netherlands; Durham University, United Kingdom

## Abstract

Language is the best example of a cultural evolutionary system, able to retain a phylogenetic signal over many thousands of years. The temporal stability (conservatism) of basic vocabulary is relatively well understood, but the stability of the structural properties of language (phonology, morphology, syntax) is still unclear. Here we report an extensive Bayesian phylogenetic investigation of the structural stability of numerous features across many language families and we introduce a novel method for analyzing the relationships between the “stability profiles” of language families. We found that there is a strong universal component across language families, suggesting the existence of universal linguistic, cognitive and genetic constraints. Against this background, however, each language family has a distinct stability profile, and these profiles cluster by geographic area and likely deep genealogical relationships. These stability profiles seem to show, for example, the ancient historical relationships between the Siberian and American language families, presumed to be separated by at least 12,000 years, and possible connections between the Eurasian families. We also found preliminary support for the punctuated evolution of structural features of language across families, types of features and geographic areas. Thus, such higher-level properties of language seen as an evolutionary system might allow the investigation of ancient connections between languages and shed light on the peopling of the world.

## Introduction

Historical linguistics [1] investigates the genealogical relationships between languages using a time-honored and complex methodology [2]. Recently, striking parallels between language and other evolutionary systems – biological and cultural – have been identified [3], [4] prompting an increasingly successful use of modern phylogenetic methods inspired by evolutionary biology [5]–[9]. A major area of current interest concerns the stability over time of various components of language and what they can reveal about human history and universal constraints with origins in human cognition and learning [6], [7], [10]. The rates of replacement in the basic vocabulary (or Swadesh list [11]) – the 200 odd wordforms expressing the most stable meanings in language – are relatively well understood [12], with the frequency of use being suggested as an important explanatory factor in recent work by Pagel and colleagues [4], [5]. These rates seem to be correlated across language families, so that lexical meanings stable in, for example, Indo-European languages also tend to be stable in Bantu or Austronesian languages [5], [8], [13], as well as across extremely broad geographical regions [14].

The maximal timedepth of historical reconstruction using vocabulary methods is generally conceded to lie at about 10,000 years before present [15], leaving scant hope of connecting the 250+ language families of the world [16] or of revealing relationships that stretch back into the Pleistocene. However, it is possible that *structural features* (such as aspects of the phoneme inventories, morphology and syntax) might well be able to preserve information about more ancient relationships. One added level of complexity in studying such structural features is that they represent abstractions over patterns across many languages and that their values necessarily include a degree of subjectivity. For example, even apparently simple and uncontroversial concepts such as “noun” and “verb” present difficulties when viewed across widely different languages [17] making cross-linguistic comparisons extremely difficult [18]. In this context, the questions then are (i.) whether it is possible to isolate the most stable structural features, akin to the conservative basic vocabulary, and (ii.) what this might reveal about the evolution of current linguistic diversity.

Unfortunately, the stability of the structural features of language is currently less well understood and has proved more controversial due to divergent empirical findings and theoretical positions. There are several possible approaches to defining and quantifying the stability of structural features (see, for some recent examples [19]–[22]) varying in the accent placed on the *vertical* (genealogical) and *horizontal* (contact) processes in language. There are suggestions, such as Nichols’ [16] work in linguistic typology and the more recent phylogenetic approaches of Dunn and colleagues [6], [10], that structural features are stable enough to retain phylogenetic signals of relationships between languages over much deeper time depths than the most conserved vocabulary, and that they might even be better than genetic markers at conserving a vertical historical signal against population admixture [10]. On the other hand, a recent comparison conducted by Greenhill and colleagues [8] of structural features and the basic vocabulary suggests that structure and vocabulary have similar stabilities (a finding also supported by a different approach [22]), but structural features might be more prone to borrowing, making them less reliable sources of information about the genealogical relationships between languages (see also [23] for a similar suggestion). The study notably suggests that the stability of structural features varies across language families [8], leading the authors to claim that their findings “do not support the existence of a set of universally stable typological features” (p.6). This pessimistic conclusion about the prospects for using structural features for historical purposes may seem supported by the recent findings by Dunn and colleagues [7] that patterns of correlated evolution among types of word order are different among four major language families. This is in apparent contrast, however, with the report by Dediu [9] that there is agreement on the stability of structural features across a large sample of language families, suggesting that the stability of a particular structural feature tends to be independent of the language family concerned.

How are we to reconcile these divergent findings? Are structural features more stable or less stable than the basic vocabulary? Are some structural features inherently more stable than others (in a manner similar to the basic vocabulary) or is their stability fully determined by idiosyncratic properties and historical contingencies specific to each language family? And can we use structural features to peer into the deep past, beyond the 10,000 years horizon of the classic comparative method in linguistics?

It will take a much more sustained effort to use structural features in historical reconstruction before we will have definitive answers to these questions. But meanwhile we believe that by taking a more abstract approach we may be able to offer a reconciliation of these divergent opinions, while providing important groundwork for future progress in this area. We show here that the cultural evolution of structural features is simultaneously shaped by *universal tendencies*, *language family-specific factors* and *deep genealogical and areal processes* acting across language families. Thus, the dichotomy between universal tendencies and language family-specificity in what concerns structural stability is a *false* one, given that all three levels are present at the same time. This three-way partitioning of structural stability among language families is metaphorically similar to the structure of our species: we are, simultaneously, fundamentally the same as each other while being unique individuals who are more similar within kin groups than across them. Or, as Murray and Kluckhohn [24] put it “Every man is in certain respects (a) like all other men, (b) like some other men, (c) like no other man” (p. 53). The *universal component* – Murray and Kluckhohn’s (a) –, whereby some aspects of language tend to be stable across all families, might point to biological and cognitive biases affecting language acquisition, usage and processing [25], [26]. The *language family-specific factors* – Murray and Kluckhohn’s (c) – include idiosyncratic affordances for language change [7] and historical accidents. Finally, the differences between families are not entirely unconstrained – Murray and Kluckhohn’s (b) – and we show here that they might be *patterned by deep historical relationships* between languages.

Rather than directly using the patterns of values of structural features to infer the historical relationships between languages, we here propose investigating the patterns of stability of these features across language families. In this manner, we use the language families constructed independently and prior to the application of our method (and ideally using the historical linguistic comparative method) to infer the stability of structural features in those families – what we call here the language family’s *stability profile*. Essentially, the stability profile of a language family represents the relative stabilities (from the most stable to the most unstable) of a set of structural features in this family. The stability profile of a family is an abstract, mathematical concept which is in itself completely agnostic as to the existence or not of universal tendencies, language family-specific and intra-family processes. Only *sets* of stability profiles computed for several families can shed light on such questions through their mutual relationships.

We use these stability profiles estimated for several language families to infer deeper relationships between these families, on the assumption that while *individual* structural features might be relatively easily transferred across language (and even language family) borders or change in a short time, the stability profiles might be more resistant to such processes. This is due to the fact that a stability profile summarizes the historical changes of the *whole set of structural features* across a *whole set of related languages* during the *entire history of the family*. Borrowing one or more features would not dramatically change the stability profile of the language family or families involved, which require alterations to coherent systems of many inter-related features where components are not free to change at will (as Meillet put it “… que chaque langue forme un système où tout se tient …”; in our translation from French: “… that every language is a system where all parts interact …”) [27]). Certainly, there are cases of important restructuring where several features change together, and in intense contact situations this restructuring can be massive, but it probably rarely affects enough members of a language family in such a coherent manner that it will alter the family’s stability profile. We suggest that, as in genetics [28], some features might be hubs in the structural network of the language system while others are more peripheral, with the first type more resistant to change and borrowing and the second more prone to it, as proposed by the (extended) complexity hypothesis in evolutionary biology [29], [30]. Such an account may be consistent with the frequency explanation shown to play a role in vocabulary [4] in that hub structures may be more frequently used in linguistic exchanges and thus resistant to change.

To pursue these issues, we examined the stability of a large set of typological features across many language families, under a range of different assumptions to test the robustness of the findings. Here, we understand *stability* in a genealogical (vertical) context as the tendency of a structural feature to retain its ancestral value across subsequent language splits. Thus, a stable feature will tend to have the same value across all languages descended from the same proto-language. This is but one possible meaning of stability as applied to linguistic typology, but it is the currently best quantified and understood type of stability due to its parallels in evolutionary biology (see Section “Comparing structural stability across methods”). For a given language family, we estimated the stability of a set of features using a Bayesian phylogenetic approach which takes as given the language family tree and the observed feature values in the family’s languages. Of relevance here is that the Bayesian phylogenetic software produces posterior distributions of estimates of ancestral states (values that the features had at the tree-internal nodes) and the rates at which feature values have changed across the tree.

In order to control for various sources of potential biases, we used several different Bayesian phylogenetic software packages, different quantifications of stability, different outgroup choices, language classifications and data codings [9], resulting in 12 distinct datasets. Due to distinct assumptions and codings, the datasets have different degrees of resolution, but the results correlate to a very high degree; consequently, but solely for presentation purposes, we illustrate here with a single representative dataset (see Materials and Methods). We compare the resulting stability estimates across language families and show that, in addition to a background agreement in feature stability, the variance in stability between language families is geographically and historically patterned.

This approach, using higher-level properties of language viewed as a system evolving through time, promises to open up a window on processes that have shaped human prehistory on a deep time scale lying beyond the currently available methods.

## Results

Drawing on *The World Atlas of Language Structures* [31], [32], we estimated the stability of a large set of structural features (such as phoneme inventories, word order or types of negation; see **Materials S1** for the full list) across more than 50 language families in total using a Bayesian phylogenetic approach. More specifically, to assess the robustness of the findings, we used two different Bayesian phylogenetic software packages (MrBayes 3 [33] and BayesLang [9]), several outgroup choices, three different language classifications (WALS [31], the Ethnologue [34] and a collection of more orthodox historical linguistic classifications [35]) and two types of data codings (binary and polymorphic), resulting in 12 distinct datasets (**Materials and Methods**, Section **Primary data and stability estimation**). This procedure allowed us to control for the influence of various sources of potential biases, including the specific method for estimating rates of change on phylogenies, coding biases in the data, and the effect of the classifications of languages into genealogical units and of the degree of resolution of these classifications. Because the two codings result in different numbers of (polymorphic vs. binary) features and the two software packages used have different assumptions and minimum requirements, the composition of the 12 resulting datasets differs in details (see **Materials S1**), but the results reported below are similar.

### Structural Features of Language Evolve in Punctuational Bursts

Atkinson and colleagues [36] have recently shown that the basic vocabulary does not evolve gradually but shows bursts of rapid change following language splits. Essentially, the amount of evolution on the path leading from the root of the tree to a language is positively correlated with the number of nodes (splits) on the path. Using a complex methodology which controls for phylogenetic relatedness and the so-called “node-density” artifact [37] in three language families (Indo-European, Bantu and Austronesian), they find that between 9.5% and 33% of the vocabulary change is due to punctuational bursts around splitting events [36]. Here we use a much simpler method to explore the possibility that structural change might also follow a punctuational model by computing the correlation between path length and the number of nodes (Methods Section: **Punctuated evolution**).

We found that across all language families and datasets, the correlation between path length and number of nodes is very high (range 0.65–0.80, mean = 0.75, sd = 0.046), suggesting that punctuational bursts might explain about 50% of structural change. There are large differences between language families and datasets (**Materials S1**) with most families showing a positive correlation (range −0.66–0.87, mean = 0.37, sd = 0.32; one-sample t-test comparing to 0: 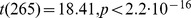). We also estimated the strength of punctuated evolution for different categories of linguistic features for the four datasets using Harald Hammarström’s classification and found important punctuational effects for all categories (on average on the order of 25%), and small but significant differences between them (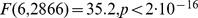 across all families). *Phonology* and *Morphology* show the lowest punctuational effects (on the order of 20%), while *Nominal Categories*, *Word Order* and *Simple Clauses* show the biggest effects (on the order of 35%); see **Materials S1**. When estimating punctuated evolution for each category in each family (**Materials S1**), we discovered quite extensive variation between categories across families (the interaction between family and category is highly significant, 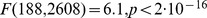), but all categories tend to show consistent punctuational evolution in all families (one-sample *t*-tests comparing each category across families to 0 are highly significant, 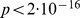). Interestingly, the strongest punctuation is shown by the largest families and, while this could be entirely an artifact of better sampling and branch length estimation, it might also suggest that large and small families evolve through different processes. Thus, within the limits of this method, our data suggest that structural features also evolve in punctuational bursts around language splits.

### The Relationships between Stability Profiles Suggest Universal Tendencies in Structural Stability

**Figure 1:**
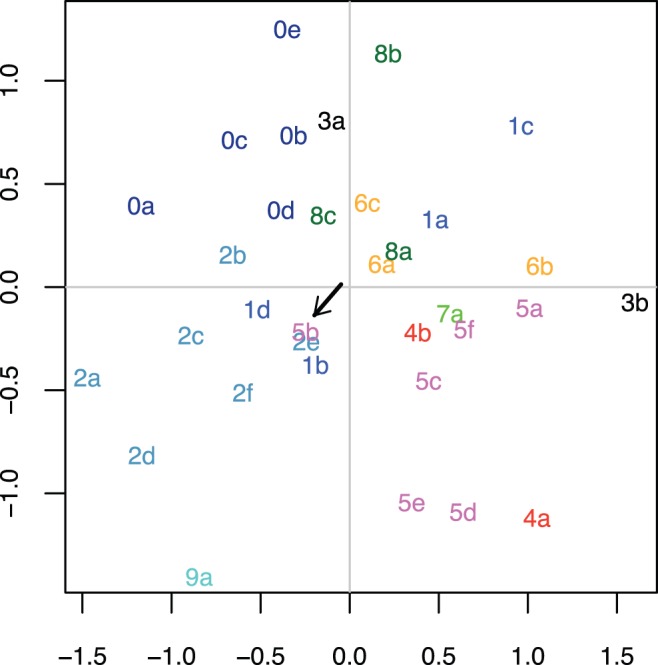
The *stability hyper-cube* for two features 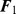 and 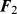, the *stability profiles* of three language families 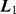, 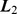 and 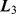 and the *stability distances* between language families (shown for 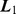 and 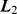). Please note that 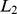 and 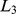 are very close in this space.

As explained in detail in **Materials and Methods**, the *stability profile* of a language family captures the stabilities of a set of structural features during the evolution of that family. This stability profile can be visualized as a point in a multi-dimensional *stability hyper-cube* (see Figure 1 and Methods Section: **The stability profile of a language family**) determined by the features considered. In any given dataset there are several language families, and for each family we computed its stability profile, representing all the features’ stabilities in this family. One such profile can be visualized as a point in the multi-dimensional stability hyper-cube determined by the structural features considered in the dataset, and the profiles of all families in the dataset form a cloud of such points.

The “shape” of this multi-dimensional cloud contains information about the relationships between the stability profiles of the language families considered (Methods Section: **The “shape” of stability profiles**), in the sense that a “compact” (“clustered”) cloud points to similar stability profiles, a “dispersed” cloud suggests dissimilar profiles, while a “random” one indicates a possible lack of relationships between these stability profiles. To investigate this multi-dimensional shape, we adapted two techniques from the analysis of spatial point patterns [38] (Methods Section: **The “shape” of stability profiles**). Please note that we use “spatial” to refer to abstract multi-dimensional mathematical spaces, reserving “geographical” or “geography” for the real space in which populations speaking languages evolve and interact.

First, we compared the shape of the relationships between the stability profiles of the language families to those expected from a random distribution, and found that the stability profiles across language families are much more similar (more clustered in the stability hyper-cube) than expected by chance (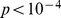). We replicated this by generalizing Ripley’s 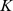 function [39] to the multi-dimensional stability hyper-cube (Methods Section: **The “shape” of stability profiles**). This generalized Ripley’s *K* function compares the properties of the observed stability profiles to those of an equivalent cloud of points generated by a random Poisson process, and determines the nature of its non-randomness (clustered or dispersed) and its associated significance. Using this, we strongly rejected the null hypothesis of *complete spatial randomness* [38], with 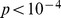 in favor of very strong clustering of stability profiles (**Materials S1**). Thus, the stability profiles are clumped together in the stability hyper-cube, showing that the stability profiles of the language families involved are much more similar than expected by chance. This suggests that there is a *strong universal component* of the structural stability of languages, manifested as an intrinsic, language family-independent tendency for structural features to systematically differ in their relative stability.

This finding supports and complements our earlier results [9], obtained using a different methodology for comparing the stability of structural features across language families. The consensus ranking among the 12 datasets of these features, from the most stable to the most unstable, is given in **Materials S1** (see also [9]), and the top and bottom 15 are given in Table 1. Work in progress involving the first author (Dediu, D. & Cysouw, M. *in preparation*, Some Structural Aspects of Language are More Stable than Others: A Comparison of Seven Methods), comparing seven diverse methods of conceptualizing and estimating the stability of structural features from the linguistic typological literature (including [9]), concludes that they all agree in finding that some features tend to be more stable than others (see Section **Comparing structural stability across methods**).

**Table 1:** Top and bottom 15 most stable features.

Rank	Polymorphic features
1	Absence of Common Consonants
2	Front Rounded Vowels
3	The Optative
4	Vowel Nasalization
5	Obligatory Possessive Inflection
6	Order of Genitive and Noun
7	N-M Pronouns
8	Nominal and Locational Predication
9	Uvular Consonants
10	M-T Pronouns
11	Order of Object and Verb
12	Order of Numeral and Noun
13	Numeral Classifiers
14	Order of Subject and Verb
15	Tone
…	…
54	Locus of Marking in the Clause
55	Voicing in Plosives and Fricatives
56	Symmetric and Asymmetric Standard Negation
57	Applicative Constructions
58	Relationship between the Order of Obj. and Verb and the Order of Adj. and Noun
59	Order of Person Markers on the Verb
60	Indefinite Articles
61	Asymmetrical Case-Marking
62	Definite Articles
63	Third Person Pronouns and Demonstratives
64	Position of Polar Question Particles
65	Number of Cases
66	Ordinal Numerals
67	Consonant-Vowel Ratio
68	Consonant Inventories

This ranking represents the consensus among all 12 datasets as given by the first principal component (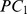) of a Principal Component Analysis run on all polymorphic ranks, explaining 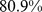 of the variance and representing the agreement. See **Materials S1** for details and WALS [31], [32] for the description of the features.

### The Stability Profiles also Show Patterns of Similarity among Language Families

The stability hyper-cube is a high-dimensional space (having between 68 and 86 dimensions depending on the number of features considered) and, in order to visualize on paper the relationships between stability profiles of the language families in these spaces, we used *multi-dimensional scaling* (MDS; [40]; a technique for projecting distance matrices on a space with lower dimensions with minimal distortions) and *networks* (using Neighbor-Net [41] as implemented in the SplitsTree4 [42]; a method for representing a space of probable but partially conflicting trees). We stress that both the MDS plots and the networks are used here simply as visual representations of the multi-dimensional relationships between the stability profiles, and we emphatically warn against automatically interpreting these networks in a phylogenetic manner. Similar (neighbouring) stability profiles could be a result of multiple factors, including descent from a common ancestor, contact and borrowing, chance, or various types of constraints on language change.

**Figure 2:**
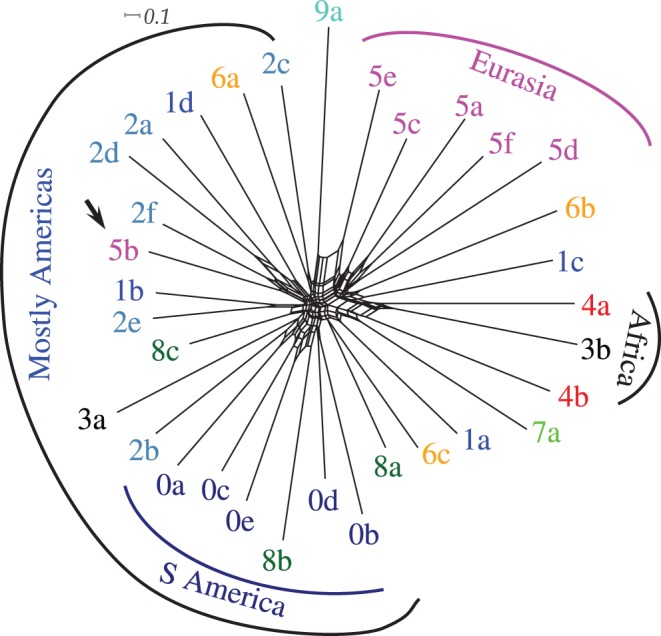
Multidimensional scaling (MDS) plot of the relationships between the stability profiles of the language families for the MBE dataset. Shown are the first (horizontal) and second (vertical) dimensions. We distinguished ten geographical regions represented by a distinct color and single digits, as follows: *South America* (**0**, dark blue), *Central America* (**1**, blue), *South America* (**2**, light blue), *Southern Africa* (**3**, black), *Northern Africa* (**4**, red), *Eurasia* (**5**, pink), *South Asia* (**6**, orange), *Oceania* (**7**, green), *Papua-New Guinea* (**8**, dark green) and *Australia* (**9**, cyan). The language families are represented by single lower case letters allocated in alphabetical order per geographical region, as follows: *Arawakan* (**0a**), *Carib* (**0b**), *Macro-Ge* (**0c**), *Tucanoan* (**0d**), *Tupi* (**0e**), *Chibchan* (**1a**), *Mayan* (**1b**), *Oto-Manguean* (**1c**), *Uto-Aztecan* (**1d**), *Algic* (**2a**), *Hokan* (**2b**), *Na-Dene* (**2c**), *Penutian* (**2d**), *Salishan* (**2e**), *Wakashan* (**2f**), *Khoisan* (**3a**), *Niger-Congo* (**3b**), *Afro-Asiatic* (**4a**), *Nilo-Saharan* (**4b**), *Altaic* (**5a**), *Chukotko-Kamchatkan* (**5b**), *Dravidian* (**5c**), *Indo-European* (**5d**), *North-Caucasian* (**5e**), *Uralic* (**5f**), *Austro-Asiatic* (**6a**), *Sino-Tibetan* (**6b**), *Tai-Kadai* (**6c**), *Austronesian* (**7a**), *Sepik* (**8a**), *Trans-New-Guinea* (**8b**), *West-Papuan* (**8c**) and *Australian* (**9a**). It can be seen that most of the American language families are distinguished from the others by the first dimension (left side) respecting the north (bottom) - south (top) geographic direction as well (second dimension). *Eurasia* occupies the bottom-right quadrant while *South Asia* and *Oceania* group together as well. Interestingly, *Chukotko-Kamchatkan* (**5b**; marked with a black arrow) clusters with the (Central and North) American language families. See supplementary figures in **Materials S1** for all 12 datasets.

**Figure 3:**
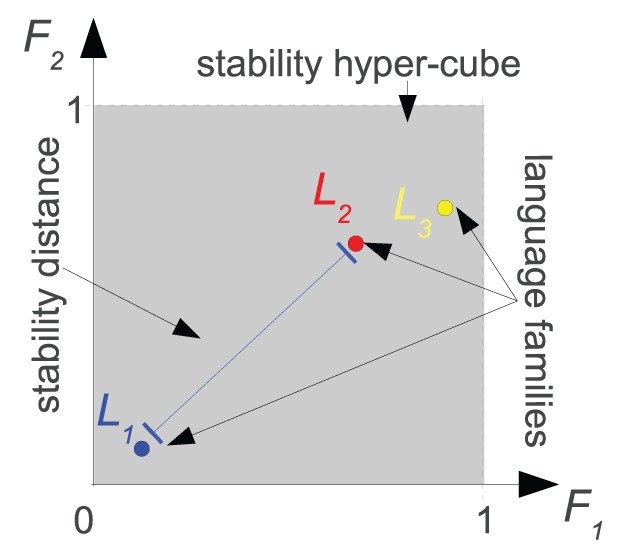
Network representation of the relationships between the same stability profiles as in Figure 2 (same conventions apply). Same clusters as in Figure 2 can be observed but the attachment of *Chukotko-Kamchatkan* (**5b**; marked with a black arrow) is now clearer with the North American families *Algic* (**2a**), *Penutian* (**2d**), *Wakashan* (**2f**), and the Central American *Uto-Aztecan* (**1d**) whose geographical range, in fact, extends well into North America. See supplementary figures in **Materials S1** for all 12 datasets.

Both methods reveal the existence of striking patterns of variation across language families, showing *a priori* unexpected *geographic clusters* (see Figures 2 and 3 illustrating the same dataset, and **Materials S1** for all 12 datasets): the American language families tend to group together along geographic lines (South, Central and North groups) and the North-Eastern Eurasian (Siberian) language families are attracted to the American cluster (Figures 2 and 3, black arrow). Weaker tendencies to clustering are also shown by Eurasian (except for North-East), and African (except *Khoisan*) language families. Interestingly, Australian and Papuan languages are very distanced from each other. *Khoisan* and Australian families are outliers, away from all the other families.

These patterns are striking as there is no *a priori* reason why the *stability profiles* of language families, as opposed to patterns of feature values, should be similar in such a way. In order to understand these patterns, (a) we tested the relationship of stability profiles with geography, (b) we tried to identify the structural features most responsible for these clusters, and, (c) we tested the internal consistency of the clusters in an attempt to rule out false positives. Finally, reassured that these clusters are robust, we went on to check if this patterning of the stability profiles supports some of the proposed macro-families in the linguistics literature. We describe these procedures in turn.

### These Patterns Seem to have a Geographic Component

Globally, there are weak to moderate but significant Mantel correlations [43] between the similarity of the stability profiles of language families and their geographical closeness (Methods section: **Geographic distances between language families**): 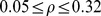, 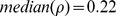, combined 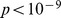 (**Materials S1**). This suggests that language families closer geographically also tend to have similar stability profiles. Such a positive relationship between geography and stability points to a weak or moderate role played by geographical distance in shaping the stability profiles of language families. Geographical distance would play, in this case, a role of proxy for other causal factors, such as language contact, as this is, in general, facilitated by geographical closeness. However, genealogically related languages also tend to be in close geographical proximity due to mechanisms of language expansion and differentiation. (Another possibility could be represented by systematic biases in the coding of structural features in WALS, reflecting geographically-based traditions and theoretical stances, but we believe this possible source of artifacts to be negligible given that the stability profiles are abstract constructs resulting from complex inferential processes using the whole structural information on groups of related language).

One approach to understanding this positive relationship between stability profiles and geographic distance is to find out which subsets of structural features maximize it. If only very stable features are required then the relationship likely reflects deep events, while very unstable features might point to recent phenomena. We used a genetic algorithm-based search (Methods section: **Features maximizing the correlation between stability and geographic distances**) and we found that, in general, a small subset of 10 to 18 features are required to maximize this correlation. These features include both very stable and very unstable ones and tend to differ among datasets (**Materials S1**), suggesting that probably a combination of both ancient and more recent phenomena plays a role.

### Statistical Robustness of Supra-family Patterns

Finally, we tested the statistical robustness of the groupings suggested by the MDS plots and networks, on one hand, and by the existing literature on deep relationships between established language families, on the other, using a permutation approach (Methods section: **Testing the robustness of groups of language family**). This method compares the properties of the stability profiles of an observed subset of language families of interest (say, a particular proposal for “Nostratic”, a suggested macrofamily including various Eurasian language families such as Indo-European and Uralic [44]) to the properties of 10,000 randomly permuted subsets of the same size chosen from the whole (or part of the whole) set of families. For each subset of interest we performed this permutation-robustness test in each of the 12 datasets, obtaining 12 empirical (“permutation”) *p*-values. Each of these 12 *p*-values indicates the probability that the properties of the stability profiles of the language families included in the subset of interest are “special” relative to random assemblages of language families from the larger set. Thus, *p*-values smaller than an *a priori* agreed 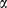-level (usually 0.05) indicate that the subset of interest is “special” with regard to the language families in the corresponding dataset. “Special” in our case here means simply more clustered in the stability hyper-cube (the language families included are more similar in their stability profiles) than expected by chance *in the context* of the dataset. This provides a method for testing whether a “macro-family” proposal is supported by the particular patterns of retentions and losses of structural features in the cluster of families being tested. Given the positive influence of geographical closeness, and thus typological diffusion by contact, we also controlled for it by comparing the clustering of the considered subset of language families in the stability hyper-cube with that expected for a random equivalent subset separated by the same geographical distances.

Thus, for each subset of interest we performed the same statistical test (our permutations-based test of robustness) 12 times on the 12 different datasets. Clearly, these 12 data-sets are not independent measures, so the standard meta-analytical statistical tools for combining *p*-values [45], [46] cannot be used *a priori*. Nevertheless our 12 data-sets do not correlate perfectly either, requiring a more refined approach to combining their *p*-values, described extensively in Methods section: **Combining** ***p*** **-values from non-independent experiments**. In summary, we combined these empirical *p*-values from the 12 datasets using five methods, conservatively taking the *largest p*-value for the subset of interest to guard against false positives (see Tables 2 and **Materials S1**). We will also report the number of methods (out of all 5) for combining *p*-values that result in a significant result at the 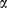-level of 0.05.

**Table 2:** Statistical robustness of sets of language families.

Set of families†	Raw		Controlling for geography	
	Most conservative	Number signif.	Most conservative	Number signif.
Africa	0.074	3	0.39	0
**America**	**0.0003**	**5**	**2.69 · 10** ***^−^*** ^**8**^	**5**
**S America** (vs world)	**0.0054**	**5**	**0.00018**	**5**
**S America** (vsAmerica)	**0.049**	**4**	**1.20 · 10** ***^−^*** ^**9**^	**5**
C America (vs world)	0.38	0	0.90	0
C America (vsAmerica)	0.99	0	0.96	0
N America (vs world)	**0.018**	**5**	0.072	2
N America (vsAmerica)	0.12	3	**4.61 · 10** ***^−^*** ^**10**^	**5**
**America + Siberia** ‡	**0.00022**	**5**	**0.00096**	**5**
**S America +** **Siberia**	**0.02**	**5**	**0.014**	**5**
C America + Siberia	0.37	0	0.42	0
**N America +** **Siberia**	**0.00039**	**5**	**0.034**	**4**
Eurasia	**0.036**	**5**	0.70	3
**Core Eurasia**	**0.0013**	**5**	0.094	**4**
Nostratic v1	**0.011**	**5**	0.13	3
Nostratic v2	0.24	0	0.77	0
SE Asia + Oceania	0.48	0	0.83	0
Austro-Tai	0.070	3	0.12	3
PNG	**0.042**	**4**	0.22	0
Australia	0.42	0	0.51	0
PNG + Australia	0.87	0	0.99	0

The most *conservative* combined *p*-value and the *number* of combined *p*-values significant at 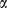-level = 0.05 for the five methods (Fisher, Z-transform, Hartung, Simes and Makambi) as applied to all 12 datasets for raw and geography-corrected stability distances. The combined *p*-values significant at 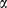-level = 0.05 are in **bold**). The sets with at least 4 significant combined *p*-values in both the raw and geography-corrected columns are also in **bold**. See **Materials S1** for full details.

† See **Materials S1** for the exact composition of these sets. **(vs America)**: randomization only within the Americas. **(vs world)**: randomization not restricted.

‡ Here we report the results for the maximal composition of “Siberia”, namely *Chukotko-Kamchatkan*, *Tungusic* and *Yukaghir* (the results are very similar when excluding *Tungusic*). See text and **Materials S1** for details.

### Some Patterns Suggest Possible Ancient Relatedness

The results are intriguing and could provide support for some proposed macro-families on a large scale. The permutation test found that the stability profiles of the American language families are much more similar than expected by chance (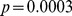) and this holds even after controlling for geography (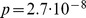), a result found using all 5 methods for combining *p*-values; Table 2 (please note that as discussed in the Methods section, most cases where controlling for geography results in a much lower *p*-value, are artifacts of our conservative approach of picking the highest combined *p*-value). Moreover, South American families also form a coherent sub-group (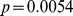; 5 methods) even after controlling for geography (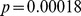; 5 methods), while North American families form their own subgroup only when not controlling for geography (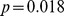, 5 methods and 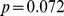, 2 methods, respectively). Importantly, the Siberian language families (comprising *Chukotko-Kamchatkan*, *Tungusic* and *Yukaghir*; see **Materials S1**) group robustly with the Americas (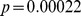, 5 methods and, after taking geography into account, 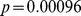, 5 methods). In particular, Siberia clusters especially with North America (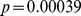, 5 methods and 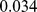, 4 methods after controlling for geography) and with South America (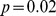, 5 methods, and 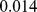, 5 methods when controlling for geography).

Africa shows a suggestion of forming a coherent group (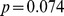, 3 methods), but this evaporates when controlling for geography (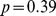, 0 methods).

Probably the best known proposal for a macro-family is represented by the various versions of Nostratic (see [44] for a critical assessment) covering several Eurasian and North African language families. We found no evidence for a version of Nostratic comprising *Afro-Asiatic*, *Indo-European*, *Dravidian* and *Uralic* (“Nostratic v2” in Table 1; 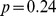, 0 methods, and 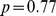, 0 methods, when controlling for geography), but there is a positive indication for another version of Nostratic comprising *Altaic* (or *Mongolic* + *Turkic*), *Indo-European* and *Uralic* (“Nostratic v1” in Table 1; 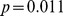, 5 methods, and 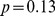, 3 methods, when controlling for geography). Interestingly, a comparable indication seems to hold for the whole of Eurasia (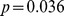, 5 methods, and 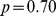, 3 methods, when controlling for geography). Quite convincing is the evidence that Core Eurasian families (comprising *Altaic* – or *Mongolic* + *Turkic* –, *Dravidian*, *Indo-European*, *Uralic* and the Caucasian families) might form a group (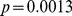, 5 methods, and 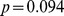, 4 methods, when controlling for geography).

There is a weak signal characterizing the set of so-called ‘Papuan’ families, where ‘Papuan’ just means non-Austronesian languages in the greater New Guinea areas (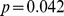, 4 methods, but not supported by any method after controlling for geography). Moreover, there is no evidence at all for Australia forming a coherent cluster, nor for groupings such as Papuan + Australian, and South-East Asian + *Austronesian*.

Finally, Reid’s [47] controversial proposal suggests that the *Tai-Kadai* and *Austronesian* language families are related forming the *Austro-Tai* group; we found a weak suggestion for this hypothesis (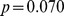, 3 methods, and 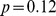, 3 methods, when controlling for geography).

## Discussion

The findings presented here strongly support the existence of a universal tendency across language families for some specific structural features to be intrinsically stable across language families and geographic regions, as previously reported by the first author [9]. One implication is that the most stable structural features of languages could be useful for deep historical reconstruction just like the most conservative portion of the vocabulary. However, one potential issue is that structural features have a much more limited set of possible states than the vocabulary, possibly leading to faster saturation (exploration of the possible states), and corresponding loss of phylogenetic signal. While this might indeed seem to theoretically limit structure-based investigations to shallower timedepths than those based on the vocabulary, much depends on rates of change of structure vs. vocabulary. Clearly, taken as a whole, vocabulary changes at much faster rates than structure (we can all recognize changes in our own lifetimes; see [48]). This is why vocabulary methods usually restrict themselves to the most conservative core of the lexicon, although there are important exceptions [48], [49]. In contrast, recent work by Dunn and colleagues [7], [50] suggests that on average a particular word-order change, for example, occurs just once in tens of thousands of years of evolution within a language family. As we have shown here and in [9], structural features also differ in their stability, some being labile, some highly conservative. We have also shown that this scale of stability has both universal and more locally restricted versions, all of which can be exploited judiciously for the exploration of deep historical relationships between languages.

Another problem that might plague phylogenetic reconstructions based on structural features is represented by the fact that they can be affected by horizontal processes such as borrowing [8]. Of course, language contact affects all components of language [51], especially vocabulary, and while vocabulary lists selected for conservatism (such as the versions of the Swadesh list) might be more resistant to it than the rest of the vocabulary, they are certainly not immune [48], [52]. There are significant misunderstandings of the role of contact in linguistic phylogeny, as pointed out in [50]: changes, whatever their source, will still be reflected in the phylogenetic profiles of language families, so the borrowing of structure should not fundamentally undermine the inference of phylogeny. In fact, recent simulation studies [53], [54] support the idea that phylogenetic inferences are robust to the degree and type of horizontal processes affecting language. When estimating rates of change in a phylogenetic framework – as done here – *any* source of change affecting language structures will count. Thus, if a feature is easily borrowed, these changes will be detected exactly as if determined by other causes of language change. Also, we find that the stabilities estimated by our phylogenetic method accord very well with those estimated by methods that explicitly model horizontal processes in language. More fundamentally, we believe that the manner in which horizontal processes in language are treated reflects deep philosophical questions concerning the historical processes and the nature of the entities whose history is reconstructed, in a manner parallel to the current controversy surrounding horizontal genetic transfer and the status of the Tree of Life in evolutionary biology [55]–[57].

The method proposed here attempts to take into account these issues (i) by considering a *large number* of structural features covering diverse aspects of language, (ii) by using *Bayesian phylogenetic* methods which can partially incorporate the uncertainty generated by horizontal processes into the posterior distributions, and (iii) by focusing on *higher-order properties of the evolutionary dynamics of patterns* of structural features.

While supporting the case for a core set of stable structural features across language families, our approach also reveals that the residual differences in structural stability between families can carry a historical signal that may be used to throw light on human prehistory. We found that the stability profile of a language family carries a signal reflecting both its deep genealogical relationships and its areal membership. Controlling for geography removed about half of the higher-level clusters of language families we found, suggesting that this similarity between stability profiles is not fully explained by contact phenomena, leaving as primary explanation the persistence of deep genealogical relationships. However, factoring out geography is also likely to factor out some genuine genealogical relations, since in a model of language diversification driven by population splits, related languages (and later, families) will also stay close in geographical space, confounding geography and underlying phylogeny. Moreover, this geographical closeness also promotes borrowing across sub-lineages, promotes language shifts, standardization, etc. As previous research on deep historical relations between languages has noted [16], structural profiles of languages can reflect both deep phylogeny and ancient contact. Supporting this dual contribution is our finding that the positive correlation between stability profiles and geographic distances is maximized by a subset of features containing both stable and unstable structural features.

Whatever the actual relative contribution of horizontal and vertical processes in shaping the patterning of language family stability profiles, it seems that these profiles are able to conserve ancient connections between language families. While it is well-known that values of structural features show geographic patterning due to vertical and horizontal processes, we have shown here that, abstract stability profiles are also geographically patterned, probably preserving a signal of much older or larger-scale such processes. For example, the strong clustering of the Americas and the Siberian languages fits the general migration patterns inferred from archeology and genetics [58]. The recent proposal of the linguistic affiliation of the *Yenisean* languages of Siberia and the *Na-Dene* languages of North America [59] could represent a potentially more recent linguistic example. In support of our method is the finding that while the whole of the Americas, and within it, North and South Americas form clusters, Central America – a well-known linguistic area [60] – does not, suggesting that the method is not overtly sensitive to relatively recent horizontal processes. It is important to note that very different approaches using the distributional patterns of structural linguistic features have recently suggested that the Americas share certain such features [61], and that it might even be a member of a putative linguistic area encircling the Pacific [62]. This suggests that stability profiles can reveal ancient connections, perhaps in this case dating back to the original peopling of the Americas at least 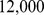 [63] years ago. Our findings provide some weak indication for a grouping within Papua-New Guinea, and cannot reject the *Austro-Tai* hypothesis. The lack of similarity between Papuan and Australian languages seems to suggest distinct demographic events taking place before or after the breaking up of the Sahul [64] and eroding any signal of relatedness. Finally, we did find support for one version of Nostratic, and for a Core Eurasian set of language families. Also, the whole of Eurasia received some support as a grouping of language families. Thus, our method seems to suggest some ancient connections between the Eurasian language families on one hand, and the American families on the other, but it is unclear if these connections reflect ancient genealogy or contact phenomena.

We believe that there is no contradiction between our findings here that the pattern of inter-language family variation in the higher-order stability profiles has three components (universal, language family-specific and genealogical/areal) and work suggesting that there are no language universals in general [17], [65] or typological implicational universals in particular [7]. More precisely, our universal tendencies for some structural features to be more stable than others across language families (see also [9]) are just that: *statistical tendencies* far from rigidly dictating the exact ranking of the features in any particular language family. These tendencies could result from “soft” cognitive, articulatory or auditory constraints or biases [25], [66] and/or emerging properties of languages as evolutionary cultural systems whose main function is complex communication. It is even possible that these “universal” tendencies reflect the ultimate monogenesis of language rather than persistent constraints, but this would require a very high conservatism of the stability profiles. The recent finding [7], [50] that constraints on syntactic change have a lineage-specific character is also consistent with the idea of stability profiles reflecting underlying genealogy, although one may expect more comprehensive studies of more language families to reveal some underlying commonalities.

Our preliminary finding here that structural features of language also show punctuated evolution like the basic vocabulary [36], and that different categories of features tend to be differently affected by punctuation across families could help shed light on the process of language divergence. Future work must investigate the causes for this variation between language families and categories of features in the importance of punctuation.

In conclusion, we found that the pattern of relative stability derived from multiple structural features has both a universal component and a genealogical/areal component. The universal component may offer insights into systems properties of languages in general, together with their contributing cognitive and genetic biases. The genealogical/areal component may offer a glimpse into ancient demographic and linguistic processes such as the peopling of the Americas, and promises some reach beyond the conventional time horizon of the comparative method in historical linguistics. In addition, comparative work on this higher, more abstract level of analysis may help to provide tools for more focused investigations of historical relationships within geographic areas: for in suggesting features that tend to be universally stable or stable within specific language families, this method may allow the judicious selection of structural features for more conventional phylogenetic analyzes of historical relationships. We hope that future work capitalizing on higher-order properties of languages seen as evolutionary systems will prove fruitful for a better understanding of language and its evolution.

## Materials and Methods

All analyzes reported here were conducted using the open source statistical environment R versions 2.13 and 2.14 [67].

### Primary Data

We used the same primary data (structural features and languages families) and methods for estimating the features’ rates of change as in [9], and, therefore, we will only briefly describe them here. To these, we added a new set of language families (described below), extending the datasets used in [9]. Moreover, we greatly extend and complement the analyzes presented there using a novel approach and methodology, and we enlarge the focus to the apportionment of variation *among* language families in addition to their shared, universal tendencies.

We collected structural data from the *World Atlas of Language Structures* (henceforth WALS [31], [32], available online at http://www.wals.info), and we filtered them by removing features with a high percentage of missing data and a low coverage in terms of the number of families [9]. The features in WALS have a number of values varying between 2 and 9 and some of these features could arguably be regarded as conflating two or more distinct aspects. Thus, to control for the effects of coding and study the behavior of such aspects separately, we coded the features as either *polymorphic* (the original rank-level coding from WALS; e.g., the feature *tone* has three values in WALS, namely “no tones”, “simple tone” or “complex tone”) or *binary* (linguistically informed recoding based on the WALS values; e.g., *tone* results in two binary aspects: *tone1* =  “no tones” versus any type of tone, and *tone2* =  “complex tone” versus “simple” and “no tones”). See **Materials S1** for the list of structural features used here, their description and the binary aspects (if any) and [9] for full details. It should be noted that, on top of the general issues concerning the comparability of typological categories across languages [18], WALS introduces several other difficulties. WALS does not provide the actual values for several features (such as the number of consonants or vowels in a language) but instead offers *ranked* summaries (such as languages with a “small”, “average” or “large” number of vowels), which artificially increases the homogeneity within such classes and the differences at the border between classes (i.e., a language with 4 vowels belongs to the “small” category but one with 5 to the “average”. Therefore, our results may depend on these characteristics of the WALS (which, with all its imperfections is currently the best available source of typological information with a large coverage both in terms of languages and features), but this must be left for future studies to assess.

Individual languages can be either *isolates* (such as *Basque* or *Ainu*) when no genealogical relationships with other languages can be established using historical linguistic methods, or they are classified as belonging to a *language family*, representing a genealogical grouping such as *Indo-European*. It has to be pointed out that the classification of languages into genealogical entities (language families) is a far from simple process and many disagreements persist as to the number, composition and internal structure of many language families. For some families (such as Indo-European) the agreement is greater than for others, while some are hotly debated (such as “Altaic”) or generally considered not to represent valid genealogical units (such as “Khoisan”) [2]. We avoided making such subjective judgments ourselves and instead took the “language families” as reported in several sources, each with its own characteristics. We collected such genealogical classifications of languages from three different sources: WALS [31], the Ethnologue [34] and Harald Hammarström’s appendix to [35], in order to control for the effect these classification might have on our results. The classifications offered by WALS and the Ethnologue are not independent and they mostly agree, but there are also slight differences, especially in what concerns the degree of specification of these genealogical trees. In both classifications there are entities with controversial status such as “Khoisan”, “Altaic” and “Australian” mostly rejected by orthodox historical linguists [2]. The classifications in WALS generally recognize only three levels (“Family”, “Genus” and “Language”), while Ethnologue recognizes as many as 14 levels and Hammarström’s 16. The language families collected by Harald Hammarström for his investigation into the language-farming co-dispersal hypothesis [35] follow several stringent criteria such as a “published demonstration” of their genealogical affiliation using the “orthodox comparative method” as described by Campbell and Poser [2]. There are no such entities as “Khoisan” or “Australian” present here. Details of these families, including their sources, are present in the appendix to [35] and a slightly updated electronic version of their structure was kindly provided to us by the author in January 2012. We used these electronic files to extract the tree topology for each language family. We allocated language families to 10 geographic areas (see Figure 2) loosely following WALS [31]. This allocation is mostly pragmatic, as it enhances the visualization and presentation of the results without impacting in any way on the actual process of hypothesis testing, which can consider arbitrary sets of language families, as described below. Details about the language families used, their structure and their allocation into geographic areas are given in **Materials S1** and Figures 1 and 2.

### Stability Estimation

For the inference of the features’ rates of change, we considered each language family as an independent given phylogeny with the feature values also given for the tips of this phylogeny (the extant languages). We used a Bayesian phylogenetic approach to estimating the rates of change. More specifically, to control for the effects of the specific method for estimating rates of change, we used two software packages, the widely used MrBayes 3 [33] and the custom-written BayesLang, specifically designed for the characteristics of this problem [9]. In general, Bayesian methods produce whole posterior distributions of parameter estimates (as opposed to single point estimates), and our procedure results in a distribution of estimated rates for each feature in the set of features for the considered language family. For MrBayes 3 we converted the language families into a set of constraints specifying the topology of the tree. The outgroup required by the software for rooting and rate estimation was represented in turn by each of a large set of language isolates selected for their feature completeness in WALS. With these, MrBayes 3 was used to infer branch lengths, ancient states and the rates of change for the features under investigation. Likewise, BayesLang does not require branch length but only a rooted tree topology represented by the language family. It also estimates branch lengths, ancient states and the rates of change for the features under investigation, with the difference that the rates represent the minimum number of changes required for the estimated ancestral state to result in the observed states given the evolutionary model assumed for the structural feature. This estimate is akin to a maximum parsimony model and was specifically chosen so that it uses a dissimilar method from MrBayes 3. For more details, please see [9]. Both MrBayes 3 and BayesLang share general assumptions such as the models of evolution on tree phylogenies and the computation of the likelihood of such phylogenies given the observed data, evolutionary models and their parameters [68], [69]. The main differences are that while MrBayes 3 was designed for biological datasets (and we treated the polymorphic features as morphological data and the binary features as restriction data), BayesLang was designed for the inference of the evolution of language structural data on fixed rooted tree topologies and it also accepts more refined (even user-defined) models of change for a given feature. Another difference discussed above concerns the type of rates estimated. Using these methods for linguistic structural data could induce certain biases. For example, treating linguistic structural data as restriction/morphological in MrBayes 3 might affect the estimation of rates, while the parsimony-like estimation in BayesLang could be affected by long branches. However, as detailed below, the high correlations between the results produced by these two software packages seems to suggest that these biases may not be important. Another possible issue, usually raised in relation to the application of phylogenetic methods to language, concerns the influence of not modeling the pervasive horizontal processes affecting language. However, as detailed in the Discussion, we believe that for this particular type of investigation, contact is implicitly included as yet another source of language change, contributing to the instability of the affected features.

With these, there are in total 12 *datasets*, each comprising a *software package* (**M**rBayes or **B**ayesLang), a data *coding* (**B**inary or **P**olymorphic) and a *genealogical classification* (**E**thnologue, **W**ALS or **H**ammarström). We will denote these datasets using the initial letters of the software package, data coding and genealogical classification: **MBE**, **MBW**, **MBH**, **MPE**, **MPW**, **MPH**, **BBE**, **BBW**, **BBH**, **BPE**, **BPW** and **BPH** (see **Materials S1**). Overall, we analyzed a total of 56 language families represented by 240 unique phylogenies composed of a total of 3836 languages, and 70 polymorphic and 86 binary features.

As explained in [9], to be able to compare these rates of change across language families and datasets without assuming calibration, we converted the *absolute rates* produced by the phylogenetic software packages to *standardized relative ranks* varying between 0.0 (most stable) to 1.0 (most unstable), as follows. For a posterior distribution of absolute rates (representing the results for a feature in a language family in a dataset), we extracted one by one each posterior observation of rates and ranked them (using the mean rank for ties); next, we normalized these ranks to the interval 0.1 as explained in detail below. For each of the 12 datasets, there is a set of structural features 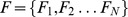 and a set of language families 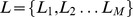 (for details see [9]). The application of MrBayes 3 or BayesLang to a particular language family 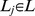 results in a large but finite sample (of size 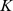) from the posterior distribution of *absolute rates* 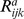, 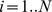, 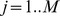, 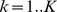, representing the 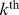 sampled absolute rate of feature 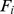 in language family 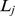. This is then converted to the relative rank sample, 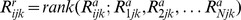, where 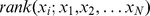 gives the rank of 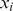 among the 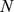 numbers (e.g., 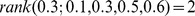). Further, these relative ranks 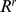 are standardized to 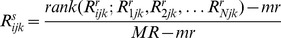, where 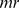 represents the minimum rank and 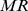 the maximum rank among 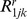, 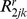, 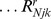. This standardized stability ranks distribution 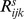 can then be summarized by its *mean* across the 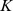 extractions, 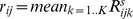, the *mean standardized stability rank* (but summarizing these standardized stability ranks distribution 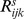 using the median produces similar results) of feature 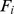 in language family 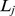. Thus, in the end we have the mean standardized stability ranks per feature, language family and dataset, representing the input data for the subsequent analyzes reported here.

Given the novel usage of Harald Hammarström’s [35] more “orthodox” classification here, it is important to quantify how well the stabilities estimated using it accord with those estimated using WALS and Ethnologue. To this end, we performed a Principal Component Analysis [70] on the rankings produced by the 6 binary and 6 polymorphic datasets separately. For both, the first principal component (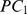) explains most of the variance(92.16% and 80.96% respectively) and represents the agreement between the two software packages and three linguistic classifications (all loadings have the same sign; see Table 1 and **Materials S1**). Thus, we confirm and extend the previous finding [9] that the relative stability of various structural features of language is conserved across methods and classifications.

### Punctuated Evolution

In order to estimate the existence and importance of punctuated evolution [37] on the structural features of language, we used a much simpler methodology than [36]. Our method is intended as an initial exploration of this topic, and is based on the principle that gradual and punctuated evolution result in different relationships between *path length* (the sum of the length of all branches connecting the root of the tree to a terminal node) and the *number of nodes* on the path: no correlation between the two for gradual evolution and a positive correlation for a punctuational process [71].

Given that the WALS classification limits the depth of trees to 3, we will focus here only on the Ethnologue and Hammarström’s classifications, resulting in 8 datasets (**BBE**, **BBH**, **BPE**, **BPH**, **MBE**, **MBH**, **MPE** and **MPH**). For each dataset and each posterior tree, we computed the correlation (Pearson’s *r* and Spearman’s 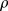) between path length and the number of nodes on the path for each terminal node (language) in the tree. For MrBayes the path length is the sum of the lengths of all branches composing the path, while for BayesLang the path length is computed as the total number of changes required to transform the root ancestral states for all structural values into the actually observed states in the terminal node (language). The two correlation coefficients used agree very well (**Materials S1**) such that we used only Pearson’s *r*. We computed the percent of variation explained by punctuational processes as the square of the correlation, 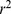.

For each of the seven categories of features as defined by WALS covered by our dataset (*Morphology*, *Nominal Categories*, *Nominal Syntax*, *Phonology*, *Simple Clauses*, *Verbal Categories* and *Word Order*) we estimated the punctuated evolution only for the four datasets using Hammarström’s classification (**BBH**, **BPH**, **MBH** and **MPH**) due to the high computational costs. Moreover, given that not all families cover all seven categories, we considered three cases defined by the set of families covering at least 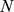 categories: 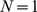 (all families), 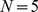 and 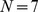 (only families covering all categories). We found similar results for these three cases, but 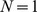 highlights the unreliability of estimating punctuated evolution for small families with poor coverage.

This simple method for estimating the role of punctuated evolution for the structural features of language does not control for shared ancestry among the languages of the same family nor does it shield against the “node-density artifact”, probably resulting in an inflated estimation of the contribution of punctuated evolution [36], [37], [71]. Therefore, these results should be taken as indicative, and more complex but also more time-consuming methods must be used to provide a better estimate of this effect. Nevertheless, given the large effects sizes found and their consistency across datasets and software packages (**Materials S1**), our estimates are most probably relatively accurate.

### The Stability Profile of a Language Family

Given a dataset, let us denote the mean standardized stability ranks of the structural features 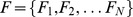 estimated for the language families 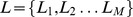 as 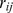. Given that 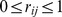, we can visualize each language family 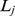 as a point in the 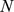-dimensional hyper-cube defined by the 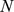 structural features 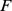, with coordinates 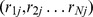. We call this 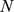-dimensional hyper-cube bounded by 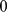 and 1 the *stability hyper-cube* and the coordinates of the language family 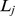 in this space as the language family’s *stability profile*. It should be noted that the concepts of stability hyper-cube and stability profile as defined above do not make any assumptions concerning the existence or not of universal tendencies, language family-specific or deep relationships between languages, but simply assume that language families can be compared with respect to the relative stability of a set of structural features in these families.

Given two language families, 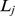 and 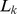, we computed the Euclidean distance between their stability profiles in the stability hyper-cube, 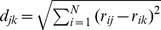 representing the *stability distance* between the two language families. The maximum possible stability distance between two families in an 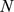-dimensional stability hyper-cube is 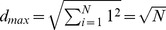.

To make things clear, let us consider just two features, 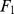 and 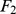 (say *tone* and *number of vowels*) and three language families 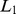, 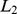 and 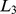 (say, *Indo-European*, *Uralic* and *Altaic*). Then the stability hyper-cube is, in fact, the 2-dimensional square of width 1 and the language families can be easily visualized as points in this plane (see Figure 1). The relative stability (mean standardized stability ranks) of feature 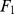 in family 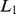 is 0.13, in 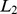 is 0.68 and 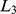 is 0.91 (the horizontal axis in the figure), while for feature 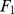 these stabilities are 0.10, 0.63 and 0.72, respectively (the vertical axis). The stability hyper-cube is the shaded area bounded by 0 and 1 on both axes and represents the theoretically possible stabilities these two features, 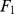 and 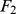, can have in any possible language family. The maximum possible stability distance in this case is 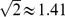. Families 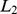 and 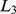 are grouped together, having a small stability distance between their stability profiles showing that they tend to have very similar stabilities for the features considered.

### The “Shape” of Stability Profiles

The stability profiles of the 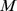 language families, 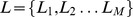, are a set of 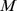 points in the *N*-dimensional stability hyper-cube. As opposed to a single stability profile, the “shape” of this cloud of points summarizes the pattern of stability across language families and holds important information concerning the existence of universal tendencies in structural stability. If the language families are *randomly scattered* then there is no universal, cross-language family component, supporting the view that stability is purely an idiosyncratic, language family-specific property. If they are more *clumped* (*clustered*) than expected, this would strongly suggest a universal component manifested as a tendency of structural features to have the same stability across families. If they are more *dispersed*, this would suggest a regular patterning of stability across families. We used two methods inspired from the analysis of point-patterns [38] to investigate the clustering, dispersion or randomness of the distribution of language families in the stability hyper-cube.

The first method involves generating 10,000 independent random sets of *M* points in the stability hyper-cube using a uniform distribution between 0.0 and 1.0 to generate the *N* coordinates for each of the *M* points, and comparing these random sets to the actually observed set of stability profiles. We used the distance to the *nearest-neighbor* and the *mean* distance between points as summary statistics for each set of *M* points (including the actually observed ones). We then compared the summary statistics of the observed set of stability profiles to the distribution of summary statistics for the 10,000 randomly generated sets to assess the clumping or dispersion of the actual data compared to the expected values. More precisely, we obtained an empirical *p*-value representing the proportion of random sets with smaller *nearest-neighbor* or *mean* distances than the actually observed set of stability profiles (**Materials S1**).

For the second method we generalized Ripley’s *K* function [39] to 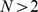 dimensions as follows. Given a set of points in a space, Ripley’s *K* is the average number of points within a radius 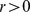 from a randomly chosen center divided by the density 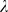 (the number of points per unit volume). An estimator of 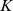 for a multi-dimensional point pattern is:
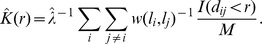
where 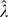 is the estimated density (
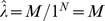
 in our case), 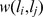 is a weight function implementing the edge correction (set to 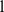 here; see below), 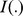 is the indicator function (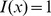 if *x* holds, 0 otherwise), and 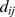 is the distance between points *i* and *j*. For the radius *r* we used 100 equal steps (or lags) between 0 and the maximum possible distance 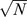. The edge correction (accounting for space “lost” due to the intersection between the spheres of radius *r* centered on the points and the stability hyper-cube’s limits) and the expected values of 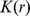 are not trivial to compute given the multi-dimensionality of the stability hyper-cube. Therefore, we estimated Ripley’s 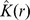 for 10,000 random Poisson processes in the same stability *N*-dimensional hyper-cube with the same number *M* of points, and we then compared the observed 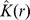 for the actual stability profiles to the distribution of these simulated 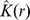
’s in order to assess the deviation of the actually observed data from the expected distribution under spatial randomness. This procedure results in empirical *p*-values (and confidence intervals) at each lag 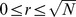 allowing the quantification of the deviation of the observed pattern from randomness (**Materials S1**).

### Comparing Structural Stability Across Methods

The question of the stability of structural features is an important one for historical linguistics and especially for linguistic typology and several approaches have been proposed in the literature. However, given the complexity of the processes affecting language change, there are many ways to conceptualize and operationalize stability. In order to understand these approaches and their relationships with each other, the first author together with Michael Cysouw (Dediu, D. & Cysouw, M. *in preparation*, Some Structural Aspects of Language are More Stable than Others: A Comparison of Seven Methods) are currently working on a systematic survey and comparison of 7 diverse methods from the linguistic typological literature.

The methods compared are:

- Cysouw and colleagues [19] consider the consistency of the cross-linguistic distribution of an individual feature with the pattern generated by multiple features, and they propose three quantifications of this measure based on Mantel’s correlation, a coherence and a rank method [19];
- Parkvall [20] proposes to distinguish features that tend to be vertically transmitted from those that are easily borrowable, quantified using the *Herfindahl-Hirschman* index (or *Gini coefficient*) computed across genealogical and areal units;
- Wichmann and colleagues, and especially Wichmann and Holman [22] have a predominantly phylogenetic conception of stability where a stable feature tends to be shared among related but not among unrelated languages;
- Maslova [21] proposes a relatively similar method based on estimating the probability of transitions between feature values;
- finally, the method described here [9] is a fully phylogenetic Bayesian approach to estimating the stability of structural features.

Interestingly, despite different concepts of stability and implementational approaches, these methods agree unexpectedly well (the first principal component of the feature rankings explains almost 50% of the variance and represents the agreement between methods). Thus, the stability captured by our method here seems supported by other approaches motivated from different perspectives.

### Geographic Distances between Language Families

Given two language families 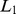 and 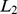, we computed the geographic distances between all pairs of languages from these families 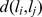, with 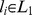 and 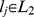 using great circle distances on Earth and forcing the paths to pass through way points between broad geographic regions. These way points are: “Syria” (lat: 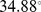, long: 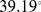; connecting Africa and Eurasia), “Bering Sea” (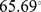, 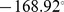, connecting North America and Eurasia), “Mexico” (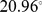, 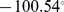, connecting North America and Central America), “Panama” (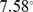,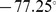, connecting South America and Central America), “Singapore” (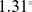, 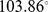, connecting Eurasia and Oceania & Papua-New Guinea), “Badu Island” (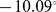, 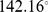, connecting Australia and Oceania & Papua-New Guinea).

Thus, for each pair of language families (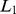,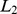) we obtained a set of geographic distances between all possible pairs of languages chosen from the two families. We summarized these using their *mean* 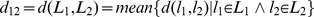 and took 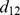 as the geographic distance between language families 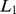 and 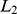. There are very high correlations between various summaries of these sets of distances between pairs of languages, 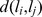, as shown by the Mantel correlations between them (we used 10,000 permutations when computing the *p*-values and all 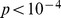): summarizing by the minimum and maximum distances between pairs of languages, 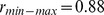; by minimum and mean, 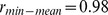; by minimum and median, 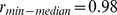; by maximum and mean, 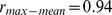; by maximum and median, 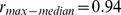; and by mean and median, 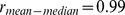). Thus, this justifies our choice of mean as a language-family level summary for geographic distances.

### Features Maximizing the Correlation between Stability and Geographic Distances

We searched for those subsets of features which maximize the Mantel correlation between stability and geographical distances, as follows. Let us consider 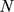 features 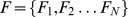 and 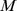 language families, 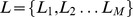. For any subset of 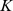 features 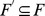 we computed the “restricted” stability profiles of the 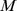 language families in the restricted stability hyper-cube defined by these 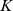 features, and the restricted stability distances between them. Then, we computed the Mantel correlation, 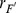, between the restricted stability distances and the geographical distances, as described above for the whole set of features 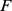.

We used a *genetic algorithm* (as implemented in the R package genalg 0.1.1) to search for the subsets 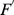 that maximize the Mantel correlation 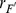 between stability and geographic distances. The genomes are binary of size 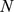 and one such genome represents a subset of features 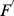 through its indicator function; thus “gene” *i* in this genome is 1 if and only if feature 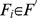 and 0 otherwise. The search used a population of 200 binary genomes, and was run for 500 generations. To insure generalizability, we replicated each search 5 times independently.

The search results in a set of 500 populations of 200 genomes (one population per generation), each of these 100,000 genomes having associated a value of the fitness function, in this case the Mantel correlation 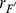 determined by the corresponding subset of features 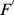. We defined a genome (subset of features) as being *optimal* if its fitness was equal to the maximum fitness for that particular run of the genetic algorithm; thus, in effect, an optimal subset is composed of features that maximize the Mantel correlation between stability and geographical distances. For each feature 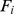, we defined its *involvement* as the proportion of times it appears in the set of optimal subsets; this varies between 0 (the feature does not appear in any optimal subset) to 1 (the feature belongs to all optimal subsets).

In general, the search process was very fast, reaching the optimal value of the Mantel correlation 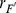 within the first 50 generations, after which it remained relatively stable. Within datasets, the 5 replicated runs produced remarkably similar results, as shown by the large first principal component (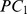 explains more than 73% of the variance in each dataset; see **Materials S1**) expressing the agreement between the feature involvements across the runs.

### Combining *p*-values from Non-independent Experiments

Our 12 datasets represent different combinations of software packages, codings and linguistic genealogical classifications, but they do not represent statistically independent experiments due to dependencies at several levels:

- the structural features and their values come form a single source, namely the WALS;
- the polymorphic and binary codings are meaningfully related;
- two of the genealogical linguistic classifications are not independent, as WALS was explicitly inspired by Ethnologue;
- the two software packages use the same fundamental mathematical and statistical apparatus (Bayesian phylogenetic inference).

Therefore, the information provided by these experiments is partly but not completely redundant.

There are several well-established methods for combining significance (*p*-value) and effect size information from *independent* tests of the *same* null hypothesis 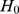, especially developed for meta-analyzes, such as:

- **Fisher**’s classic method [45], and the more recent
- Z-transform [46],

but *a priori* they are not appropriate to our case due to the mentioned non-independence.

Methods for combining *dependent p*-values, however, are not as well developed and have various assumptions which are not easily checked in real situations. Nevertheless, we selected three such methods from the literature and implemented them in R [67] (see **Materials S1** for the R code implementing them):

- **Hartung**’s [72] method assumes constant correlations across the tests and it also provides an estimate of this correlation;
- **Makambi**’s [73] is an extension of Fisher’s method for positively correlated dependent cases and assumes the homogeneity of the inter-test correlations; it also provides an estimate of this correlation; and
- **Simes**’ [74] approach is robust to dependence but it does not compute a combined *p*-value, instead testing if the null hypothesis can be rejected for a given 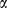-level by the combined information contained in the individual tests.

Using these five methods (a) – (e), we combined the one-sided *p*-values resulting from testing the same null hypothesis 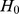 in the different datasets. As described in the main text and below, the null hypothesis specifically tested here concerns the stronger clustering of groups of language families as compared to an expected distribution derived by permutations.

All five methods agree very well on rejecting or not the null hypothesis at a conventional 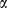-level of 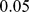, and the combined *p*-values (where available) correlate at over 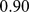 (**Materials S1**). The inter-dataset correlations estimated by **Hartung** and **Makambi** tend to be small to moderate (for **Hartung**: 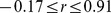, 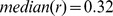, 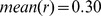, 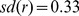; and for **Makambi**: 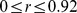, 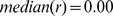, 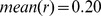, 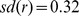) and strongly correlated between **Hartung** and **Makambi** (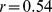, 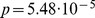). Thus, these estimates suggest that despite our justified *a priori* concerns, the dependencies between these 12 datasets are in fact small.

Nevertheless, we will take a *conservative* stance and report as the combined *p*-value the *largest* of the 4 *p*-values given by **Fisher**, **Z-transform**, **Hartung** and **Makambi**. Please note that this procedure, while guarding against false positives, does result in counterintuitive effects, such as the apparently dramatic lowering of the *p*-values when controlling for geography in some cases (for example, for America; see Table 2). However, these are artifacts due to the different assumptions of the methods for combining *p*-values, as can be clearly seen in **Materials S1**. Finally, given that we take this very conservative stance in combining the 12 datasets, we have decided to not correct for multiple comparisons. But even using an extremely conservative Bonferroni correction across all tested groups (see below) still results in, for example, the Americas forming a coherent group when controlling for geography (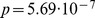), with Siberia still gravitating towards it for both the uncorrected (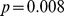) and geography-corrected (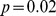) cases.

### Testing the Robustness of Groups of Language Families

In general, let us consider a subset of 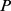 language families taken from the full set of families in the dataset, 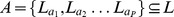 (thus, the indexes 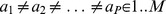). Such a subset 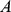 could be an *a priori* motivated grouping, such as a suggested macro-family, or a set defined *a posteriori* following some exploratory analyzes (such as from the analysis of the MDS and networks discussed previously), or it could simply be a random assortment of language families. We tested the *coherence* of such a subset 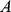 using a randomization approach as follows: we compared the observed geographic and stability distances between the language families in 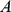 to those of random subsets of language families from 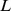 of the same size as 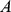 (namely, of size 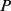).

More precisely, we considered the raw (i.e., uncorrected) and geographically-corrected mean stability distance between the language families. We generated 10,000 random subsets of language families 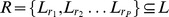 of the same length as 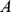, and we computed the proportion of such random subsets more extreme than 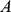, namely, having a smaller raw mean stability distance. This proportion represents the empirical *p*-value of the hypothesis that the language families in *A* form a group with stability profiles more similar to each other than expected by chance among the full set *L* of language families considered.

Next, we took the randomly generated subsets *R* and used them to infer what the mean stability distance between the families in *A* should have been if *A* were just another random subset of language families. More precisely, we regressed linearly the mean stability distance on the geographical distance between the language families in the random subsets (each random subset *R* represents a single data point in this regression) and we predicted the value of the mean stability distance given the observed geographical distance between the families in *A*. This tells us how the stability profiles in *A* should be related to each other for a set of families separated by the given geographical distance. Then we used the *prediction* 95% confidence interval of this regression to test the hypothesis (and derive a corresponding *p*-value) that the language families in *A* are more compact than expected by chance in *L* when controlling for geography.

Thus, the *uncorrected (raw) mean stability distance* tests the hypothesis that the language families in *A* have very similar stability profiles relative to the whole set of families, while the *corrected version* takes also into account the geographical distances between them. In most cases, the uncorrected *p*-values are smaller than the corrected ones (see **Materials S1**): the paired t-tests between the uncorrected and geography-corrected *p*-values are negative except, interestingly, for South, Central and North America versus America, in which case correcting for geography helps highlight the similarity within these areas against the background of the general similarity of the American families. However, it is not clear if the raw or corrected measures are more appropriate for our study, as they represent slightly different concepts of clustering in the stability space. More specifically, given that both areal (horizontal) phenomena (borrowing, language shift, etc.) and vertical genealogical relationships usually involve geographically neighboring populations, controlling for geographical distance might in fact remove an essential causal factor and not just a nuisance. Therefore, we have tested and reported both cases.

A main limitation of this method is its small power to test large subsets *A* from *L*, as there are few possible random subsets *R* equivalent to *A*. Therefore, we cannot test the coherence of larger sets of language families covering, for example, Eurasia and the Americas.

## Supporting Information

Electronic Supplementary Material S1 **Contains more information about the primary data and its coding (Tables S1, S3, S4 and S15), about the stability profiles (Tables S2 and S5, and Figures S1–S14), the involvement of features in the correlation between stability and geographic distances (Tables S6–S13), the combined** ***p*** **-values (Tables S14 and S16) and the R code implementing these methods (Table S17), and more results concerning the punctuated evolution of structural features (Tables S18 and S19, and Figures S15–S18).**
(PDF)
