# Correction: Abstract Profiles of Structural Stability Point to Universal Tendencies, Family-Specific Factors, and Ancient Connections between Languages

**DOI:** 10.1371/annotation/ceff8775-a4e3-45cb-b6c9-dd62d9179d59

**Published:** 2012-10-03

**Authors:** Dan Dediu, Stephen C. Levinson

There were errors in the placement of Figures 1-3. The published Figure 1 is actually Figure 2. The published Figure 2 is actually Figure 3. The published Figure 3 is actually Figure 1. The legends are in the correct locations.

