# Supplementary material for: Abstract Profiles of Structural Stability Point to Universal Tendencies, Family-Specific Factors, and Ancient Connections between Languages
Source: PLoS One. 2012 Sep 20;7(9):e45198. doi: 10.1371/journal.pone.0045198 (PMC3447929; doi:10.1371/journal.pone.0045198)
Supplement: Electronic Supplementary Material S1 — Contains more information about the primary data and its coding (Tables S1, S3, S4 and S15), about the stability profiles (Tables S2 and S5, and Figures S1–S14), the involvement of features in the correlation between stability and geographic distances (Tables S6–S13), the combined p -values (Tables S14 and S16) and the R code implementing these methods (Table S17), and more results concerning the punctuated evolution of structural features (Tables S18 and S19, and Figures S15–S18). (PDF) [file pone.0045198.s001.pdf]

Electronic Supplementary Material S1

Abstract profiles of structural stability point to universal tendencies, family-specific factors, and ancient connections between languages

Dan Dediu[1,3,*] and Stephen C. Levinson[2,4]

[1] Language and Genetics, Max Planck Institute for Psycholinguistics, Nijmegen, The Netherlands
[2] Language and Cognition, Max Planck Institute for Psycholinguistics, Nijmegen, The Netherlands
[3] Donders Institute for Brain, Cognition and Behaviour, Radboud University Nijmegen, The Netherlands
[4] Radboud University Nijmegen, The Netherlands

[*] Dan Dediu, E-mail: Dan.Dediu@mpi.nl

Summary

This **Electronic Supplementary Material (S1)** includes:

- more information about the *primary data* used in this paper and its coding (**Tables S1**, **S3**, **S4** and **S15**);
- the relationships between the language family *stability profiles* (**Tables S2** and **S5**, and **Figures S1-S14**);
- the *involvement* of different features in sets maximizing the correlations between geographic and stability distances (**Tables S6-S13**);
- the *combined p-values* (**Tables S14** and **S16**);
- the *punctuated evolution* of structural features (**Tables S18** and **S19**, and **Figures S15-S18**); and
- the *R code* implementing the five methods for combining *p*-values (**Table S17**).

The order of these items largely reflects the structure of the paper's main text.

Table of Contents

Supplementary Figures

Figure S1: Observed Ripley's ***K*** for **`BayesLang`** 5
Figure S2: Observed Ripley's ***K*** for **`MrBayes`** 6
Figure S3: Stability distances (MDS and NeighborNet) for **MBE** 7
Figure S4: Stability distances (MDS and NeighborNet) for **MBW** 8
Figure S5: Stability distances (MDS and NeighborNet) for **MBH** 9
Figure S6: Stability distances (MDS and NeighborNet) for **MPE** 10
Figure S7: Stability distances (MDS and NeighborNet) for **MPW** 11
Figure S8: Stability distances (MDS and NeighborNet) for **MPH** 12
Figure S9: Stability distances (MDS and NeighborNet) for **BBE** 13
Figure S10: Stability distances (MDS and NeighborNet) for **BBW** 14
Figure S11: Stability distances (MDS and NeighborNet) for **BBH** 15
Figure S12: Stability distances (MDS and NeighborNet) for **BPE** 16
Figure S13: Stability distances (MDS and NeighborNet) for **BPW** 17
Figure S14: Stability distances (MDS and NeighborNet) for **BPH** 18
Figure S15: Punctuated evolution across families 64
Figure S16: Punctuated evolution across categories of structural features 65
Figure S17: Punctuated evolution: means across families and categories 66
Figure S18: Punctuated evolution: standard deviations across families and categories 67

Supplementary Tables

Table S1: The 12 datasets 3
Table S2: Observed versus expected stability distances 4
Table S3: The structural features and their coding 22
Table S4: The composition and structure of the language families........ 36
Table S5: Mantel correlations between stability and geographic distances 37
Table S6: Most involved features for dataset **MBE** 38
Table S7: Most involved features for dataset **MBW** 39
Table S8: Most involved features for dataset **MPE** 40
Table S9: Most involved features for dataset **MPW** 41
Table S10: Most involved features for dataset **BBE** 41
Table S11: Most involved features for dataset **BBW** 42
Table S12: Most involved features for dataset **BPE** 43
Table S13: Most involved features for dataset **BPW** 44
Table S14: Correlations and concordances between methods for combining *p*-values 45
Table S15: The composition and interpretation of sets of language families........ 54
Table S16: Statistical robustness of sets of language families........ 56
Table S17: The **`R`** code implementing the methods for combining *p*-values 59
Table S18: Punctuated evolution across language families 61
Table S19: Punctuated evolution across categories of structural features 59

Dataset	Software	Coding	Classification	Structural features	Language families	Languages
**MBE**	MrBayes	Binary	Ethnologue	86	33	320
**MBW**			WALS	86	25	255
**MBH**			HH	86	38	459
**MPE**		Poly	Etnologue	70	34	319
**MPW**			WALS	68	18	162
**MPH**			HH	74	39	420
**BBE**	BayesLang	Binary	Ethnologue	86	39	303
**BBW**			WALS	86	26	266
**BBH**			HH	86	38	458
**BPE**		Poly	Ethnologue	70	28	195
**BPW**			WALS	70	25	249
**BPH**			HH	70	38	430

***Table S1**: The 12 datasets resulting from the combination of software packages, codings and historical linguistic classifications, with the number of structural features, language families and languages processed. For MrBayes all outgroups have been combined. For details see main text and [6].*

Case	Diag	Measure	Observed	Simulated			*p*-value
				Mean	SD	SDs from mean	
**MBE**	9.27	**NN**	2.222	3.304	0.039	27.957	**$< 10^{-4}$**
		**Mean**	2.749	3.664	0.032	28.940	**$< 10^{-4}$**
**MBW**	9.27	**NN**	2.570	3.333	0.045	17.073	**$< 10^{-4}$**
		**Mean**	3.017	3.627	0.037	16.496	**$< 10^{-4}$**
**MBH**	9.27	**NN**	2.207	3.289	0.036	30.434	**$< 10^{-4}$**
		**Mean**	2.736	3.682	0.030	31.858	**$< 10^{-4}$**
**MPE**	8.37	**NN**	2.024	2.930	0.038	23.887	**$< 10^{-4}$**
		**Mean**	2.484	3.308	0.032	26.121	**$< 10^{-4}$**
**MPW**	8.25	**NN**	2.366	2.948	0.055	10.591	**$< 10^{-4}$**
		**Mean**	2.641	3.171	0.044	12.055	**$< 10^{-4}$**
**MPH**	8.60	**NN**	2.162	3.011	0.035	24.578	**$< 10^{-4}$**
		**Mean**	2.642	3.416	0.029	26.521	**$< 10^{-4}$**
**BBE**	9.27	**NN**	1.413	3.289	0.035	53.805	**$< 10^{-4}$**
		**Mean**	2.147	3.681	0.029	52.111	**$< 10^{-4}$**
**BBW**	9.27	**NN**	1.990	3.329	0.044	30.314	**$< 10^{-4}$**
		**Mean**	2.561	3.633	0.036	29.395	**$< 10^{-4}$**
**BBH**	9.27	**NN**	1.494	3.288	0.035	51.428	**$< 10^{-4}$**
		**Mean**	2.167	3.681	0.029	51.497	**$< 10^{-4}$**
**BPE**	8.37	**NN**	1.308	2.949	0.042	39.412	**$< 10^{-4}$**
		**Mean**	1.823	3.285	0.035	42.051	**$< 10^{-4}$**
**BPW**	8.37	**NN**	1.757	2.961	0.044	27.070	**$< 10^{-4}$**
		**Mean**	2.184	3.271	0.037	29.212	**$< 10^{-4}$**
**BPH**	8.37	**NN**	1.378	2.916	0.035	44.006	**$< 10^{-4}$**
		**Mean**	1.879	3.319	0.029	48.818	**$< 10^{-4}$**

Table S2*: Comparison between the observed stability distances between the actual language families and 10,000 simulations of random language families.* ***Diag****: the maximum possible stability distance in the stability cube with the number of dimensions equal to the number of features in this case.* ***Measure****: the summary of the stability distances matrix between language families (****NN*** *is the average distance to the nearest neighbor;* ***Mean*** *is the mean stability distance).* ***Observed****: the actual value of the measure for the real language families.* ***Simulated****: 10,000 simulations of random language families in the current stability cube resulting in 10,000 simulated stability distance matrices; for each such simulated matrix the measures as defined above have been computed and the distribution of these 10,000 simulated measures was summarized using their* ***Mean*** *and standard deviation (****SD****);* ***SDs from mean*** *represents how far is the observed measure from the mean of the simulated measures in terms of the simulated SDs.* ***P-value****: the empirical probability that the observed measure is outside the simulated distribution.*

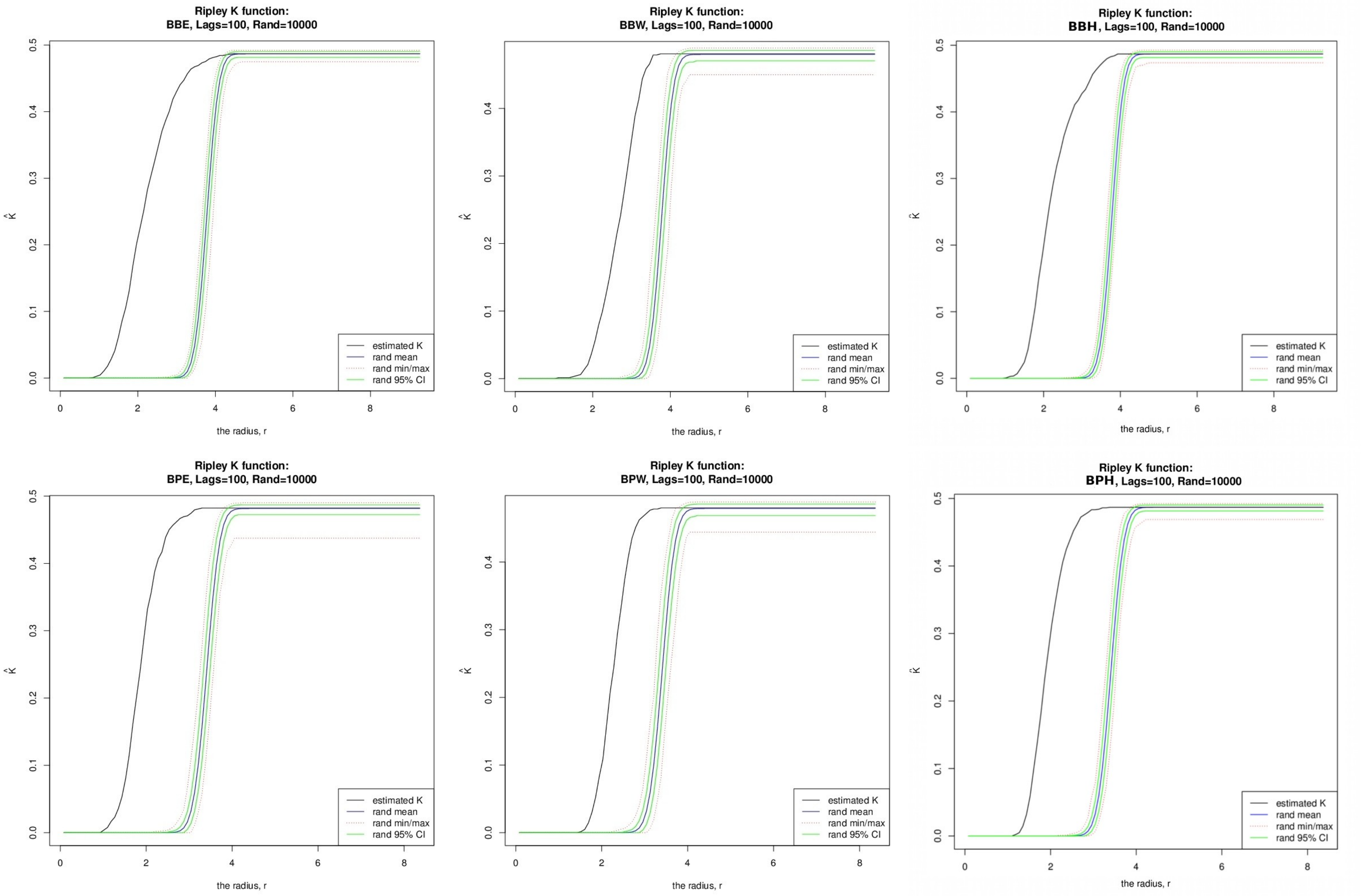

***Figure S1**: The observed Ripley's **K** function (computed at 100 lags) for the 4 datasets using BayesLang versus 10,000 simulated Poisson processes. It can be seen that the actually observed values (black) are well outside the 95% confidence interval around the mean randomizations (and, in fact, outside their whole range) for all meaningful values of the radius r.*

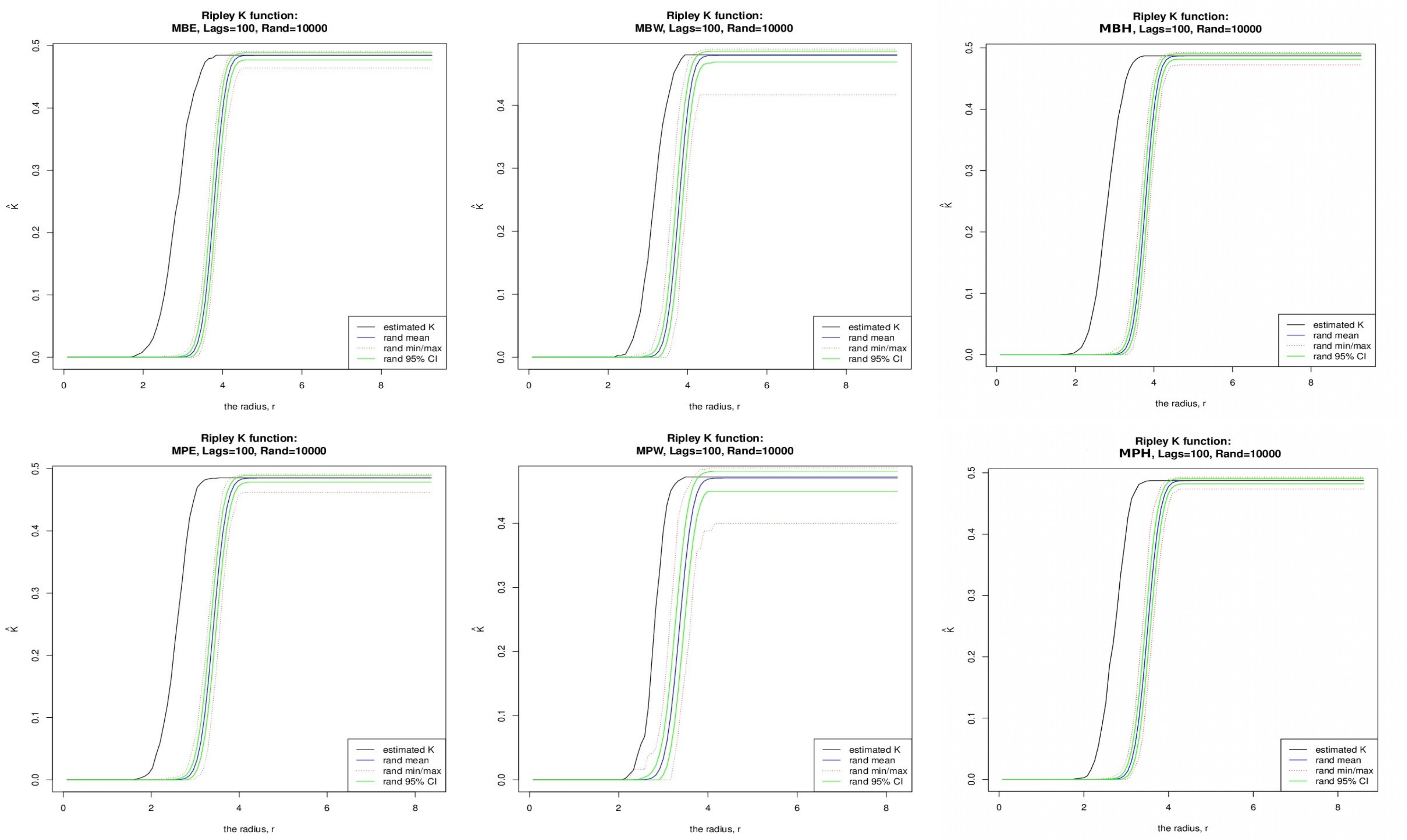

***Figure S2**: The observed Ripley's **K** function (computed at 100 lags) for the 4 datasets using MrBayes versus 10,000 simulated Poisson processes. It can be seen that the actually observed values (black) are well outside the 95% confidence interval around the mean randomizations (and, in fact, outside their whole range) for all meaningful values of the radius r.*

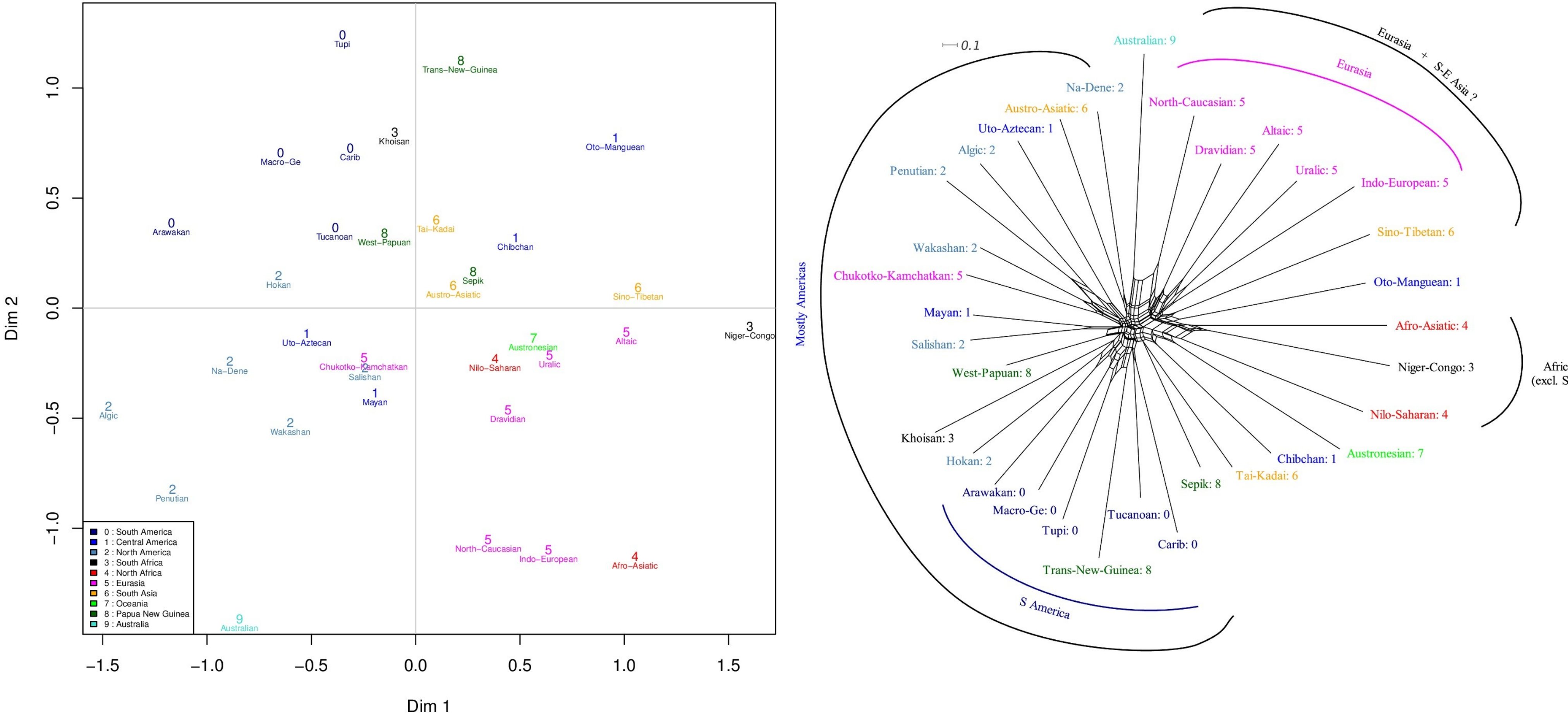

***Figure S3**: MDS (left) and annotated Network (right) representations of the stability distances between language families for dataset **MBE**. Please note that for the MDS plot only the first two dimensions are shown and the scales and directionality of the axes are arbitrary.*

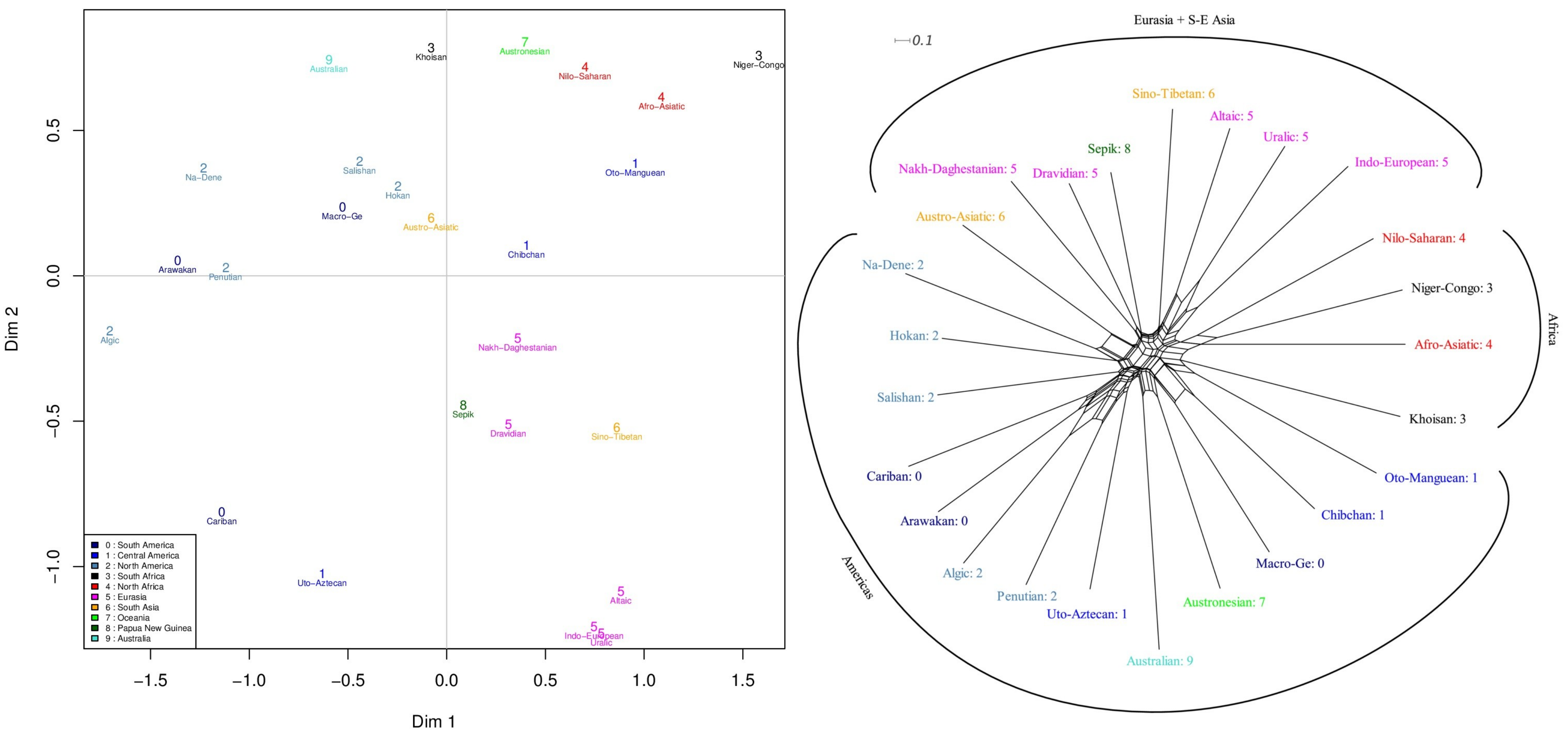

***Figure S4**: MDS (left) and annotated Network (right) representations of the stability distances between language families for dataset **MBW**. Please note that for the MDS plot only the first two dimensions are shown and the scales and directionality of the axes are arbitrary.*

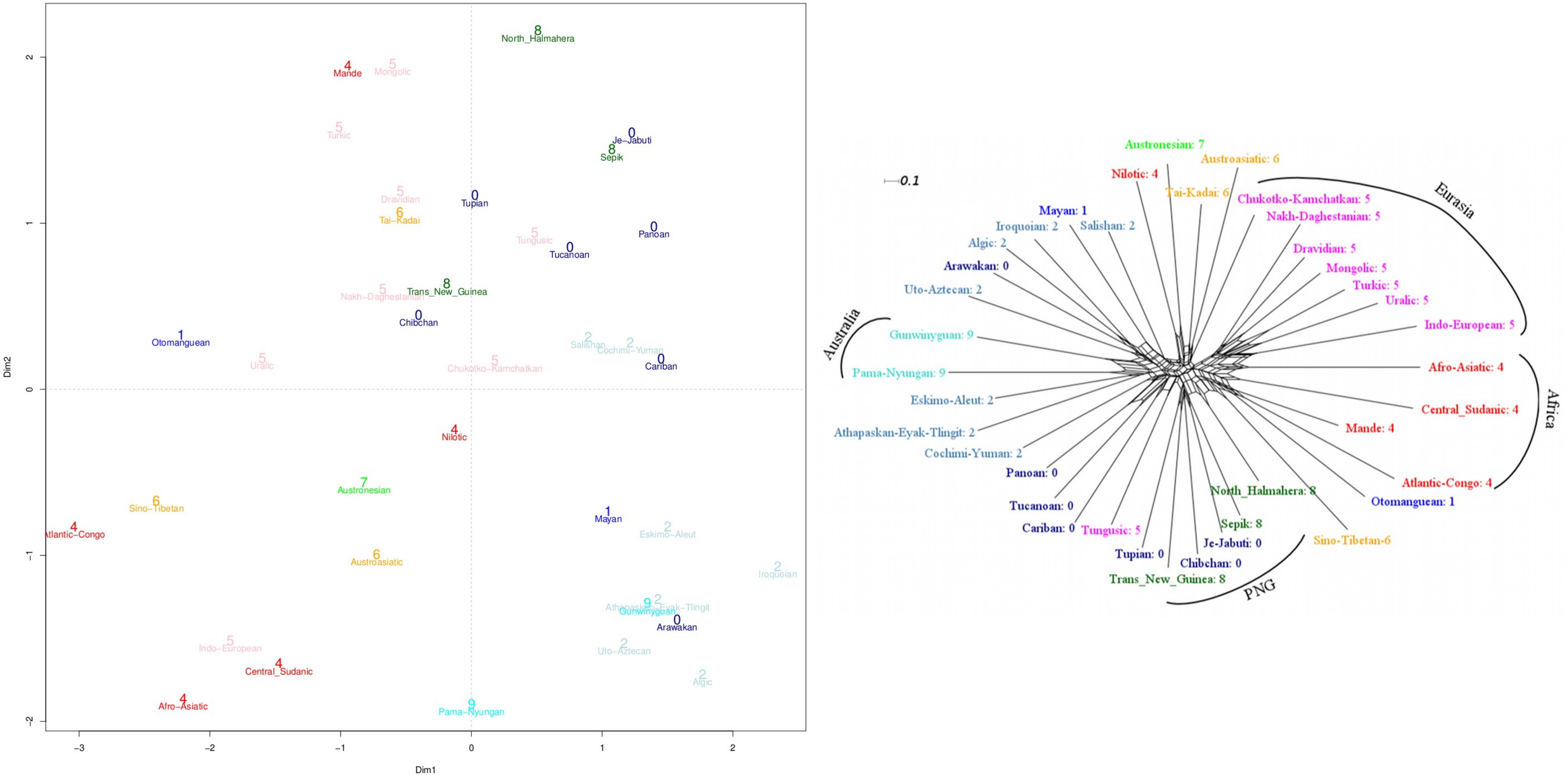

***Figure S5**: MDS (left) and annotated Network (right) representations of the stability distances between language families for dataset **MBH**. Please note that for the MDS plot only the first two dimensions are shown and the scales and directionality of the axes are arbitrary.*

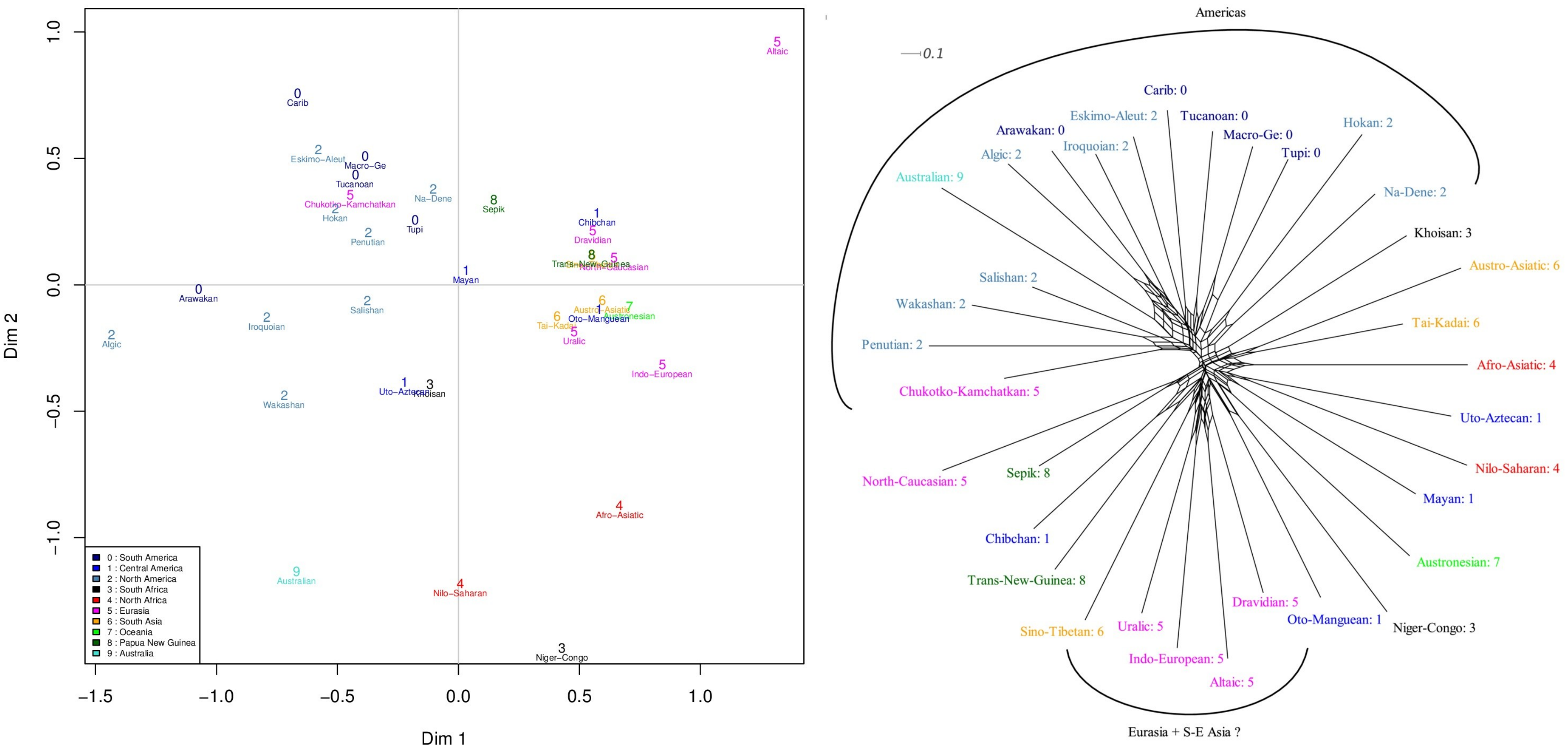

Figure S6: *MDS (left) and annotated Network (right) representations of the stability distances between language families for dataset* ***MPE****. Please note that for the MDS plot only the first two dimensions are shown and the scales and directionality of the axes are arbitrary.*

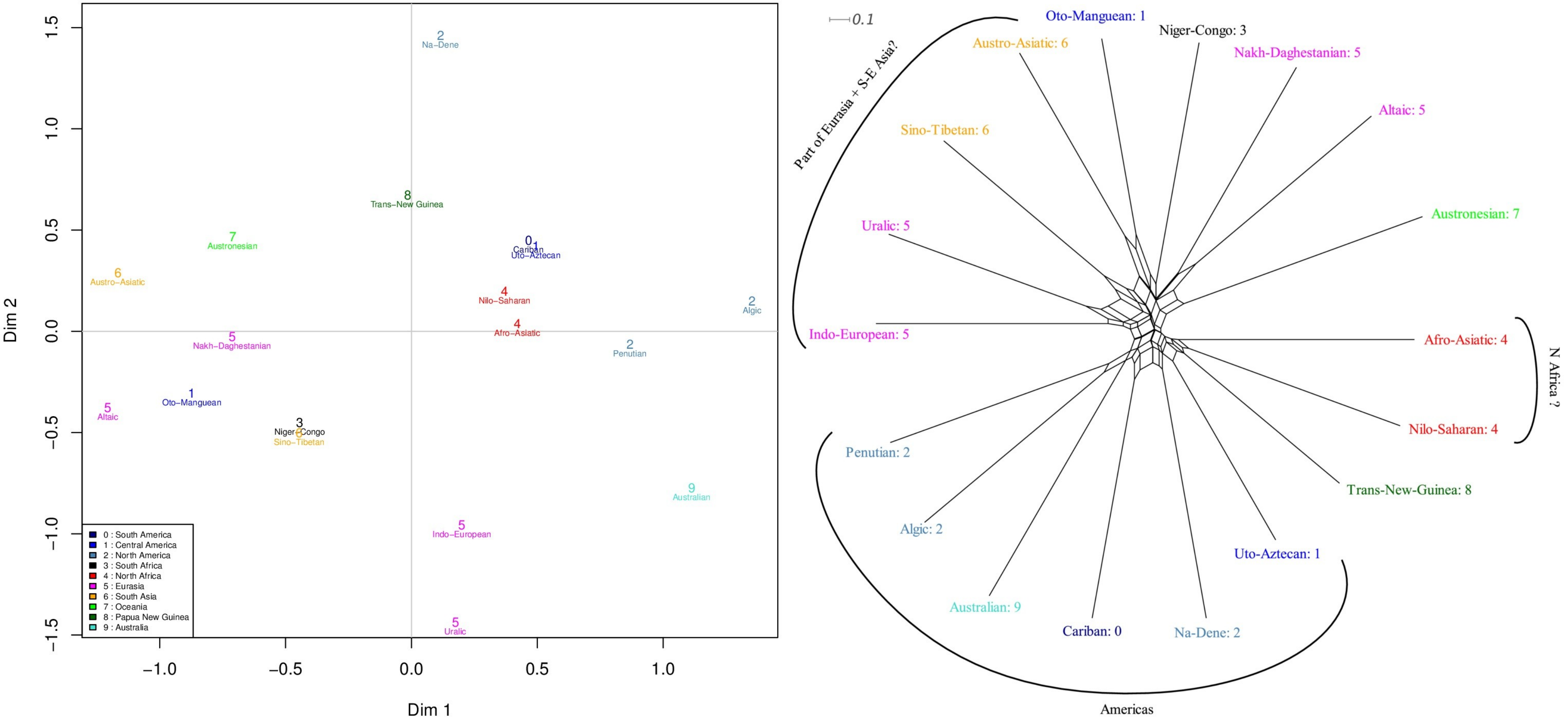

***Figure S7**: MDS (left) and annotated Network (right) representations of the stability distances between language families for dataset **MPW**. Please note that for the MDS plot only the first two dimensions are shown and the scales and directionality of the axes are arbitrary.*

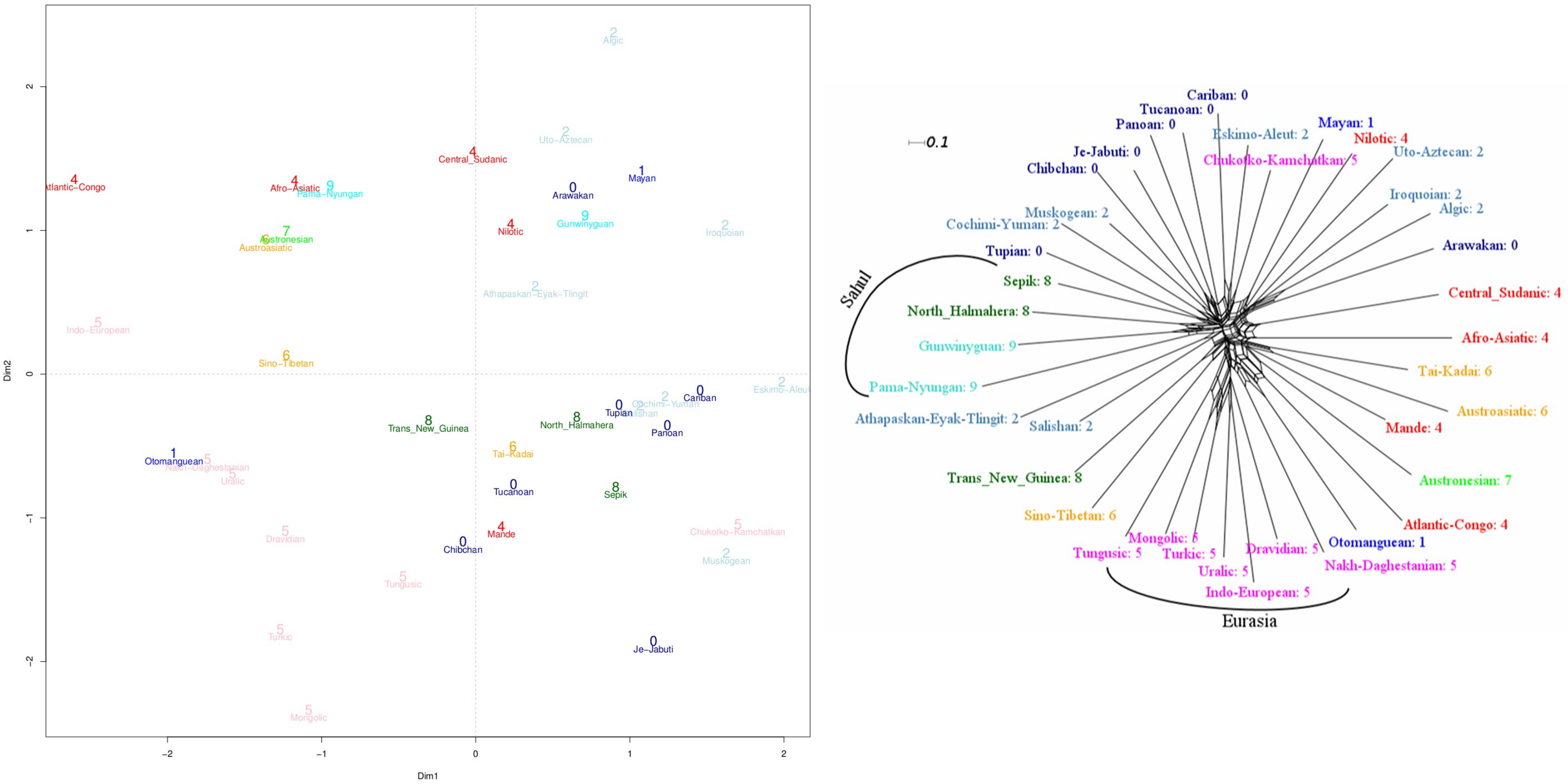

Figure S8: *MDS (left) and annotated Network (right) representations of the stability distances between language families for dataset* ***MPH****. Please note that for the MDS plot only the first two dimensions are shown and the scales and directionality of the axes are arbitrary.*

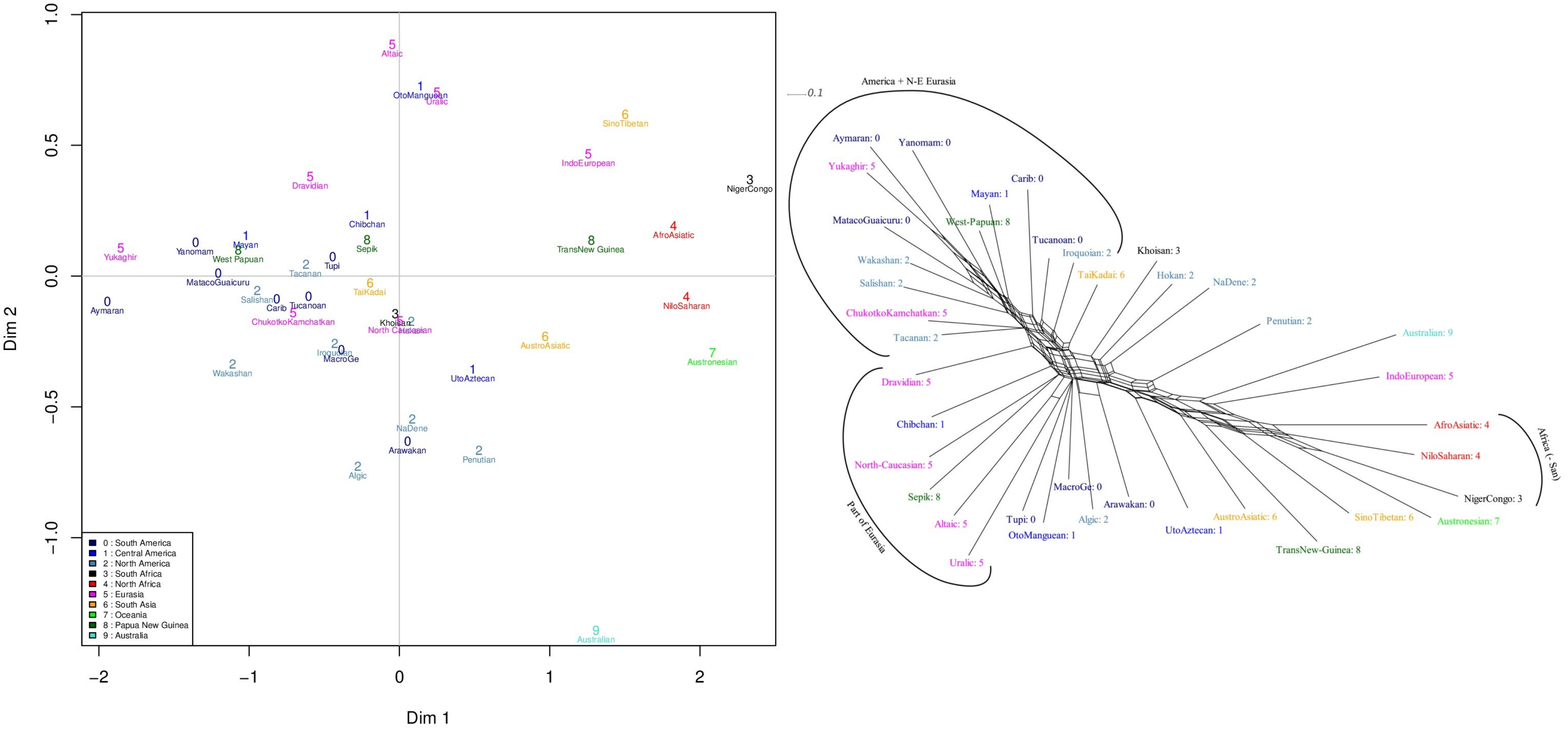

Figure S9: *MDS (left) and annotated Network (right) representations of the stability distances between language families for dataset* ***BBE****. Please note that for the MDS plot only the first two dimensions are shown and the scales and directionality of the axes are arbitrary.*

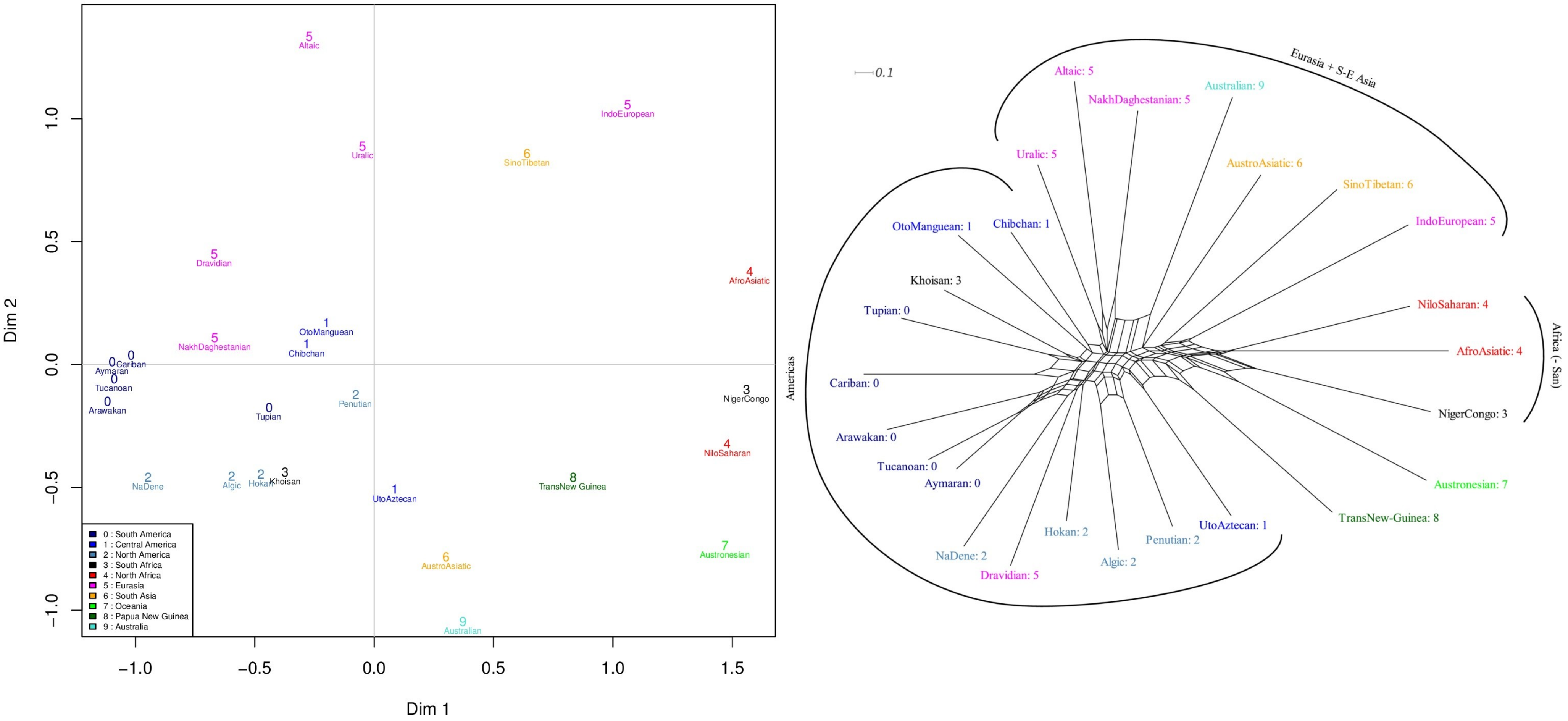

***Figure S10**: MDS (left) and annotated Network (right) representations of the stability distances between language families for dataset **BBW**. Please note that for the MDS plot only the first two dimensions are shown and the scales and directionality of the axes are arbitrary.*

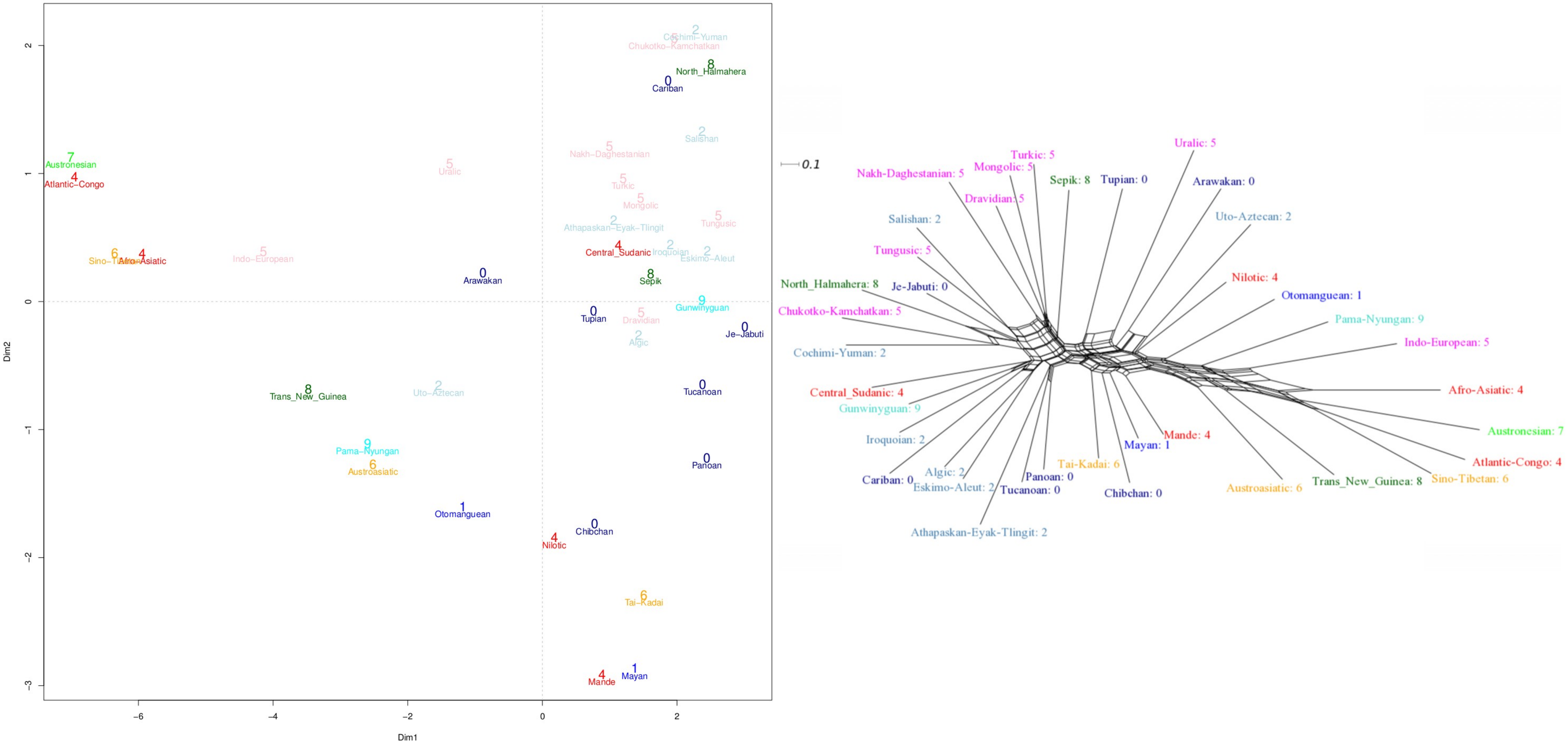

Figure S11: *MDS (left) and annotated Network (right) representations of the stability distances between language families for dataset* ***BBH****. Please note that for the MDS plot only the first two dimensions are shown and the scales and directionality of the axes are arbitrary.*

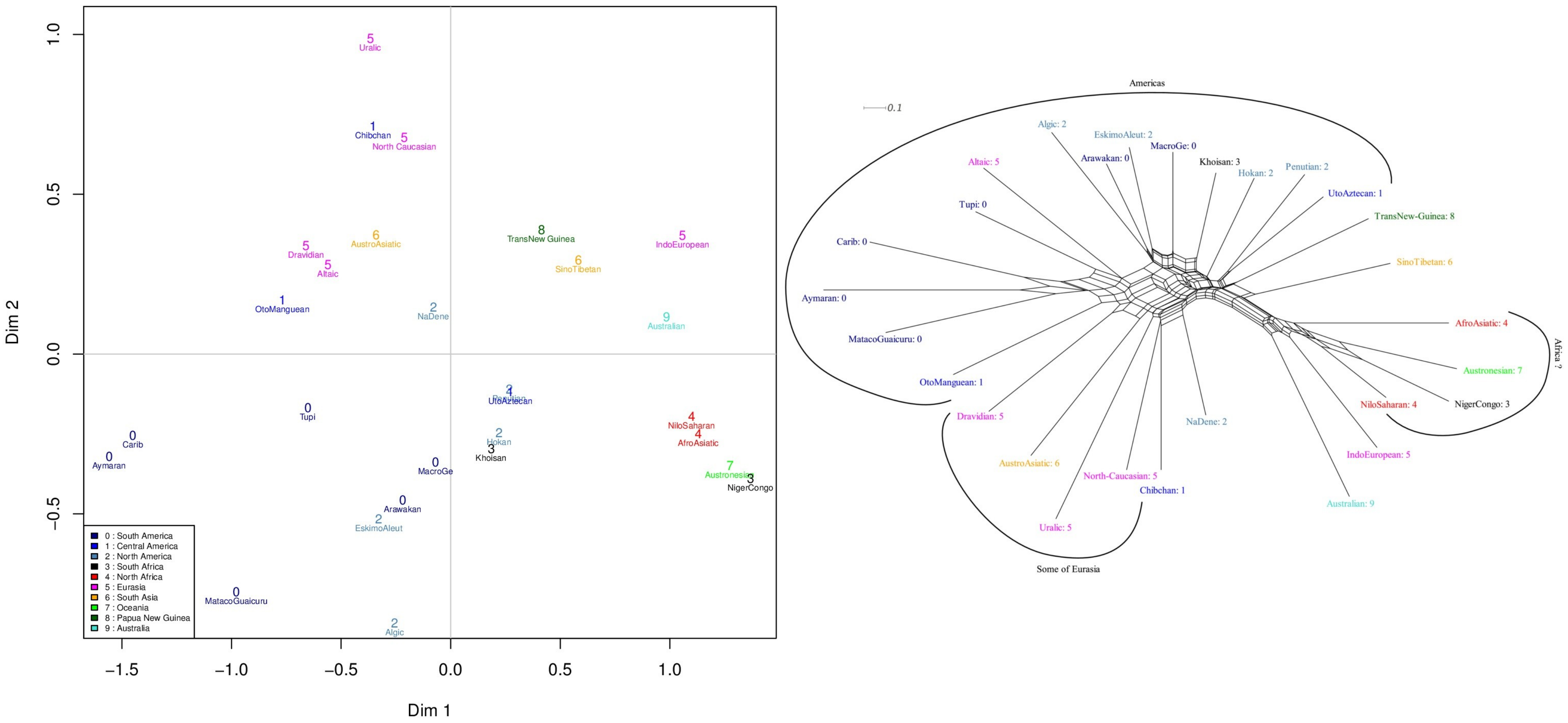

Figure S12: *MDS (left) and annotated Network (right) representations of the stability distances between language families for dataset* ***BPE****. Please note that for the MDS plot only the first two dimensions are shown and the scales and directionality of the axes are arbitrary.*

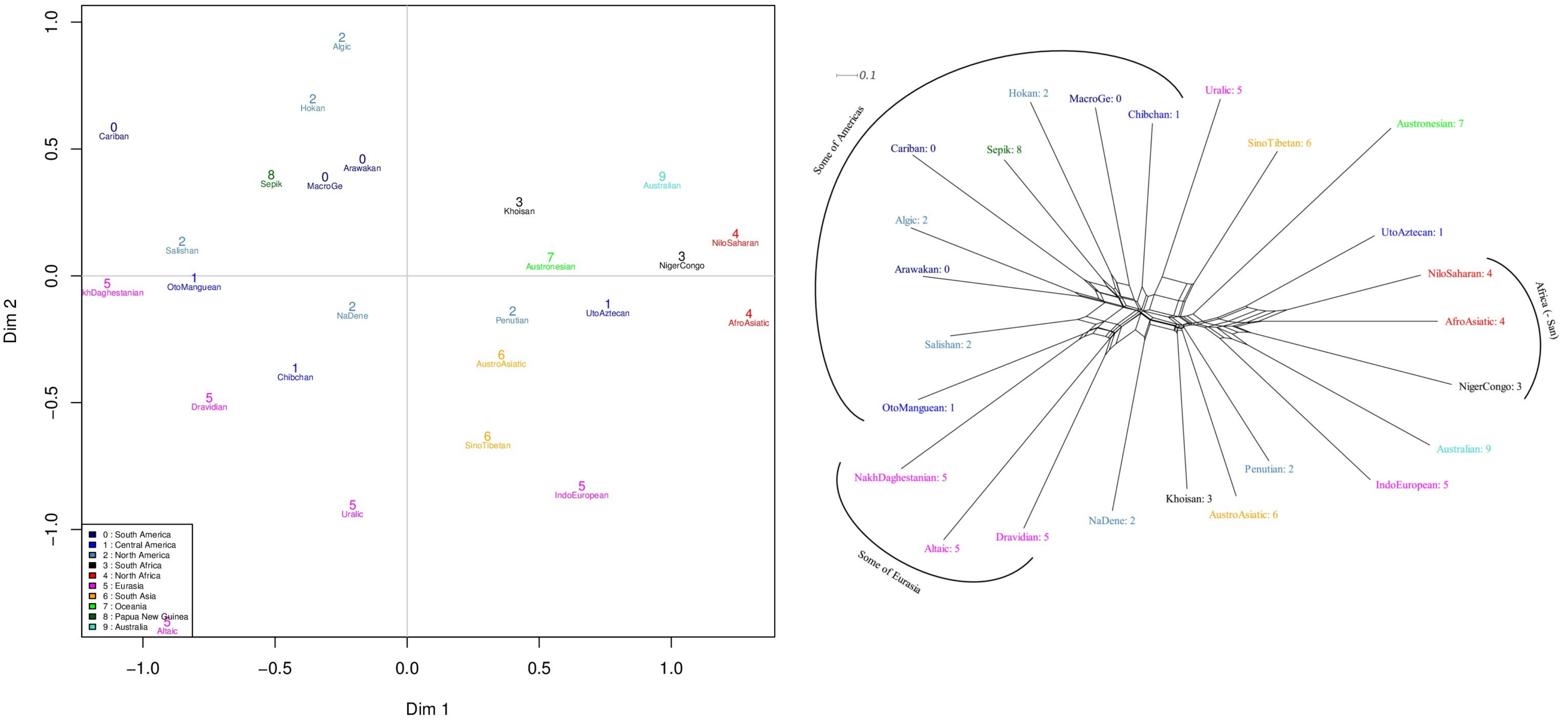

***Figure S13**: MDS (left) and annotated Network (right) representations of the stability distances between language families for dataset **BPW**. Please note that for the MDS plot only the first two dimensions are shown and the scales and directionality of the axes are arbitrary.*

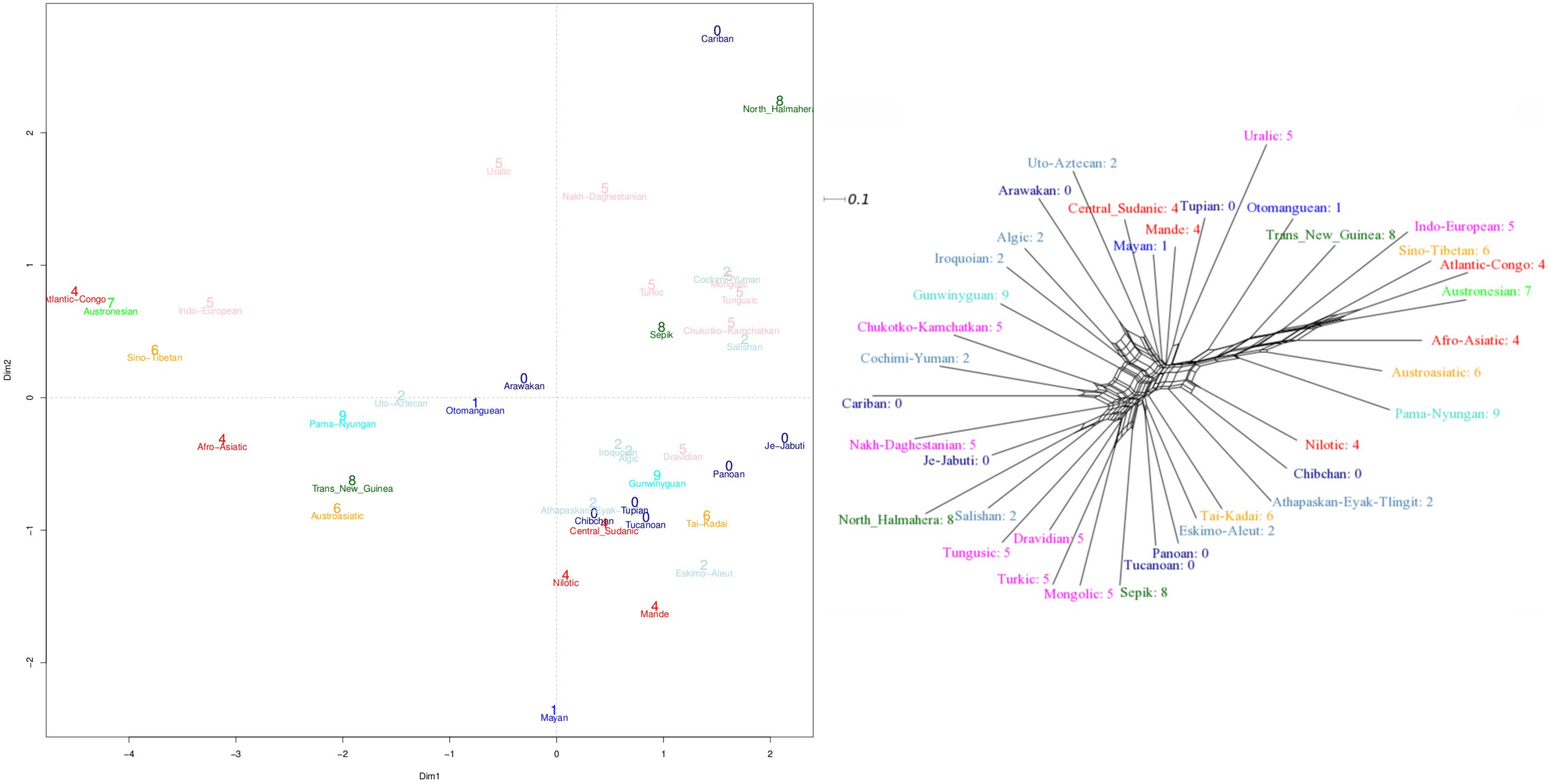

Figure S14: *MDS (left) and annotated Network (right) representations of the stability distances between language families for dataset* ***BPH****. Please note that for the MDS plot only the first two dimensions are shown and the scales and directionality of the axes are arbitrary.*

ID	WALS Feature Name	Short	Binary	Poly	Bin
1	Consonant Inventories	**Cons**	**Cons1** (Average and small vs large), **Cons2** (Small vs average and large)	**68**	**70, 75**
2	Vowel Quality Inventories	**Vowel**	**Vowel1** (Average and small vs large), **Vowel2** (Small vs average and large)	**41**	**45, 16**
3	Consonant-Vowel Ratio	**CVRatio**	**CVRatio1** (Average and small vs large), **CVRatio2** (Small vs average and large)	**67**	**61, 67**
4	Voicing in Plosives and Fricatives	**VoicPF**	**VoicPF1** (None vs at least in one), **VoicPF2** (Both vs at most in one)	**55**	**52, 28**
6	Uvular Consonants	**UvulC**	**UvulC** (None vs at least in one)	**9**	**23**
7	Glottalized Consonants	**GlotC**	**GlotC** (None vs at least in one)	**19**	**21**
8	Lateral Consonants	**LatC**	**LatC** (None vs at least in one)	**44**	**27**
9	The Velar Nasal	**VelarN**	**VelarN** (No vs yes)	**38**	**43**
10	Vowel Nasalization	**VowelN**	**VowelN** (No vs yes)	**4**	**33**
11	Front Rounded Vowels	**FrRoundV**	**FrRoundV** (None vs at least in one)	**2**	**8**
12	Syllable Structure	**SylStr**	**SylStr1** (Simple and moderate vs complex), **SylStr2** (Simple vs moderate and complex)	**45**	**76, 10**
13	Tone	**Tone**	**Tone1** (No tone vs any form of tone), **Tone2** (Absent and simple vs complex)	**15**	**37, 9**
14	Fixed Stress Locations	**FixStress**	**FixStress** (No vs yes)		**80**
18	Absence of Common Consonants	**AbsComC**	**AbsComC** (No vs any)	**1**	**5**
19	Presence of Uncommon Consonants	**PresUnC**	**PresUnC** (None vs at least one)		**31**
21	Exponence of Selected Inflectional Formatives	**ESIflF**	**ESIflF** (No case vs case)		**58**
22	Inflectional Synthesis of the Verb	**IflSVerb**	**IflSVerb** (0-5 vs 6-13 categories per word)		**68**
23	Locus of Marking in the Clause	**LmarkC**	**LmarkC** (No marking vs marking)	**54**	**41**
24	Locus of Marking in Possessive Noun Phrases	**LmarkPNP**	**LmarkPNP** (No marking vs marking)	**48**	**22**
27	Reduplication	**Redup**	**Redup** (No vs any)	**23**	
28	Case Syncretism	**CaseS**	**CaseS** (No synchretism vs any)		**48**
29	Syncretism in Verbal Person/Number Marking	**SynVPNM**	**SynVPNM** (No synchretism vs any)		**57**
30	Number of Genders	**NoGen**	**NoGen** (None vs any)	**39**	**44**
31	Sex-based and Non-sex-based Gender Systems	**SexGen**	**SexGen** (Sex-based vs non-sex-based)		**30**
32	Systems of Gender Assignment	**SgenAss**	**SgenAss** (Semantic vs semantic and formal)		**35**
34	Occurrence of Nominal Plurality	**OccNPlu**	**OccNPlu** (None vs any)		**14**
36	The Associative Plural	**AssocPlu**	**AssocPlu** (Same as addtive vs unique)		**64**
37	Definite Articles	**DefArt**	**DefArt** (No vs any)	**62**	**82**

ID	WALS Feature Name	Short	Binary	Poly	Bin
38	Indefinite Articles	**IndefArt**	**IndefArt** (No vs an)	**60**	**83**
41	Distance Contrasts in Demonstratives	**DistCDem**	**DistCDem** (Two way vs more than two)	**49**	**84**
42	Pronominal and Adnominal Demonstratives	**PadDem**	**PadDem** (Identical vs different)	**28**	
43	Third Person Pronouns and Demonstratives	**P3PrDem**	**P3PrDem** (Related vs unrelated)	**63**	**77**
44	Gender Distinctions in Independent Personal Pronouns	**GenDIPersP**	**GenDIPersP** (No distinction vs any)	**35**	**47**
45	Politeness Distinctions in Pronouns	**PolitDPron**	**PolitDPron** (No distinction vs any)	**42**	**65**
47	Intensifiers and Reflexive Pronouns	**IntReflPron**	**IntReflPron** (Identical vs differentiated)		**60**
48	Person Marking on Adpositions	**PersMAdpos**		**50**	**53**
49	Number of Cases	**Ncases**	**Ncases** (No case marking vs any)	**65**	
50	Asymmetrical Case-Marking	**AsymCaseM**	**AsymCaseM** (Symmetrical vs asymmetrical)	**61**	**71**
52	Comitatives and Instrumentals	**ComInstr**	**ComInstr** (Identity vs differentiation)		**36**
53	Ordinal Numerals	**OrdNum**	**OrdNum** (None vs any)	**66**	**25**
54	Distributive Numerals	**DistNum**	**DistNum** (None vs any)		**39**
55	Numeral Classifiers	**NumClas**	**NumClas** (Absent vs present)	**13**	**42**
56	Conjunctions and Universal Quantifiers	**ConjUQu**	**ConjUQu** (Different vs similar)		**54**
57	Position of Pronominal Possessive Affixes	**PosProPAff**	**PosProPAff** (None vs any)	**34**	**59**
58	Obligatory Possessive Inflection	**OlbPosInfl**	**OlbPosInfl** (Absent vs present)	**5**	**29**
59	Possessive Classification	**PosClas**	**PosClas** (None vs any)	**29**	**56**
63	Noun Phrase Conjunction	**NounPConj**	**NounPConj** (Different vs identical)		**63**
64	Nominal and Verbal Conjunction	**NomVConj**	**NomVConj** (Identity vs differentiation)	**36**	**62**
65	Perfective/Imperfective Aspect	**PerfImpAsp**	**PerfImpAsp** (No vs yes)	**20**	**66**
66	The Past Tense	**PastTense**	**PastTense** (No past tense vs any)	**32**	**50**
67	The Future Tense	**FutTense**	**FutTense** (No vs yes)	**22**	**69**
68	The Perfect	**Perfect**	**Perfect** (No perfect vs any)	**43**	**73**
70	The Morphological Imperative	**MorphImp**	**MorphImp** (No second-person imperative vs any)	**51**	**26**
73	The Optative	**Optative**	**Optative** (Absent vs present)	**3**	**17**
76	Overlap between Situational and Epistemic Modal Marking	**OvSitEpi**	**OvSitEpi** (No overlap vs overlap)	**47**	**78**

ID	WALS Feature Name	Short	Binary	Poly	Bin
77	Semantic Distinctions of Evidentiality	**SemDistEv**	**SemDistEv** (No grammatical evidential vs any)	**46**	**72**
79	Suppletion According to Tense and Aspect	**SuppTAsp**	**SuppTAsp** (None vs any)	**25**	**55**
80	Verbal Number and Suppletion	**VnumSupp**	**VnumSupp** (None vs any)	**33**	**32**
82	Order of Subject and Verb	**SV**	**SV1** (No dominant order vs any dominant order), **SV2** (SV vs VS)	**14**	**12, 15**
83	Order of Object and Verb	**OV**	**OV1** (No dominant order vs any dominant order), **OV2** (OV vs VO)	**11**	**6, 19**
85	Order of Adposition and Noun Phrase	**AdposNP**	**AdposNP** (Postpositions vs prepositions)	**16**	**13**
86	Order of Genitive and Noun	**GenN**	**GenN1** (No dominant order vs any dominant order), **GenN2** (GN vs NG)	**6**	**3, 11**
87	Order of Adjective and Noun	**AdjN**	**AdjN1** (No dominant order vs any dominant order), **AdjN2** (AdjN vs Nadj)	**31**	**4, 38**
89	Order of Numeral and Noun	**NumN**	**NumN1** (No dominant order vs any dominant order), **NumN2** (NumN vs NNum)	**12**	**1, 24**
91	Order of Degree Word and Adjective	**DegWAdj**	**DegWAdj1** (No dominant order vs any dominant order), **DegWAdj2** (DegAdj vs AdjDeg)	**30**	**18, 40**
92	Position of Polar Question Particles	**PolQPart**	**PolQPart** (No question particle vs any)	**64**	**85**
93	Position of Interrogative Phrases in Content Questions	**IntPhCQ**	**IntPhCQ1** (No dominant order vs any dominant order), **IntPhCQ2** (Initial vs non-initial)	**27**	**2, 51**
95	Relationship between the Order of Object and Verb and the Order of Adposition and Noun Phrase	**OVAdpNP**	**OVAdpNP** (Head first vs head second)	**37**	**7**
96	Relationship between the Order of Object and Verb and the Order of Relative Clause and Noun	**OVRelN**	**OVRelN** (Head first vs head second)	**52**	**20**
97	Relationship between the Order of Object and Verb and the Order of Adjective and Noun	**OVAdjN**	**OVAdjN** (Head first vs head second)	**58**	**49**
102	Verbal Person Marking	**VpersM**	**VpersM** (No person marking vs any)	**53**	**34**
104	Order of Person Markers on the Verb	**PersMV**	**PersMV** (A and P do not or do not both occur on the verb vs any)	**59**	**74**
107	Passive Constructions	**PassiveC**	**PassiveC** (Absent vs present)	**26**	**79**
108	Antipassive Constructions	**AntipassiveC**	**AntipassiveC** (No antipassive vs any)	**18**	**46**
109	Applicative Constructions	**ApplicativeC**	**ApplicativeC** (No applicative vs any)	**57**	
113	Symmetric and Asymmetric Standard Negation	**SymAsymStNeg**	**SymAsymStNeg1** (Symmetric vs asymmetric and both), **SymAsymStNeg2** (Symmetric and both vs asymmetric)	**56**	**81, 86**
118	Predicative Adjectives	**PredAdj**	**PredAdj1** (Verbal encoding vs any), **PredAdj2** (Nonverbal encoding vs any)	**24**	
119	Nominal and Locational Predication	**NomLocPred**	**NomLocPred** (Different vs identical)	**8**	
120	Zero Copula for Predicate Nominals	**ZeroCopPredNom**	**ZeroCopPredNom** (Possible vs impossible)	**21**	

ID	WALS Feature Name	Short	Binary	Poly	Bin
126	`When' Clauses	**WhenC**	**WhenC1** (Balanced vs any), **WhenC2** (Deranked vs any)	**40**	
129	Hand and Arm	**HandArm**	**HandArm** (Identical vs different)	**17**	
136	M-T Pronouns	**MTPron**	**MTPron** (No M-T pronouns vs any)	**10**	
137	N-M Pronouns	**NMPron**	**NMPron** (No N-M pronouns vs any)	**7**	
**The features listed from the most stable to the most unstable**					
The **polymorphic** features: most stable → most unstable		AbsComC, FrRoundV, Optative, VowelN, OlbPosInfl, GenN, NMPron, NomLocPred, UvulC, MTPron, OV, NumN, NumClas, SV, Tone, AdposNP, HandArm, AntipassiveC, GlotC, PerfImpAsp, ZeroCopPredNom, FutTense, Redup, PredAdj, SuppTAsp, PassiveC, IntPhCQ, PadDem, PosClas, DegWAdj, AdjN, PastTense, VnumSupp, PosProPAff, GenDIPersP, NomVConj, OVAdpNP, VelarN, NoGen, WhenC, Vowel, PolitDPron, Perfect, LatC, SylStr, SemDistEv, OvSitEpi, LmarkPNP, DistCDem, PersMAdpos, MorphImp, OVRelN, VpersM, LmarkC, VoicPF, SymAsymStNeg, ApplicativeC, OVAdjN, PersMV, IndefArt, AsymCaseM, DefArt, P3PrDem, PolQPart, Ncases, OrdNum, CVRatio, Cons			
The **binary** features: most stable → most unstable		NumN1, IntPhCQ1, GenN1, AdjN1, AbsComC, OV1, OVAdpNP, FrRoundV, Tone2, SylStr2, GenN2, SV1, AdposNP, OccNPlu, SV2, Vowel2, Optative, DegWAdj1, OV2, OVRelN, GlotC, LmarkPNP, UvulC, NumN2, OrdNum, MorphImp, LatC, VoicPF2, OlbPosInfl, SexGen, PresUnC, VnumSupp, VowelN, VpersM, SgenAss, ComInstr, Tone1, AdjN2, DistNum, DegWAdj2, LmarkC, NumClas, VelarN, NoGen, Vowel1, AntipassiveC, GenDIPersP, CaseS, OVAdjN, PastTense, IntPhCQ2, VoicPF1, PersMAdpos, ConjUQu, SuppTAsp, PosClas, SynVPNM, ESIflF, PosProPAff, IntReflPron, CVRatio1, NomVConj, NounPConj, AssocPlu, PolitDPron, PerfImpAsp, CVRatio2, IflSVerb, FutTense, Cons1, AsymCaseM, SemDistEv, Perfect, PersMV, Cons2, SylStr1, P3PrDem, OvSitEpi, PassiveC, FixStress, SymAsymStNeg1, DefArt, IndefArt, DistCDem, PolQPart, SymAsymStNeg2			

Table S3:** The typological features used here, with their full WALS unique ID and name, the short name used in this paper and the binary aspects (if any).* ***Poly *and* ***Bin*** *show the consensus between the 12 datasets:* ***Poly*** *gives the stability rank of the polymorphic feature, if any (between 1 = most stable to 68 = most unstable), and* ***Bin*** *gives the stability rank of the binary aspect(s) separated by comma (from 1 = most stable to 86 = most unstable).* ***Poly*** *and* ***Bin*** *cells are empty for those features not included in the stability estimation (as polymorphic or binary) for various reasons such as data coverage and coding meaningfulness. The last two rows give the lists of polymorphic and binary features ordered from the most stable to most unstable (for easiness of comparison). See [9] and especially Tables* ***S7****,* ***S4*** *and* ***S10*** *in there for more details and explanations.*

Family	Case	Structure	Size
Afro-Asiatic	BBE	(((arz,heb),(amh,tig)),((((anc,sur),hau,kna),ngi),ker,(mrt,ttr)),(bej,irk,(hae,som)),tzm)	17
	BBH	(((tzm,shi),thv),(((gde,ttr,mrt),bdm),(lln,ker),(hau,((kna,pip),(anc,sur)),(ngi,mkf))),(awn,(hae,aar,som),bej,(bds,irk)),cop,((aij,((ary,arz,apc,afb),heb)),(tig,amh)))	32
	BBW	((amh,arz,heb,tig),(anc,hau,kna,sur,ngi),bej,tzm,irk,ker,(mrt,ttr),(hae,som))	17
	BPE	(((arz,heb),(amh,tig)),(bej,irk,(hae,som)),(tzm,shi),(hau,ker,mrt))	13
	BPH	(((tzm,shi),thv),((gde,ttr,mrt),(lln,ker),(hau,((kna,pip),(anc,sur)),(ngi,mkf))),((hae,som),bej,(bds,irk)),cop,((((ary,arz,apc,afb,mlt),heb)),(tig,amh)))	29
	BPW	((amh,arz,heb,tig),bej,tzm,mdx,(hau,kna,sur,ngi),irk,ker,(mrt,ttr),(hae,som))	17
	MBE	(aiw,((arz,heb),(amh,tig)),((((anc,sur),hau,kna),ngi),ker,(mrt,ttr)),(bej,irk,(hae,som)),tzm)	18
	MBH	(((tzm,shi),thv),(((gde,ttr,mrt),bdm),(lln,ker),(hau,((kna,pip),(anc,sur)),(ngi,mkf))),(awn,(hae,aar,som),bej,(bds,irk)),cop,((aij,((ary,arz,apc,afb),heb)),(tig,amh)))	32
	MBW	((amh,arz,heb,tig),(anc,hau,kna,sur,ngi),bej,tzm,irk,ker,(mrt,ttr),(hae,som))	17
	MPE	((aiw,mdx),((arz,heb),(amh,tig)),(bej,irk,(hae,som)),tzm,(((hau,kna,sur),ngi),ker,(mrt,ttr)))	18
	MPH	(((tzm,shi),thv),((gde,ttr,mrt),(lln,ker),(hau,((kna,pip),(anc,sur)),(ngi,mkf))),((hae,som),bej,(bds,irk)),cop,((((ary,arz,afb,mlt),heb)),(tig,amh)))	28
	MPW	((amh,arz,heb,tig),bej,tzm,(hau,ngi),irk,ker,mrt,(hae,som))	13
Algic	BBE	(((crk,ojg),pqm),yur)	4
	BBH	((bla,crk,pqm,ojg),wiy,yur)	6
	BBW	((crk,ojg,pqm),yur)	4
	BPE	((crk,pqm),yur)	3
	BPH	((bla,crk,pqm,ojg),wiy,yur)	6
	BPW	((crk,pqm),wiy,yur)	4
	MBE	(((abe,pqm),(crk,ojg)),yur)	5
	MBH	((bla,crk,pqm,ojg),wiy,yur)	6
	MBW	((crk,ojg,pqm),yur)	4
	MPE	(((abe,pqm),crk),wiy,yur)	5
	MPH	((bla,crk,pqm,ojg),wiy,yur)	6
	MPW	((crk,pqm),yur)	3
Altaic	BBE	(((azb,tur),bak,chv,(tyv,sah)),(dta,khk,mjg),evn)	10
	BBW	((azb,bak,chv,tur,tyv,sah),(dta,khk,mjg),evn)	10
	BPE	(bak,chv,tur,(tyv,sah),evn,khk)	7
	BPW	((azb,bak,chv,tur,tyv,sah),(dta,khk,mjg),(evn,evn))	11
	MBE	(((alt,tyv,sah),(azb,tur),bak,chv),(dta,khk,mjg),evn)	11
	MBW	((azb,bak,chv,tur,tyv,sah),(dta,khk,mjg),evn)	10

Family	Case	Structure	Size
	MPE	(((alt,tyv,sah),(azb,tur),bak,chv),(dta,khk,mjg),(eve,eve))	12
	MPW	((chv,tur),evn,khk)	4
Arawakan	BBE	(((apu,cni),(guc,rgr)))	4
	BBH	((plu,((guc,arw,cab),(rgr,tae,bae,gae,ycn)),(ign,cni,apu),ame))	13
	BBW	(apu,cni,guc,rgr)	4
	BPE	(apu,cni)	2
	BPH	((plu,((guc,arw,cab),(rgr,gae)),(ign,cni,apu),ame))	10
	BPW	(apu,cni,guc,rgr)	4
	MBE	((((aca,rgr),guc),(apu,cni)))	5
	MBH	((plu,((guc,arw,cab),(rgr,tae,bae,gae,ycn)),(ign,cni,apu),ame))	13
	MBW	(apu,cni,guc,rgr)	4
	MPE	((((aca,rgr),guc),(apu,cni)))	5
	MPH	((plu,((guc,arw,cab),(rgr,gae)),(ign,cni,apu),ame))	10
Athapaskan-Eyak-Tlingit	BBH	(nav,hup,(chp,scs),tli)	5
	BPH	(nav,hup,(chp,scs),tli)	5
	MBH	(nav,hup,(chp,scs),tli)	5
	MPH	(nav,hup,(chp,scs),tli)	5
Atlantic-Congo	BBH	(((dyo,(snf,ndv),(wol,fuv)),(kqs,tem)),((mcu,(nhu,((swh,kng,cgg,lue,nya,ndo,(zul,sna,sot),ewo),(bav,agq))),(pym,bom),(((ann,efi),(gkn,ogo))),yor,enn,ibo,amo,gbr),(gry,klu),(ewe,(adj,gaa,(lef,(nko,aka)))),((((dow,mzm),mdd),(gbp,mfc,zne)),(kfz,(dga,dag),spp))))	50
	BPH	(((dyo,(snf,ndv),(wol,fuv)),(kqs,tem)),(((nhu,((swh,kng,((cgg,lug),lue,ndo,(zul,sna,sot)),ewo),bav)),(pym,bom),(ann,ogo),yor,enn,ibo,amo,(gbr,nup)),(gry,klu),(ewe,(gaa,(lef,aka))),((((dow,mzm),mdd),(liy,gbp,mfc,zne)),(kfz,(dga,dag),spp))))	46
	MBH	(((dyo,(snf,ndv),(wol,fuv)),(kqs,tem)),((mcu,(nhu,((swh,kng,cgg,lue,nya,ndo,(zul,sna,sot),ewo),(bav,agq))),(pym,bom),(((ann,efi),(gkn,ogo))),yor,enn,ibo,amo,gbr),(gry,klu),(ewe,(adj,gaa,(lef,(nko,aka)))),((((dow,mzm),mdd),(gbp,mfc,zne)),(kfz,(dga,dag),spp))))	50
	MPH	(((dyo,(snf,ndv),(wol,fuv)),(kqs,tem)),(((nhu,((swh,kng,((cgg,lug),lue,ndo,(zul,sna,sot)),ewo),bav)),(pym,bom),yor,enn,ibo,amo,(gbr,nup)),gry,(ewe,(gaa,(lef,aka))),((((dow,mzm),mdd),(gbp,mfc,zne)),(kfz,(dga,dag),spp))))	42
Australian	BBE	((aer,dif,dbl,gyd,ktg,(vma,pjt),wyb,yii),gni,gbc,mph,(zmr,mpb,mwf),(mpc,nuy,wrr),tiw,ung,wmb)	21
	BBW	((aer,dif,dbl,ktg,vma,wyb,pjt,yii),gbc,gni,gyd,mpb,mpc,zmr,mph,mwf,nuy,tiw,ung,wmb,wrr)	21
	BPE	((alh,mpc,nuy,wrr),(aer,dif,dbl,gyd,(vma,pjt),wyb,yii),gni,mph,(zmr,mpb),tiw,ung,wmb)	19
	BPW	(alh,(aer,dif,duj,dbl,ktg,vma,wyb,pjt,yii),gbc,gni,gyd,mpb,mpc,zmr,mph,mwf,nig,nuy,tiw,ung,wmb,wrr)	24
	MBE	(((ard,dif),aer,dbl,gyd,ktg,(vma,pjt),wyb,yii),gni,gbc,mph,(zmr,mpb,mwf),(mpc,nuy,wrr),tiw,ung,wmb)	22
	MBW	((aer,dif,dbl,ktg,vma,wyb,pjt,yii),gbc,gni,gyd,mpb,mpc,zmr,mph,mwf,nuy,tiw,ung,wmb,wrr)	21

Family	Case	Structure	Size
	**MPE**	(((ard,dif),aer,duj,dbl,gyd,ktg,(vma,pjt),wyb,yii),(alh,mpc,nig,nuy,wrr),gni,gbc,mph,(zmr,mpb,mwf),tiw,ung,wmb)	25
	**MPW**	((aer,dif,dbl,vma,wyb,pjt,yii),gni,gyd,mpb,mpc,zmr,mph,nuy,tiw,ung,wmb,wrr)	18
**Austro-Asiatic**	**BBE**	((khm,(sed,kpm),(kha,kjg),caq,sza,vie))	8
	**BBH**	((sza,((sed,(kpm,crw),brb),khm),mnw,(caq,ncb),kha,kjg,vie),(khr,bfw))	14
	**BBW**	(kha,khm,kjg,unr,caq,(sed,kpm),sza,vie)	9
	**BPE**	((khm,(kha,kjg),sza,vie))	5
	**BPH**	(((tea,sza),((sed,(kpm,crw),brb),khm),(caq,ncb),kha,kjg,vie),bfw)	13
	**BPW**	((crw,sed,kpm),kha,khm,kjg,unr,caq,sza,vie)	10
	**MBE**	((((bdq,sed,kpm),khm),(kha,kjg),caq,sza,vie))	9
	**MBH**	((sza,((sed,(kpm,crw),brb),khm),mnw,(caq,ncb),kha,kjg,vie),(unr,(khr,bfw)))	15
	**MBW**	(kha,khm,kjg,unr,caq,(sed,kpm),sza,vie)	9
	**MPE**	((((bdq,(crw,kpm),sed),khm),(kha,kjg),caq,sza,vie))	10
	**MPH**	(((tea,sza),((sed,(kpm,crw),brb),khm),(caq,ncb),kha,kjg,vie),(unr,bfw))	14
	**MPW**	(kha,khm,kjg,unr,sza,vie)	6
**Austronesian**	**BBE**	(tay,((btx,bbc),cha,((((((dhv,iai),((fij,((((haw,mri),rap),smo)))),(gil,pon),pma),kwd,tnl),(khl,kij,tgc),yap),(irh,mky)),tet),(iba,ind),plt,pau,tgl,bhq),pwn,dru)	31
	**BBH**	(tay,((mnb,bhq),((xbr,nni,tet),(los,((((fij,((((haw,(tah,mri),rap),(fut,smo)),(ton,niu))),rtm),(dhv,iai),((gil,kos,((pon,mkj),woe))),cir,pma),(aty,erg,tnl),kwd),((tgc,(hla,ksd),nak),(khl,(xsi,pss),mva),(((tbo,gvs),sbe),(meu,kij))),yap),((irh,mky),(mhz,amk)))),cha,(plt,bdl),jav,ljp,(((ace,cja),(iba,ind)),sun),((btx,bbc),nia),pau,tgl),pwn,dru,tsu)	66
	**BBW**	(tay,(btx,bbc,iba,ind),cha,(dhv,fij,haw,iai,khl,kij,gil,kwd,tnl,mri,pma,pon,rap,smo,tgc),(irh,mky),plt,pwn,pau,dru,tgl,tet,bhq,yap)	31
	**BPE**	((btx,cha,((((((dhv,iai),((fij,((((haw,mri),rap))))),gil,pma)),(kij,tgc),yap),mky)),ind,plt,tgl,bhq),pwn)	19
	**BPH**	(tay,((mnb,bhq),((xbr,tet),(los,((((fij,((((haw,(tah,mri),rap),(fut,smo)),(ton,niu))),rtm),(dhv,iai),((gil,kos,((pon,mkj),woe))),cir,pma),(aty,erg,tnl),(kwd,aia)),(((tgc,ksd),nak),(jae,khl,mva),((tbo,gvs),(meu,kij))),yap),((irh,mky),mhz))),cha,(plt,bdl),(((ace,cja),(iba,ind)),sun),(btx,bbc),pau,(pam,(ceb,tgl),pag)),pwn,dru,tsu)	62
	**BPW**	((ace,btx,bbc,ind,sun),tay,cha,(dhv,fij,haw,iai,khl,kij,gil,kos,kwd,tnl,mri,mkj,pma,pon,rap,smo,tgc,cir),plt,pwn,pau,dru,mky,tgl,tet,bhq,yap)	34
	**MBE**	((ace,(iba,ind),(btx,bbc),cha,((((((dhv,iai),((fij,((((haw,mri),rap),smo)))),(gil,pon),pma),kwd,tnl),(khl,kij,tgc),yap),(irh,mky)),tet),plt,pau,tgl,bhq),tay,pwn,dru)	32
	**MBH**	(tay,((mnb,bhq),((xbr,nni,tet),(los,((((fij,((((haw,(tah,mri),rap),(fut,smo)),(ton,niu))),rtm),(dhv,iai),((gil,kos,((pon,mkj),woe))),cir,pma),(aty,erg,tnl),kwd),((tgc,(hla,ksd),nak),(khl,(xsi,pss),mva),(((tbo,gvs),sbe),(meu,kij))),yap),((irh,mky),(mhz,amk)))),cha,(plt,bdl),jav,ljp,(((ace,cja),(iba,ind)),sun),((btx,bbc),nia),pau,tgl),pwn,dru,tsu)	66
	**MBW**	(tay,(btx,bbc,iba,ind),cha,(dhv,fij,haw,iai,khl,kij,gil,kwd,tnl,mri,pma,pon,rap,smo,tgc),(irh,mky),plt,pwn,pau,dru,tgl,tet,bhq,yap)	31
	**MPE**	((((ace,ind),sun),(btx,bbc),cha,((((((dhv,iai),((fij,((((haw,mri),rap),smo)))),(kos,gil,(mkj,pon)),pma,cir),kwd,tnl),(khl,kij,tgc),yap),mky),tet),plt,pau,tgl,bhq),tay,pwn,dru)	34
	**MPH**	(tay,((mnb,bhq),((xbr,tet),(los,((((fij,((((haw,(tah,mri),rap),(fut,smo)),(ton,niu)))),(dhv,iai),((gil,kos,((pon,mkj),woe))),cir,pma),(aty,erg,tnl),(kwd,aia)),(((tgc,ksd),nak),(jae,khl,mva),((tbo,gvs),(meu,kij))),yap),((irh,mky),mhz))),cha,(plt,bdl),(((ace,cja),(iba,ind)),sun),(btx,bbc),pau,(pam,(ceb,tgl),pag)),pwn,dru,tsu)	61
	**MPW**	((btx,ind),cha,(dhv,fij,haw,iai,kij,gil,mri,pma,pon,rap,tgc),plt,pwn,mky,tgl,bhq,yap)	20
**Aymaran**	**BBE**	(ayc,jqr)	2

Family	Case	Structure	Size
	BBW	(ayc,jqr)	2
	BPE	(ayc,jqr)	2
Cariban	BBE	((car,mbc),hix)	3
	BPE	(car,hix)	2
	MBE	(((ake,mbc),car),hix)	4
	MPE	(((ake,mbc),car),hix)	4
	BBH	(apy,car,hix,mbc)	4
	BBW	(car,hix,mbc)	3
	BPH	(apy,car,hix,mbc)	4
	BPW	(car,hix,mbc)	3
	MBH	(apy,car,hix,mbc)	4
	MBW	(car,hix,mbc)	3
	MPH	(apy,car,hix,mbc)	4
	MPW	(car,hix,mbc)	3
Central-Sudanic	BBH	(niy,lgg,(yul,(bmi,(myb,sba))))	6
	BPH	(niy,lgg,(yul,(bmi,(myb,sba))))	6
	MBH	(niy,lgg,(yul,(bmi,(myb,sba))))	6
	MPH	(niy,lgg,(yul,(bmi,(myb,sba))))	6
Chibchan	BBE	(bzd,arh,rma)	3
	BBH	(pay,(gym,(tfr,bzd),arh,rma))	6
	BBW	(bzd,arh,rma)	3
	BPE	(bzd,arh,rma)	3
	BPH	(pay,(gym,(tfr,bzd),arh,rma))	6
	BPW	(bzd,arh,rma)	3
	MBE	(sab,bzd,arh,rma)	4
	MBH	(pay,(gym,(tfr,bzd),arh,rma))	6
	MBW	(bzd,arh,rma)	3
	MPE	(sab,bzd,arh,rma)	4
	MPH	(pay,(gym,(tfr,bzd),arh,rma))	6
Chukotko-	BBE	(ckt,itl)	2

Family	Case	Structure	Size
**Kamchatkan**	**BBH**	((ckt,kpy),itl)	3
	**BPH**	((ckt,kpy),itl)	3
	**MBE**	((alr,ckt),itl)	3
	**MBH**	((ckt,kpy),itl)	3
	**MPE**	((alr,ckt),itl)	3
	**MPH**	((ckt,kpy),itl)	3
**Cochimi-Yuman**	**BBH**	(((coc,dih),mrc))	3
	**BPH**	(((coc,dih),mrc))	3
	**MBH**	(((coc,dih),mrc))	3
	**MPH**	(((coc,dih),mrc))	3
**Dravidian**	**BBE**	(brh,((kan,tam),tcy),tel)	5
	**BBH**	(kfb,tel,brh,((ggo,kff),(kan,(tam,mal),tcy)))	9
	**BBW**	(brh,(kan,tam,tcy),tel)	5
	**BPE**	(brh,kan)	2
	**BPH**	(tel,brh,(kff,(kan,(tam,mal),tcy)))	7
	**BPW**	(brh,(kan,tam),tel)	4
	**MBE**	((((bfq,kan),tam),tcy),brh,tel)	6
	**MBH**	(kfb,tel,brh,((ggo,kff),(kan,(tam,mal),tcy)))	9
	**MBW**	(brh,(kan,tam,tcy),tel)	5
	**MPE**	((((bfq,kan),tam)),brh,tel)	5
	**MPH**	(tel,brh,(kff,(kan,(tam,mal),tcy)))	7
**Eskimo-Aleut**	**BBH**	(kal,(esu,ess))	3
	**BPE**	(kal,esu)	2
	**BPH**	(kal,(esu,ess))	3
	**MBH**	(kal,(esu,ess))	3
	**MPE**	(ale,(kal,esu))	3
	**MPH**	(kal,(esu,ess))	3
**Gunwinyguan**	**BBH**	(gup,nuy,wrz)	3
	**BPH**	(gup,nuy,nig,wrz)	4
	**MBH**	(gup,nuy,wrz)	3

Family	Case	Structure	Size
	MPH	(gup,nuy,nig,wrz)	4
Hokan	BBE	((dih,mrc),(kyh,pom))	4
	BBW	((dih,mrc),pom)	3
	BPE	((coc,mrc),(kyh,pom))	4
	BPW	((dih,mrc),pom)	3
	MBE	(((acv,kyh),pom),(dih,mrc))	5
	MBW	((dih,mrc),pom)	3
	MPE	(((acv,kyh),pom),(dih,mrc))	5
Indo-European	BBE	(aln,hye,(bre,gle),(bul,pol,rus),((((((cat,spa),fra)),ita),ron)),((eng,deu),(isl,(nor,swe))),ell,(((hin,pan),kas,mar,nep,sin),((kmr,pes),pst)),(lav,lit))	29
	BBH	(aln,hye,(lit,lav),(((bre,cor,cym),(gla,gle))),(((nor,dan,swe),isl),(eng,deu,nld)),ell,(guj,pan,lmn,((urd,hin),(ben,mai),nep,kas,sin,mar),((oss,pst),(kmr,pes))),(ita,(fra,(cat,(spa,por)),ron)),((rus,ukr),bul,(ces,pol,hrv)))	44
	BBW	(aln,hye,(bre,gle),(bul,pol,rus),(cat,fra,ita,ron,spa),(eng,deu,isl,nor,swe),ell,(hin,kas,mar,nep,pan,sin),(kmr,pst,pes),(lav,lit))	29
	BPE	(aln,hye,(bre,gle),(bul,pol,rus),((eng,deu),(isl,(nor,swe))),(((fra,spa),ron)),ell,((hin,kas),(pes,pst)),(lav,lit))	22
	BPH	(aln,hye,(lit,lav),(((bre,cym),(gla,gle))),(((nor,dan,swe),isl),(eng,deu,nld)),ell,(pan,((urd,hin),(ben,mai),nep,kas,sin,mar),((oss,pst),(kmr,pes))),(ita,(fra,(cat,(spa,por)),ron)),((rus,ukr),bul,(ces,pol,hrv)))	41
	BPW	(aln,hye,(bre,gle,cym),(bul,pol,rus),(cat,fra,ita,ron,spa),(nld,eng,deu,isl,nor,swe),ell,(hin,kas,nep,pan,sin),(kmr,pst,pes),(lav,lit))	30
	MBE	(((afr,eng,deu),(isl,(nor,swe))),aln,hye,(bre,gle),(bul,pol,rus),((((((cat,spa),fra)),ita),ron)),ell,(((hin,pan),kas,mar,nep,sin),((kmr,pes),pst)),(lav,lit))	30
	MBH	(aln,hye,(lit,lav),(((bre,cor,cym),(gla,gle))),(((nor,dan,swe),isl),(eng,deu,nld)),ell,(guj,pan,lmn,((urd,hin),(ben,mai),nep,kas,sin,mar),((oss,pst),(kmr,pes))),(ita,(fra,(cat,(spa,por)),ron)),((rus,ukr),bul,(ces,pol,hrv)))	44
	MBW	(aln,hye,(bre,gle),(bul,pol,rus),(cat,fra,ita,ron,spa),(eng,deu,isl,nor,swe),ell,(hin,kas,mar,nep,pan,sin),(kmr,pst,pes),(lav,lit))	29
	MPE	((((afr,nld),eng,deu),(isl,(nor,swe))),aln,hye,(((bre,cym),gle)),(bul,pol,rus),((((((cat,spa),fra)),ita),ron)),ell,(((hin,pan),kas,nep,sin),((kmr,pes),pst)),(lav,lit))	31
	MPH	(aln,hye,(lit,lav),(((bre,cym),(gla,gle))),(((nor,dan,swe),isl),(eng,deu,nld)),ell,(pan,((urd,hin),(ben,mai),nep,kas,sin,mar),((oss,pst),(kmr,pes))),(ita,(fra,(cat,(spa,por)),ron)),((rus,ukr),bul,(ces,pol,hrv)))	41
	MPW	(aln,hye,(bul,pol,rus),(eng,deu,swe),(fra,ron,spa),ell,(hin,kas),gle,(lav,lit),(pst,pes))	19
Iroquoian	BBE	(chr,one)	2
	BBH	((see,one),chr)	3
	BPH	((see,one),chr)	3
	MBH	((see,one),chr)	3
	MPE	(chr,(one,see))	3
	MPH	((see,one),chr)	3
Je-Jabuti	BBH	(kgp,(apn,ram))	3

Family	Case	Structure	Size
	BPH	(kgp,(apn,ram))	3
	MBH	(kgp,(apn,ram))	3
	MPH	(kgp,(apn,ram))	3
Khoisan	BBE	(((hnh,naq),ktz))	3
	BBW	((hnh,naq),ktz)	3
	BPE	(ktz,naq)	2
	BPW	((hnh,naq),ktz)	3
	MBE	(((hnh,naq),ktz))	3
	MBW	((hnh,naq),ktz)	3
	MPE	(((hnh,naq),ktz))	3
Macro-Ge	BBE	(bor,(ram,kgp))	3
	BPE	(bor,ram)	2
	BPW	(bor,ram)	2
	MBE	(((apn,ram),kgp),bor)	4
	MBW	(bor,(ram,kgp))	3
	MPE	((apn,ram),bor)	3
Mande	BBH	((daf,mev),(bam,(xpe,men)))	5
	BPH	(daf,((((((bam,mlq),vai))),(xpe,men))))	6
	MBH	((daf,mev),(bam,(xpe,men)))	5
	MPH	(daf,(((bam,vai),(xpe,men))))	5
Mataco-Guaicuru	BBE	(axb,mzh)	2
	BPE	(axb,mzh)	2
Mayan	BBE	(hsf,jac)	2
	BBH	(((chf,jac,tzj),yua))	4
	BPH	(((jac,tzj),yua))	3
	MBE	(acc,hsf,jac)	3
	MBH	(((chf,jac,tzj),yua))	4
	MPE	(((acc,tzj),mvc),hsf,jac)	5
	MPH	(((jac,tzj),yua))	3
Mongolic	BBH	(dta,mjg,(((bxm,khk),xal)),mhj)	6

Family	Case	Structure	Size
	BPH	(dta,mjg,(((bxm,khk),xal)))	5
	MBH	(dta,mjg,(((bxm,khk),xal)),mhj)	6
	MPH	(dta,mjg,(((bxm,khk),xal)))	5
Muskogean	MPH	((cku,akz),cho)	3
Na-Dene	BBE	(hdn,((hup,nav,scs),tli))	5
	BBW	((hup,nav,scs),tli)	4
	BPE	(hdn,((nav,scs),tli))	4
	BPW	((hup,nav,scs),tli)	4
	MBE	(((aht,hup,nav,scs),tli),hdn)	6
	MBW	((hup,nav,scs),tli)	4
	MPE	(((aht,hup,nav,scs),tli),hdn)	6
	MPW	((nav,scs),tli)	3
Nakh-Daghestanian	BBH	(((ava,huz),lbe,(aqc,(lez,rut))),(bbl,(inh,che)))	9
	BBW	((aqc,lez,rut),(ava,huz),(inh,bbl),lbe)	8
	BPH	(((gdo,ava,huz),lbe,(aqc,(lez,rut))),(bbl,(inh,che)))	10
	BPW	((aqc,lez),(ava,huz),(inh,bbl),lbe)	7
	MBH	(((ava,huz),lbe,(aqc,(lez,rut))),(bbl,(inh,che)))	9
	MBW	((aqc,lez,rut),(ava,huz),(inh,bbl),lbe)	8
	MPH	(((ava,huz),lbe,(aqc,(lez,rut))),(bbl,(inh,che)))	9
	MPW	(huz,inh,lez)	3
Niger-Congo	BBE	((((aka,ewe),((bom,pym),((ewo,(lue,cgg,swh,zul),nhu)),ibo,yor),(((dag,kfz),spp),((dow,mzm),(gbp,zne))),gry),((dyo,ndv,wol),(kqs,tem)),ijc),(bam,daf),mor)	29
	BBW	((aka,ewe),bam,(bom,pym),(dag,kfz,spp),daf,(dyo,ndv,wol),(dow,gbp,mzm,sag,zne),(ewo,lue,cgg,nhu,swh,zul),gry,ibo,ijc,(kqs,tem),mor,yor)	30
	BPE	((((aka,ewe),gry,(ibo,(lue,cgg,swh,zul),yor),(kfz,spp)),(dyo,wol),ijc),bam)	15
	BPW	((aka,ewe),bam,bom,(dag,kfz,spp),(dyo,fuv,wol),(dow,gbp,mzm,sag,zne),(ewo,lue,cgg,swh,zul),gry,ibo,ijc,(kqs,tem),mor,yor)	27
	MBE	(((((abi,aka),ewe),((bom,pym),((ewo,(lue,cgg,swh,zul),nhu)),ibo,yor),(((dag,kfz),spp),((dow,mzm),(gbp,zne))),gry),((dyo,ndv,wol),(kqs,tem)),ijc),(bam,daf),mor)	30
	MBW	((aka,ewe),bam,(bom,pym),(dag,kfz,spp),daf,(dyo,ndv,wol),(dow,gbp,mzm,sag,zne),(ewo,lue,cgg,nhu,swh,zul),gry,ibo,ijc,(kqs,tem),mor,yor)	30
	MPE	(((((abi,aka),ewe),(bom,(ewo,(lue,cgg,swh,zul)),ibo,yor),(((dag,kfz),spp),((dow,mzm),(gbp,zne))),gry),(dyo,(fuv,wol),(kqs,tem)),ijc),bam,mor)	27
	MPW	((aka,ewe),bam,(dyo,wol),(gbp,sag),gry,ibo,ijc,(kfz,spp),(lue,cgg,swh,zul),yor)	17
Nilo-Saharan	BBE	(bmi,(lgg,niy),wti,((dip,(((laj,luo),mde)),mas,niq),ikx,(mur,nrb,kzh)),fvr,(khq,ses),kun,knc,kgo)	20
	BBW	(bmi,wti,(dip,laj,luo,mas,niq),fvr,ikx,knc,(khq,ses),kun,lgg,mde,mur,nrb,niy,kzh)	19

Family	Case	Structure	Size
	BPE	(bmi,(lgg,niy),fvr,(khq,ses),kun,knc,kgo,(((((laj,luo),mde)),niq),(mur,kzh)))	15
	BPW	(bmi,(dip,laj,luo,mas,niq,tuv),fvr,ikx,knc,(khq,ses),kun,lgg,mde,mur,nrb,niy,kzh)	19
	MBE	((((((((ach,laj),luo)),mde),dip),mas,niq),ikx,(mur,nrb,kzh)),(bmi,(lgg,niy)),wti,fvr,(khq,ses),kun,knc,kgo)	21
	MBW	(bmi,wti,(dip,laj,luo,mas,niq),fvr,ikx,knc,(khq,ses),kun,lgg,mde,mur,nrb,niy,kzh)	19
	MPE	((((((((ach,laj),luo)),mde),dip),(mas,tuv),niq),ikx,(mur,nrb,kzh)),(bmi,(lgg,niy)),fvr,(khq,ses),kun,knc,kgo)	21
	MPW	(bmi,fvr,knc,(khq,ses),kun,(laj,luo,mas,niq),lgg,mde,mur,niy,kzh)	15
Nilotic	BBH	(bfa,(mas,tuv),(kpz,niq),(dip,(lkr,(luo,laj))))	9
	BPH	(bfa,(mas,tuv),(kpz,niq),(dip,(lkr,(luo,laj))))	9
	MBH	(bfa,(mas,tuv),(kpz,niq),(dip,(lkr,(luo,laj))))	9
	MPH	(bfa,(mas,tuv),(kpz,niq),(dip,(lkr,(luo,laj))))	9
North Caucasian	BBE	((abk,kbd),(aqc,(lez,rut),ava,huz,(inh,bbl),lbe))	10
	BPE	(abk,(ava,huz,inh,lbe,lez))	6
	MBE	((abk,kbd),(aqc,(lez,rut),ava,huz,(inh,bbl),lbe))	10
	MPE	((abk,kbd),((aqc,lez),ava,huz,(inh,bbl),lbe))	9
North Halmahera	BBH	((saj,tvo),mqs)	3
	BPH	((saj,tvo),mqs)	3
	MBH	((saj,tvo),mqs)	3
	MPH	((saj,tvo),mqs)	3
Oto-Manguean	BBE	((chq,cle),ctp,mig,ote)	5
	BBH	(((((mig,mil),trc)),(maq,(ctp,zai))),((cpa,cle,(chq,cco),ote),tpx))	12
	BBW	(ctp,(cle,chq),mig,ote)	5
	BPE	(cle,mig,ote)	3
	BPH	(((((mig,mil),trc)),(maq,(ctp,zai))),((cle,(chq,cco),ote),tpx))	11
	BPW	(ctp,cle,mig,ote)	4
	MBE	(amu,(chq,cle),ctp,mig,ote)	6
	MBH	(((((mig,mil),trc)),(maq,(ctp,zai))),((cpa,cle,(chq,cco),ote),tpx))	12
	MBW	(ctp,(cle,chq),mig,ote)	5
	MPE	(amu,cle,ctp,mig,ote)	5
	MPH	(((((mig,mil),trc)),(maq,(ctp,zai))),((cle,(chq,cco),ote),tpx))	11
	MPW	(cle,mig,ote)	3

Family	Case	Structure	Size
**Pama-Nyungan**	**BBH**	((aly,aer),dbl,ktg,kky,dif,wim,(vma,pjt),jao,wyb,gvn,yii,duj)	14
	**BPH**	((aly,aer),dbl,ktg,kgs,kky,dif,wim,(vma,pjt),wyb,gvn,yii,duj)	14
	**MBH**	((aly,aer),dbl,ktg,kky,dif,wim,(vma,pjt),jao,wyb,gvn,yii,duj)	14
	**MPH**	((aly,aer),dbl,ktg,kky,dif,wim,(vma,pjt),wyb,gvn,yii,duj)	13
**Panoan**	**BBH**	(cao,shp,amc)	3
	**BPH**	(cao,shp,amc)	3
	**MBH**	(cao,shp,amc)	3
	**MPH**	(cao,shp,amc)	3
**Penutian**	**BBE**	(csz,(kla,nez),nmu,skd,tsi,wit)	7
	**BBW**	(kla,nmu,skd,nez,tsi,wit)	6
	**BPE**	(csz,nmu,skd,nez,tsi,wit)	6
	**BPW**	(kla,nmu,skd,nez,tsi,wit)	6
	**MBE**	((aes,csz),(kla,nez),nmu,skd,tsi,wit)	8
	**MBW**	(kla,nmu,skd,nez,tsi,wit)	6
	**MPE**	((aes,csz),(kla,nez),nmu,skd,tsi,wit)	8
	**MPW**	(nmu,skd,nez,tsi,wit)	5
**Salishan**	**BBE**	(blc,shs,squ)	3
	**BBH**	(blc,(hur,squ),shs,cjh)	5
	**BPH**	(blc,(hur,squ),shs)	4
	**BPW**	(shs,squ)	2
	**MBE**	(blc,shs,squ)	3
	**MBH**	(blc,(hur,squ),shs,cjh)	5
	**MBW**	(blc,shs,squ)	3
	**MPE**	(blc,shs,squ)	3
	**MPH**	(blc,(hur,squ),shs)	4
**Sepik**	**BBE**	(amp,kmn,kmo)	3
	**BBH**	(kmo,kmn,amp,yss)	4
	**BPH**	(kmo,kmn,amp,yss)	4
	**BPW**	(amp,kmn)	2
	**MBE**	(aau,amp,kmn,kmo)	4

Family	Case	Structure	Size
	**MBH**	(kmo,kmn,amp,yss)	4
	**MBW**	(amp,kmn,kmo)	3
	**MPE**	(aau,amp,kmn)	3
	**MPH**	(kmo,kmn,amp,yss)	4
**Sino-Tibetan**	**BBE**	((njo,(bgr,ctd),((brx,grt),kac),mya,(ksw,eky),(((lbj,taj),lep),(lif,new)),lhu,mni,dap),(yue,cmn))	19
	**BBH**	(cmn,(hak,yue),(bca,((lhu,mya),nbf),(((cdm,kgj),(byw,lif,dus),new),lep,((taj,ggn),bee)),(sip,(bod,lbj)),(kac,(brx,grt)),(ksw,(eky,bwe)),((((cnh,bgr),lus),ctd), (njo,nsm,mni)),raw,dap))	35
	**BBW**	((njo,bgr,ctd,mni),(brx,grt),(mya,lhu),(yue,cmn),kac,(ksw,eky),(lbj,lif,new,taj),lep,dap)	19
	**BPE**	(bgr,mya,grt,eky,((((lbj,bod),lep)),lhu,mni),(yue,cmn))	11
	**BPH**	(cmn,(hak,yue),(bca,(lhu,mya),(((cdm,kgj),(lif,dus),new),lep,(taj,bee)),(bod,lbj),(kac,(brx,grt)),(ksw,eky),((mrh,((cnh,bgr),lus),ctd),(njo,nsm,mni)),raw,dap))	31
	**BPW**	((njo,bgr,mni),(brx,grt),(mya,lhu),(yue,cmn),eky,(lbj,lif),lep)	13
	**MBE**	(((acn,mya),(njo,(bgr,ctd)),((brx,grt),kac),(ksw,eky),(((lbj,taj),lep),(lif,new)),lhu,mni,dap),(yue,cmn))	20
	**MBH**	(cmn,(hak,yue),(bca,((lhu,mya),nbf),(((cdm,kgj),(byw,lif,dus),new),lep,((taj,ggn),bee)),(sip,(bod,lbj)),(kac,(brx,grt)),(ksw,(eky,bwe)),((((cnh,bgr),lus),ctd), (njo,nsm,mni)),raw,dap))	35
	**MBW**	((njo,bgr,ctd,mni),(brx,grt),(mya,lhu),(yue,cmn),kac,(ksw,eky),(lbj,lif,new,taj),lep,dap)	19
	**MPE**	(((acn,mya),(njo,bgr),(brx,grt),eky,((lbj,lep),lif),lhu,mni),(yue,cmn))	14
	**MPH**	(cmn,(hak,yue),(bca,(lhu,mya),((kgj,(lif,dus),new),lep,(taj,bee)),(bod,lbj),(kac,(brx,grt)),(ksw,eky),((((cnh,bgr),lus),ctd),(njo,mni)),raw,dap))	28
	**MPW**	((bgr,mni),(mya,lhu),(yue,cmn),grt,eky,lbj,lep)	10
**Tacanan**	**BBE**	(aro,tna)	2
**Tai-Kadai**	**BBE**	(nut,tha)	2
	**BBH**	((pcc,nut,(lao,tha,shn),doc))	6
	**BPH**	((pcc,nut,(lao,tha),doc))	5
	**MBE**	(doc,(nut,tha))	3
	**MBH**	((pcc,nut,(lao,tha,shn),doc))	6
	**MPE**	(doc,(nut,tha))	3
	**MPH**	((nut,(lao,tha),doc))	4
**Trans-New Guinea**	**BBE**	(((aey,wnu),kpw,tya),tml,(dgz,yrb),(dni,ekg),hmt,kew,kiw,mrz,sue,mtg,wgi,ygr)	17
	**BBW**	((aey,kpw,tya,wnu),tml,dni,ekg,hmt,kew,sue,mtg,wgi,ygr)	13
	**BPE**	(((aey,wnu),kpw),tml,dgz,(dni,ekg),hmt,kew,mrz,sue,mtg)	12
	**MBE**	((agd,ygr),((aey,wnu),kpw,tya),tml,(dgz,yrb),(dni,ekg),hmt,kew,kiw,mrz,sue,mtg,wgi)	18
	**MPE**	((agd,ygr),((aey,wnu),kpw,tya,wsk),tml,dgz,(dni,ekg),hmt,kew,mrz,sue,mtg)	16

Family	Case	Structure	Size
	MPW	((aey,kpw,tya,wnu),tml,dni,hmt,kew,sue,mtg,ygr)	11
	BBH	(sue,(tya,(wsk,wnu),(aey,ssd)),(tml,((wms,tyn),((sll,wgi),gaj,kew,ygr,kpw),spl,(dni,ekg))),mtg)	19
	BPH	(sue,(tya,(wsk,wnu),(aey,ssd)),(tml,((wms,tyn),((sll,wgi),gaj,kew,ygr,kpw),spl,(dni,ekg))),mtg)	19
	MBH	(sue,(tya,(wsk,wnu),(aey,ssd)),(tml,((wms,tyn),((sll,wgi),gaj,kew,ygr,kpw),spl,(dni,ekg))),mtg)	19
	MPH	(sue,(tya,(wsk,wnu),(aey,ssd)),(tml,((wms,tyn),((sll,wgi),gaj,kew,ygr,kpw),spl,(dni,ekg))),mtg)	19
**Tucanoan**	BBE	(bsn,cub)	2
	BBH	((cub,tnc),bsn,snn)	4
	BBW	(bsn,cub)	2
	BPH	((cub,tnc),(tuo,bsn),snn)	5
	MBE	((bao,bsn),cub)	3
	MBH	((cub,tnc),bsn,snn)	4
	MPE	((bao,bsn),cub,tnc)	4
	MPH	((cub,tnc),(tuo,bsn),snn)	5
**Tungusic**	BBH	(eve,evn,(gld,ude),mnc)	5
	BPH	(eve,evn,(gld,ude),mnc)	5
	MBH	(eve,evn,(gld,ude),mnc)	5
	MPH	(eve,evn,(gld,ude),mnc)	5
**Tupian**	BBE	(gug,srq,urb)	3
	BPE	(gug,urb)	2
	MBE	(((guq,gug),srq,urb))	4
	MPE	(((guq,gug),srq,urb))	4
	BBH	(gug,srq,cod,kay,urb)	5
	BBW	(gug,srq,urb)	3
	BPH	(gug,srq,cod,urb)	4
	MBH	(gug,srq,cod,kay,urb)	5
	MPH	(gug,srq,urb)	3
**Turkic**	BBH	(chv,(((tur,azb,(krc,(tat,bak),(kaa,kir)),uzn)),sah,tyv))	11
	BPH	(chv,(((tur,azb,((tat,bak),kir),uzn)),sah,tyv))	9
	MBH	(chv,(((tur,azb,(krc,(tat,bak),(kaa,kir)),uzn)),sah,tyv))	11
	MPH	(chv,(((tur,azb,((tat,bak),kir),uzn)),sah,tyv))	9

Family	Case	Structure	Size
Uralic	BBE	(fin,hun,kca,kpv,(yrk,sel))	6
	BBH	((est,fin),hun,kca,mns,mhr,(udm,kpv),sme,(yrk,sel,nio))	12
	BBW	((fin,kpv),(hun,kca),(yrk,sel))	6
	BPE	(fin,hun,kca,yrk)	4
	BPH	((est,fin),hun,kca,mns,mhr,kpv,sme,(yrk,sel,nio))	11
	BPW	(fin,(hun,kca),yrk)	4
	MBE	((enf,yrk,sel),fin,hun,kca,kpv)	7
	MBH	((est,fin),hun,kca,mns,mhr,(udm,kpv),sme,(yrk,sel,nio))	12
	MBW	((fin,kpv),(hun,kca),(yrk,sel))	6
	MPE	((enf,yrk),fin,hun,kca)	5
	MPH	((est,fin),hun,kca,mns,mhr,kpv,sme,(yrk,sel,nio))	11
	MPW	(fin,hun,yrk)	3
Uto-Aztecan	BBE	((chl,com,hop),((nhg,ppl),(ood,yaq)))	7
	BBH	((hop,((par,com),xaw,pao),(chl,lui)),(crn,(ppl,(nhg,ncj)),(ntp,ood),yaq))	14
	BBW	(chl,com,hop,(nhg,ppl),ood,yaq)	7
	BPE	((chl,com),((nhg,ppl),(ood,yaq)))	6
	BPH	((hop,((par,com),(xaw,ute),pao),(chl,lui)),(crn,(ppl,(nhg,ncj)),(ntp,ood),yaq))	15
	BPW	((chl,lui),(com,par),hop,(nhg,ppl),ood,yaq)	9
	MBE	((chl,com,hop),((nhg,ppl),(ood,yaq)))	7
	MBH	((hop,((par,com),xaw,pao),(chl,lui)),(crn,(ppl,(nhg,ncj)),(ntp,ood),yaq))	14
	MBW	(chl,com,hop,(nhg,ppl),ood,yaq)	7
	MPE	(((chl,lui),(com,par),hop),((nhg,ppl),(ood,yaq)))	9
	MPH	((hop,((par,com),(xaw,ute),pao),(chl,lui)),(crn,(ppl,(nhg,ncj)),(ntp,ood),yaq))	15
	MPW	(chl,com,(nhg,ppl),ood,yaq)	6
Wakashan	BBE	(kwk,myh)	2
	MBE	((hei,kwk),myh)	3
	MPE	((hei,kwk),(myh,noo))	4
West Papuan	BBE	(tvo,mqs)	2
	MBE	(gbi,tvo,mqs)	3
Yanomam	BBE	(shb,xsu)	2

Family	Case	Structure	Size
**Yukaghir**	**BBE**	(yux,ykg)	2

Table S4: *The composition and structure of the language families can vary among cases. The structure is given as the topology of the language family in the parentheses notation (Newick format; http://evolution.genetics.washington.edu/phylip/newicktree.html). The languages are given using their ISO 639-2 three-letter codes. The number of families and their composition might differ slightly among outgroups for MrBayes.*

Case	Using Pearson's *r*		Using Spearmans' ρ	
	r	*p*	ρ	*p*
**MBE**	0.21	0.0017	0.24	$< 10^{-4}$
**MBW**	0.30	$< 10^{-4}$	0.32	$3 \cdot 10^{-4}$
**MBH**	0.19	0.001	0.22	$9.99 \cdot 10^{-5}$
**MPE**	0.20	0.0031	0.22	0.0012
**MPW**	0.08	0.19	0.10	0.13
**MPH**	0.19	0.0008	0.19	0.0002
**BBE**	0.32	$< 10^{-4}$	0.30	$< 10^{-4}$
**BBW**	0.30	$2 \cdot 10^{-4}$	0.31	$< 10^{-4}$
**BBH**	0.07	0.14	0.07	0.12
**BPE**	0.30	$4 \cdot 10^{-4}$	0.23	0.0011
**BPW**	0.14	0.046	0.16	0.022
**BPH**	0.04	0.22	0.05	0.20

Table S5*: The Mantel correlation (Pearson's r and Spearman's ρ) between stability and geographical distances (10,000 permutations).*

Feature	Involvement
**Tone2**	**0.99**
**PolQPart**	**0.99**
**AssocPlu**	**0.99**
**SymAsymStNeg2**	**0.98**
**OccNPlu**	**0.98**
**SV2**	**0.98**
**AbsComC**	**0.98**
**OrdNum**	**0.98**
**Perfect**	**0.98**
**LatC**	**0.98**
**OV2**	**0.98**
**GenDIPersP**	**0.98**
**NumN2**	**0.98**
**Cons1**	**0.98**
**IntReflPron**	**0.97**
**IndefArt**	**0.97**
SylStr1	0.79
PastTense	0.79
OlbPosInfl	0.79
VpersM	0.79
SuppTAsp	0.79
AdjN1	0.79
Cons2	0.79
SexGen	0.78
ComInstr	0.22
LmarkPNP	0.21

Table S6*: Most involved features for dataset* ***MBE*** *(showing only those with involvement > 0.03). The PC1 of the 5 runs explains* ***92.22%*** *of variance and the maximum Mantel correlation is* ***0.51****.*

Feature	Involvement
**IndefArt**	**0.99**
**AssocPlu**	**0.99**
**OrdNum**	**0.98**
**IntReflPron**	**0.98**
**NomVConj**	**0.98**
**AbsComC**	**0.98**
**OlbPosInfl**	**0.98**
**Cons1**	**0.98**
**LatC**	**0.98**
**VpersM**	**0.98**
**P3PrDem**	**0.98**
**OVAdpNP**	**0.98**
**Tone2**	**0.97**
**Perfect**	**0.97**
OccNPlu	0.79
PastTense	0.79
SV2	0.79
PerfImpAsp	0.79
SynVPNM	0.78
OvSitEpi	0.77
LmarkC	0.60
FixStress	0.59
SylStr1	0.41
MorphImp	0.41
LmarkPNP	0.41
PolQPart	0.41
IntPhCQ2	0.40
GlotC	0.40
IntPhCQ1	0.40
DistNum	0.40
DefArt	0.40
SymAsymStNeg2	0.22
NumN1	0.22
SylStr2	0.21
NumN2	0.21
NumClas	0.21
NoGen	0.21
ConjUQu	0.21

***Table S7**: Most involved features for dataset **MBW** (showing only those with involvement > 0.03). The PC1 of the 5 runs explains **73.60%** of variance and the maximum Mantel correlation is **0.62**.*

Feature	Involvement
**PadDem**	**0.98**
**IndefArt**	**0.98**
**LatC**	**0.98**
**OV**	**0.98**
**WhenC**	**0.98**
**NomVConj**	**0.98**
**OvSitEpi**	**0.98**
**Cons**	**0.98**
**SV**	**0.98**
**PastTense**	**0.98**
**Perfect**	**0.97**
**AbsComC**	**0.97**
**NumN**	**0.97**
**PersMV**	**0.97**
**PersMAdpos**	**0.97**
**ApplicativeC**	**0.96**
**OlbPosInfl**	**0.96**

Table S8*: Most involved features for dataset* ***MPE*** *(showing only those with involvement > 0.05). The PC1 of the 5 runs explains* ***99.93%*** *of variance and the maximum Mantel correlation is* ***0.48****.*

Feature	Involvement
**Redup**	**0.98**
**ApplicativeC**	**0.98**
**AntipassiveC**	**0.97**
**Perfect**	**0.97**
**NomLocPred**	**0.97**
**SV**	**0.97**
**AbsComC**	**0.97**
**MorphImp**	**0.97**
**VpersM**	**0.97**
**DefArt**	**0.97**
**WhenC**	**0.97**
**CVRatio**	**0.97**
**PastTense**	**0.97**
**DegWAdj**	**0.97**
**OlbPosInfl**	**0.95**
IndefArt	0.78
NumN	0.77
SymAsymStNeg	0.60
NomVConj	0.59
P3PrDem	0.59
IntPhCQ	0.41
LatC	0.41
LmarkPNP	0.41
OVAdpNP	0.40
NMPron	0.40
PredAdj	0.40
AdposNP	0.22
HandArm	0.22
NumClas	0.22

***Table S9**: Most involved features for dataset **MPW** (showing only those with involvement > 0.04). The PC1 of the 5 runs explains **80.83%** of variance and the maximum Mantel correlation is **0.63**.*

Feature	Involvement
**GenDIPersP**	**0.98**
**IndefArt**	**0.97**
**NomVConj**	**0.96**
IflSVerb	0.79
ConjUQu	0.22

***Table S10**: Most involved features for dataset **BBE** (showing only those with involvement > 0.03). The PC1 of the 5 runs explains **91.88%** of variance and the maximum Mantel correlation is **0.50**.*

Feature	Involvement
**PastTense**	**0.99**
**LmarkPNP**	**0.99**
**NumN1**	**0.99**
**P3PrDem**	**0.99**
**ConjUQu**	**0.99**
**LatC**	**0.98**
**SymAsymStNeg2**	**0.98**
**FixStress**	**0.98**
**OVAdpNP**	**0.98**
**SylStr1**	**0.98**
**IntPhCQ2**	**0.98**
**PolQPart**	**0.98**
**VoicPF2**	**0.98**
**SexGen**	**0.98**
GenN1	0.79
NomVConj	0.79
IndefArt	0.78
OccNPlu	0.40
Cons1	0.22
AbsComC	0.21
OlbPosInfl	0.21
OrdNum	0.21
NumN2	0.21

Table S11*: Most involved features for dataset* ***BBW*** *(showing only those with involvement > 0.03). The PC1 of the 5 runs explains* ***89.90%*** *of variance and the maximum Mantel correlation is* ***0.58****.*

Feature	Involvement
**IntPhCQ**	**0.98**
**PerfImpAsp**	**0.98**
**MTPron**	**0.98**
**P3PrDem**	**0.98**
**NomVConj**	**0.98**
**AbsComC**	**0.98**
**Cons**	**0.98**
**VoicPF**	**0.98**
**OvSitEpi**	**0.97**
**PolitDPron**	**0.97**
**ZeroCopPredNom**	**0.97**
**Ncases**	**0.97**
**OlbPosInfl**	**0.97**
**GenDIPersP**	**0.97**
**AntipassiveC**	**0.97**
**PresUnC**	**0.97**
**NumN**	**0.97**
**NumClas**	**0.96**

***Table S12**: Most involved features for dataset **BPE** (showing only those with involvement > 0.03). The PC1 of the 5 runs explains **99.95%** of variance and the maximum Mantel correlation is **0.45**.*

Feature	Involvement
**MTPron**	**0.98**
**IntPhCQ**	**0.98**
**VowelN**	**0.98**
**OlbPosInfl**	**0.98**
**NumN**	**0.98**
**PastTense**	**0.98**
**LmarkC**	**0.98**
**NomVConj**	**0.97**
**AbsComC**	**0.97**
**OvSitEpi**	**0.96**
FutTense	0.79
ZeroCopPredNom	0.79
Cons	0.78
VpersM	0.78
FixStress	0.78
PersMV	0.60
PadDem	0.60
OV	0.59
P3PrDem	0.41
WhenC	0.41
PersMAdpos	0.40

Table S13*: Most involved features for dataset* ***BPW*** *(showing only those with involvement > 0.03). The PC1 of the 5 runs explains* ***84.30%*** *of variance and the maximum Mantel correlation is* ***0.40****.*

Methods		Correlation		H_0 rejection (α=0.05) concordance					
				Contingency table				χ^2 test	
		r	*p*	T-T	F-F	T-F	F-T	$\chi^2(1)$	*p*
Fisher	Z-transform	0.92	$< 2.2 \bullet 10^{-16}$	29	18	0	3	35.21	$2.96 \bullet 10^{-9}$
	Hartung	0.92	$< 2.2 \bullet 10^{-16}$	25	18	0	7	25.09	$5.48 \bullet 10^{-7}$
	Makambi	0.99	$< 2.2 \bullet 10^{-16}$	30	18	0	2	38.37	$5.85 \bullet 10^{-10}$
	Simes	-	-	23	18	0	9	21.15	$4.24 \bullet 10^{-6}$
Z-transform	Hartung	0.97	$< 2.2 \bullet 10^{-16}$	24	20	1	5	26.60	$2.50 \bullet 10^{-7}$
	Makambi	0.90	$< 2.2 \bullet 10^{-16}$	27	18	3	2	28.33	$1.02 \bullet 10^{-7}$
	Simes	-	-	20	18	3	9	12.54	0.0004
Hartung	Makambi	0.93	$< 2.2 \bullet 10^{-16}$	25	20	5	0	30.08	$4.14 \bullet 10^{-8}$
	Simes	-	-	21	23	2	4	26.09	$3.26 \bullet 10^{-7}$
Simes	Makambi	-	-	23	20	7	0	25.39	$4.68 \bullet 10^{-7}$

Table S14*: Correlations and concordances between methods for combining p-values. There is very high agreement between these methods on our data. The* **correlation** *represents the Pearson's correlation coefficient between the combined p-values provided by the methods (please note that Simes does not compute a combined p-value and only provides a decision to reject/not reject* H_0 *at the chosen α-level). If we take the α-level to be 0.05, then these tests reject or not the* H_0 *and the columns* ***T-T****,* ***F-F****,* ***T-F*** *and* ***F-F*** *represent the number of times the two tests both reject* H_0*, both fail to reject it, only the first does reject and only the second, respectively. The last two columns show the* χ^2 *test on this contingency table.*

Macro-area	Interpretation	Dataset	Composition
**Africa**[1]	Do all the African language families form a group?	**MBE**	*Afro-Asiatic, Khoisan, Niger-Congo, Nilo-Saharan*
		**MBW**	*Afro-Asiatic, Khoisan, Niger-Congo, Nilo-Saharan*
		**MBH**	*Afro-Asiatic, Atlantic-Congo, Central Sudanic, Mande, Nilotic*
		**MPE**	*Afro-Asiatic, Khoisan, Niger-Congo, Nilo-Saharan*
		**MPW**	**[no *Khoisan* in this dataset]**
		**MPH**	*Afro-Asiatic, Atlantic-Congo, Central Sudanic, Mande, Nilotic*
		**BBE**	*AfroAsiatic, Khoisan, NigerCongo, NiloSaharan*
		**BBW**	*AfroAsiatic, Khoisan, NigerCongo, NiloSaharan*
		**BBH**	*Afro-Asiatic, Atlantic-Congo, Central Sudanic, Mande, Nilotic*
		**BPE**	*AfroAsiatic, Khoisan, NigerCongo, NiloSaharan*
		**BPW**	*AfroAsiatic, Khoisan, NigerCongo, NiloSaharan*
		**BPH**	*Afro-Asiatic, Atlantic-Congo, Central Sudanic, Mande, Nilotic*
**America**	Do all the American language families form a group?	**MBE**	*Algic, Arawakan, Carib, Chibchan, Hokan, Macro-Ge, Mayan, Na-Dene, Oto-Manguean, Penutian, Salishan, Tucanoan, Tupi, Uto-Aztecan, Wakashan*
		**MBW**	*Algic, Arawakan, Cariban, Chibchan, Hokan, Macro-Ge, Na-Dene, Oto-Manguean, Penutian, Salishan, Uto-Aztecan*
		**MBH**	*Algic, Arawakan, Athapaskan-Eyak-Tlingit, Cariban, Chibchan, Cochimi-Yuman, Eskimo-Aleut, Iroquoian, Je-Jabuti, Mayan, Otomanguean, Panoan, Salishan, Tucanoan, Tupian, Uto-Aztecan*
		**MPE**	*Algic, Arawakan, Carib, Chibchan, Eskimo-Aleut, Hokan, Iroquoian, Macro-Ge, Mayan, Na-Dene, Oto-Manguean ,Penutian, Salishan, Tucanoan, Tupi, Uto-Aztecan, Wakashan*
		**MPW**	*Algic, Cariban, Na-Dene, Oto-Manguean, Penutian, Uto-Aztecan*
		**MPH**	*Algic, Arawakan, Athapaskan-Eyak-Tlingit, Cariban, Chibchan, Cochimi-Yuman, Eskimo-Aleut, Iroquoian, Je-Jabuti, Mayan, Otomanguean, Panoan, Salishan, Tucanoan, Tupian, Uto-Aztecan*
		**BBE**	*Algic, Arawakan, Aymaran, Carib, Chibchan, Hokan, Iroquoian, MacroGe, MatacoGuaicuru, Mayan, NaDene, OtoManguean, Penutian, Salishan, Tacanan, Tucanoan, Tupi, UtoAztecan, Wakashan, Yanomam*
		**BBW**	*Algic, Arawakan, Aymaran, Cariban, Chibchan, Hokan, NaDene, OtoManguean, Penutian, Tucanoan, Tupian, UtoAztecan*
		**BBH**	*Algic, Arawakan, Athapaskan-Eyak-Tlingit, Cariban, Chibchan, Cochimi-Yuman, Eskimo-Aleut, Iroquoian, Je-Jabuti, Mayan, Otomanguean, Panoan, Salishan, Tucanoan, Tupian, Uto-Aztecan*
		**BPE**	*Algic, Arawakan, Aymaran, Carib, Chibchan, EskimoAleut, Hokan, MacroGe, MatacoGuaicuru, NaDene, OtoManguean, Penutian, Tupi, UtoAztecan*
		**BPW**	*Algic, Arawakan, Cariban, Chibchan, Hokan, MacroGe, NaDene, OtoManguean, Penutian, Salishan, UtoAztecan*

1 Given that the **H** classification does not contain "*Khoisan*" or an equivalent grouping, for the four datasets using it (**MBH**, **MPH**, **BBH** and **BPH**) **Africa** and **Africa (w/o Khoisan)** are equivalent. We considered it equivalent to **Africa** as it conceptually tests the coherence of the whole continent with respect to the rest of the world.

Macro-area	Interpretation	Dataset	Composition
		**BPH**	*Algic, Arawakan, Athapaskan-Eyak-Tlingit, Cariban, Chibchan, Cochimi-Yuman, Eskimo-Aleut, Iroquoian, Je-Jabuti, Mayan, Otomanguean, Panoan, Salishan, Tucanoan, Tupian, Uto-Aztecan*
**S America (vs America)**	Do the South American language families form a group relative to the other American language families?	**MBE**	*Arawakan, Carib, Macro-Ge, Tucanoan, Tupi*
		**MBW**	*Arawakan, Cariban, Macro-Ge*
		**MBH**	*Arawakan, Cariban, Chibchan, Je-Jabuti, Panoan, Tucanoan, Tupian*
		**MPE**	*Arawakan, Carib, Macro-Ge, Tucanoan, Tupi*
		**MPW**	**[too few language families]**
		**MPH**	*Arawakan, Cariban, Chibchan, Je-Jabuti, Panoan, Tucanoan, Tupian*
		**BBE**	*Arawakan, Aymaran, Carib, MacroGe ,MatacoGuaicuru, Tucanoan, Tupi, Yanomam*
		**BBW**	*Arawakan, Aymaran, Cariban, Tucanoan, Tupian*
		**BBH**	*Arawakan, Cariban, Chibchan, Je-Jabuti, Panoan, Tucanoan, Tupian*
		**BPE**	*Arawakan, Aymaran, Carib, MacroGe, MatacoGuaicuru ,Tupi*
		**BPW**	*Arawakan, Cariban, MacroGe*
		**BPH**	*Arawakan, Cariban, Chibchan, Je-Jabuti, Panoan, Tucanoan, Tupian*
**S America (vs world)**	Do the South American language families form a group?	**[as above]**	
**C America (vs America)**	Do the Central American language families form a group relative to the other American language families?	**MBE**	*Chibchan,Mayan,Oto-Manguean,Uto-Aztecan*
		**MBW**	*Chibchan,Oto-Manguean,Uto-Aztecan*
		**MBH**	*Mayan, Oto-Manguean*
		**MPE**	*Chibchan,Mayan,Oto-Manguean,Uto-Aztecan*
		**MPW**	**[too few language families]**
		**MPH**	*Mayan, Oto-Manguean*
		**BBE**	*Chibchan, Mayan, OtoManguean, UtoAztecan*
		**BBW**	*Chibchan, OtoManguean, UtoAztecan*
		**BBH**	*Mayan, Oto-Manguean*
		**BPE**	*Chibchan, OtoManguean, UtoAztecan*
		**BPW**	*Chibchan, OtoManguean, UtoAztecan*
		**BPH**	*Mayan, Oto-Manguean*

Macro-area	Interpretation	Dataset	Composition
**C America (vs world)**	Do the Central American language families form a group?	**[as above]**	
**N America (vs America)**	Do the North American language families form a group relative to the other American language families?	**MBE**	*Algic, Hokan, Na-Dene, Penutian, Salishan, Wakashan*
		**MBW**	*Algic, Hokan, Na-Dene, Penutian, Salishan*
		**MBH**	*Algic, Athapaskan-Eyak-Tlingit, Cochimi-Yuman, Eskimo-Aleut, Iroquoian, Salishan, Uto-Aztecan*
		**MPE**	*Algic, Eskimo-Aleut, Hokan, Iroquoian, Na-Dene,P enutian, Salishan, Wakashan*
		**MPW**	*Algic, Na-Dene, Penutian*
		**MPH**	*Algic, Athapaskan-Eyak-Tlingit, Cochimi-Yuman, Eskimo-Aleut, Iroquoian, Salishan, Uto-Aztecan*
		**BBE**	*Algic, Hokan, Iroquoian, NaDene, Penutian, Salishan, Tacanan, Wakashan*
		**BBW**	*Algic, Hokan,N aDene,Penutian*
		**BBH**	*Algic, Athapaskan-Eyak-Tlingit, Cochimi-Yuman, Eskimo-Aleut, Iroquoian, Salishan, Uto-Aztecan*
		**BPE**	*Algic, EskimoAleut, Hokan, NaDene, Penutian*
		**BPW**	*Algic, Hokan, NaDene, Penutian, Salishan*
		**BPH**	*Algic, Athapaskan-Eyak-Tlingit, Cochimi-Yuman, Eskimo-Aleut, Iroquoian, Salishan, Uto-Aztecan*
**N America (vs world)**	Do the North American language families form a group?	**[as above]**	
**America + Siberia**	Do the American and Siberian language families form a group?	**MBE**	*Algic, Arawakan, Carib, Chibchan, **Chukotko-Kamchatkan**, Hokan, Macro-Ge, Mayan, Na-Dene, Oto-Manguean, Penutian, Salishan, Tucanoan, Tupi, Uto-Aztecan, Wakashan*
		**MBW**	**[no Siberian language families]**
		**MBH**	*Algic, Arawakan, Athapaskan-Eyak-Tlingit, Cariban, Chibchan, Cochimi-Yuman, Eskimo-Aleut, Iroquoian, Je-Jabuti, Mayan, Otomanguean, Panoan, Salishan, Tucanoan,T upian, Uto-Aztecan, **Chukotko-Kamchatkan***
		**MBH(*)**	*Algic, Arawakan, Athapaskan-Eyak-Tlingit, Cariban, Chibchan, Cochimi-Yuman, Eskimo-Aleut, Iroquoian, Je-Jabuti, Mayan, Otomanguean, Panoan, Salishan, Tucanoan,T upian, Uto-Aztecan, **Chukotko-Kamchatkan**, **Tungusic***
		**MPE**	*Algic, Arawakan, Carib, Chibchan, **Chukotko-Kamchatkan**, Eskimo-Aleut, Hokan,I roquoian, Macro-Ge, Mayan, Na-Dene, Oto-Manguean, Penutian, Salishan, Tucanoan, Tupi, Uto-Aztecan, Wakashan*
		**MPW**	**[no Siberian language families]**
		**MPH**	*Algic, Arawakan, Athapaskan-Eyak-Tlingit, Cariban, Chibchan, Cochimi-Yuman, Eskimo-Aleut, Iroquoian, Je-Jabuti, Mayan, Otomanguean, Panoan, Salishan, Tucanoan,T upian, Uto-Aztecan, **Chukotko-Kamchatkan***
		**MPH(*)**	*Algic, Arawakan, Athapaskan-Eyak-Tlingit, Cariban, Chibchan, Cochimi-Yuman, Eskimo-Aleut, Iroquoian, Je-Jabuti, Mayan, Otomanguean, Panoan, Salishan, Tucanoan,T upian, Uto-Aztecan, **Chukotko-Kamchatkan**, **Tungusic***

* For the **H** classification, two North-Eastern Eurasian ("Siberian") language families can be included in the testing, *Chukotko-Kamchatkan* and *Tungusic*. To also ensure comparability with the other two classifications, we tested two sets, one including only *Chukotko-Kamchatkan* within "Siberia", and another one, marked with a star (*), including both these families within "Siberia".

Macro-area	Interpretation	Dataset	Composition
		BBE	*Algic, Arawakan, Aymaran, Carib, Chibchan,* ***ChukotkoKamchatkan****, Hokan, Iroquoian, MacroGe, MatacoGuaicuru, Mayan, NaDene, OtoManguean, Penutian, Salishan, Tacanan, Tucanoan, Tupi, UtoAztecan, Wakashan, Yanomam,* ***Yukaghir***
		BBW	**[no Siberian language families]**
		BBH	*Algic, Arawakan, Athapaskan-Eyak-Tlingit, Cariban, Chibchan, Cochimi-Yuman, Eskimo-Aleut, Iroquoian, Je-Jabuti, Mayan, Otomanguean, Panoan, Salishan, Tucanoan, Tupian, Uto-Aztecan,* ***Chukotko-Kamchatkan***
		BBH(*)	*Algic, Arawakan, Athapaskan-Eyak-Tlingit, Cariban, Chibchan, Cochimi-Yuman, Eskimo-Aleut, Iroquoian, Je-Jabuti, Mayan, Otomanguean, Panoan, Salishan, Tucanoan, Tupian, Uto-Aztecan,* ***Chukotko-Kamchatkan****,* ***Tungusic***
		BPE	**[no Siberian language families]**
		BPW	**[no Siberian language families]**
		BPH	*Algic, Arawakan, Athapaskan-Eyak-Tlingit, Cariban, Chibchan, Cochimi-Yuman, Eskimo-Aleut, Iroquoian, Je-Jabuti, Mayan, Otomanguean, Panoan, Salishan, Tucanoan, Tupian, Uto-Aztecan,* ***Chukotko-Kamchatkan***
		BPH(*)	*Algic, Arawakan, Athapaskan-Eyak-Tlingit, Cariban, Chibchan, Cochimi-Yuman, Eskimo-Aleut, Iroquoian, Je-Jabuti, Mayan, Otomanguean, Panoan, Salishan, Tucanoan, Tupian, Uto-Aztecan,* ***Chukotko-Kamchatkan****,* ***Tungusic***
**S America + Siberia**	Is Siberia special among world's language families in being close to South America?)	MBE	*Arawakan, Carib,* ***Chukotko-Kamchatkan****, Macro-Ge, Tucanoan, Tupi*
		MBW	**[no Siberian language families]**
		MBH	*Arawakan, Cariban, Chibchan, Je-Jabuti, Panoan, Tucanoan, Tupian,* ***Chukotko-Kamchatkan***
		MBH(*)	*Arawakan, Cariban, Chibchan, Je-Jabuti, Panoan, Tucanoan, Tupian,* ***Chukotko-Kamchatkan****,* ***Tungusic***
		MPE	*Arawakan, Carib,* ***Chukotko-Kamchatkan****, Macro-Ge, Tucanoan, Tupi*
		MPW	**[no Siberian language families]**
		MPH	*Arawakan, Cariban, Chibchan, Je-Jabuti, Panoan, Tucanoan, Tupian,* ***Chukotko-Kamchatkan***
		MPH(*)	*Arawakan, Cariban, Chibchan, Je-Jabuti, Panoan, Tucanoan, Tupian,* ***Chukotko-Kamchatkan****,* ***Tungusic***
		BBE	*Arawakan, Aymaran, Carib,* ***ChukotkoKamchatkan****, MacroGe, MatacoGuaicuru, Tucanoan, Tupi, Yanomam,* ***Yukaghir***
		BBW	**[no Siberian language families]**
		BBH	*Arawakan, Cariban, Chibchan, Je-Jabuti, Panoan, Tucanoan, Tupian,* ***Chukotko-Kamchatkan***
		BBH(*)	*Arawakan, Cariban, Chibchan, Je-Jabuti, Panoan, Tucanoan, Tupian,* ***Chukotko-Kamchatkan****,* ***Tungusic***
		BPE	**[no Siberian language families]**
		BPW	**[no Siberian language families]**
		BPH	*Arawakan, Cariban, Chibchan, Je-Jabuti, Panoan, Tucanoan, Tupian,* ***Chukotko-Kamchatkan***
		BPH(*)	*Arawakan, Cariban, Chibchan, Je-Jabuti, Panoan, Tucanoan, Tupian,* ***Chukotko-Kamchatkan****,* ***Tungusic***
**C America + Siberia**	Is Siberia special among world's language families in being close to Central America?)	MBE	*Chibchan,* ***Chukotko-Kamchatkan****, Mayan, Oto-Manguean, Uto-Aztecan*
		MBW	**[no Siberian language families]**
		MBH	*Mayan, Oto-Manguean,* ***Chukotko-Kamchatkan***

Macro-area	Interpretation	Dataset	Composition
		**MBH(*)**	*Mayan, Oto-Manguean,* ***Chukotko-Kamchatkan****,* ***Tungusic***
		**MPE**	*Chibchan,* ***Chukotko-Kamchatkan****, Mayan, Oto-Manguean, Uto-Aztecan*
		**MPW**	**[no Siberian language families]**
		**MPH**	*Mayan, Oto-Manguean,* ***Chukotko-Kamchatkan***
		**MPH(*)**	*Mayan, Oto-Manguean,* ***Chukotko-Kamchatkan****,* ***Tungusic***
		**BBE**	*Chibchan,* ***ChukotkoKamchatkan****, Mayan, OtoManguean, UtoAztecan,* ***Yukaghir***
		**BBW**	**[no Siberian language families]**
		**BBH**	*Mayan, Oto-Manguean,* ***Chukotko-Kamchatkan***
		**BBH(*)**	*Mayan, Oto-Manguean,* ***Chukotko-Kamchatkan****,* ***Tungusic***
		**BPE**	**[no Siberian language families]**
		**BPW**	**[no Siberian language families]**
		**BPH**	*Mayan, Oto-Manguean,* ***Chukotko-Kamchatkan***
		**BPH(*)**	*Mayan, Oto-Manguean,* ***Chukotko-Kamchatkan****,* ***Tungusic***
**N America + Siberia**	Is Siberia special among world's language families in being close to North America?)	**MBE**	*Algic,* ***Chukotko-Kamchatkan****, Hokan, Na-Dene, Penutian, Salishan, Wakashan*
		**MBW**	**[no Siberian language families]**
		**MBH**	*Algic, Athapaskan-Eyak-Tlingit, Cochimi-Yuman, Eskimo-Aleut, Iroquoian, Salishan, Uto-Aztecan,* ***Chukotko-Kamchatkan***
		**MBH(*)**	*Algic, Athapaskan-Eyak-Tlingit, Cochimi-Yuman, Eskimo-Aleut, Iroquoian, Salishan, Uto-Aztecan,* ***Chukotko-Kamchatkan****,* ***Tungusic***
		**MPE**	*Algic,* ***Chukotko-Kamchatkan****, Eskimo-Aleut, Hokan, Iroquoian, Na-Dene, Penutian, Salishan, Wakashan*
		**MPW**	**[no Siberian language families]**
		**MPH**	*Algic, Athapaskan-Eyak-Tlingit, Cochimi-Yuman, Eskimo-Aleut, Iroquoian, Salishan, Uto-Aztecan,* ***Chukotko-Kamchatkan***
		**MPH(*)**	*Algic, Athapaskan-Eyak-Tlingit, Cochimi-Yuman, Eskimo-Aleut, Iroquoian, Salishan, Uto-Aztecan,* ***Chukotko-Kamchatkan****,* ***Tungusic***
		**BBE**	*Algic,* ***ChukotkoKamchatkan****, Hokan, Iroquoian, NaDene, Penutian, Salishan, Tacanan, Wakashan,* ***Yukaghir***
		**BBW**	**[no Siberian language families]**
		**BBH**	*Algic, Athapaskan-Eyak-Tlingit, Cochimi-Yuman, Eskimo-Aleut, Iroquoian, Salishan, Uto-Aztecan,* ***Chukotko-Kamchatkan***
		**BBH(*)**	*Algic, Athapaskan-Eyak-Tlingit, Cochimi-Yuman, Eskimo-Aleut, Iroquoian, Salishan, Uto-Aztecan,* ***Chukotko-Kamchatkan****,* ***Tungusic***
		**BPE**	**[no Siberian language families]**
		**BPW**	**[no Siberian language families]**
		**BPH**	*Algic, Athapaskan-Eyak-Tlingit, Cochimi-Yuman, Eskimo-Aleut, Iroquoian, Salishan, Uto-Aztecan,* ***Chukotko-Kamchatkan***
		**BPH(*)**	*Algic, Athapaskan-Eyak-Tlingit, Cochimi-Yuman, Eskimo-Aleut, Iroquoian, Salishan, Uto-Aztecan,* ***Chukotko-Kamchatkan****,* ***Tungusic***

Macro-area	Interpretation	Dataset	Composition
**Eurasia**	Do the Eurasian language families from a group?	**MBE**	*Afro-Asiatic, Altaic, Austro-Asiatic, Chukotko-Kamchatkan, Dravidian, Indo-European, North-Caucasian, Sino-Tibetan, Tai-Kadai, Uralic*
		**MBW**	*Afro-Asiatic, Altaic, Austro-Asiatic, Dravidian, Indo-European, Nakh-Daghestanian, Sino-Tibetan, Uralic*
		**MBH**	*Chukotko-Kamchatkan, Dravidian, Indo-European, Mongolic, Nakh-Daghestanian, Tungusic, Turkic, Uralic*
		**MPE**	*Afro-Asiatic, Altaic, Austro-Asiatic, Chukotko-Kamchatkan, Dravidian, Indo-European, North-Caucasian, Sino-Tibetan, Tai-Kadai, Uralic*
		**MPW**	*Afro-Asiatic, Altaic, Austro-Asiatic, Indo-European, Nakh-Daghestanian, Sino-Tibetan, Uralic*
		**MPH**	*Chukotko-Kamchatkan, Dravidian, Indo-European, Mongolic, Nakh-Daghestanian, Tungusic, Turkic, Uralic*
		**BBE**	*AfroAsiatic, Altaic, AustroAsiatic, ChukotkoKamchatkan, Dravidian, IndoEuropean, North Caucasian, SinoTibetan, TaiKadai, Uralic, Yukaghir*
		**BBW**	*AfroAsiatic, Altaic, AustroAsiatic ,Dravidian, IndoEuropean, NakhDaghestanian, SinoTibetan, Uralic*
		**BBH**	*Chukotko-Kamchatkan, Dravidian, Indo-European, Mongolic, Nakh-Daghestanian, Tungusic, Turkic, Uralic*
		**BPE**	*AfroAsiatic, Altaic, AustroAsiatic, Dravidian, IndoEuropean, North Caucasian, SinoTibetan*
		**BPW**	*AfroAsiatic, Altaic, AustroAsiatic, Dravidian, IndoEuropean, NakhDaghestanian, SinoTibetan, Uralic*
		**BPH**	*Chukotko-Kamchatkan, Dravidian, Indo-European, Mongolic, Nakh-Daghestanian, Tungusic, Turkic, Uralic*
**Core Eurasia**	A reduced set of “true” Eurasian language families	**MBE**	*Altaic, Dravidian,I ndo-European, North-Caucasian, Uralic*
		**MBW**	*Altaic, Dravidian, Indo-European, Nakh-Daghestanian ,Uralic*
		**MBH**	*Dravidian, Indo-European, Mongolic, Nakh-Daghestanian, Turkic, Uralic*
		**MPE**	*Altaic, Dravidian, Indo-European, North-Caucasian, Uralic*
		**MPW**	*Altaic, Indo-European, Nakh-Daghestanian, Uralic*
		**MPH**	*Dravidian, Indo-European, Mongolic, Nakh-Daghestanian, Turkic, Uralic*
		**BBE**	*Altaic, Dravidian, IndoEuropean, NakhDaghestanian, Uralic*
		**BBW**	*Altaic, Dravidian, IndoEuropean, NakhDaghestanian, Uralic*
		**BBH**	*Dravidian, Indo-European, Mongolic, Nakh-Daghestanian, Turkic, Uralic*
		**BPE**	*Altaic, Dravidian, IndoEuropean, North Caucasian, Uralic*
		**BPW**	*Altaic, Dravidian, IndoEuropean, NakhDaghestanian ,Uralic*
		**BPH**	*Dravidian, Indo-European, Mongolic, Nakh-Daghestanian, Turkic, Uralic*
**Nostratic v1**	A core set of language families across various Nostratic proposals.	**MBE**	*Altaic, Indo-European, Uralic*
		**MBW**	*Altaic, Indo-European, Uralic*
		**MBH**	*Indo-European, Uralic, Mongolic, Turkic*
		**MPE**	*Altaic,Indo-European, Uralic*
		**MPW**	*Altaic, Indo-European, Uralic*
		**MPH**	*Indo-European, Uralic, Mongolic, Turkic*

Macro-area	Interpretation	Dataset	Composition
		**BBE**	*Altaic, IndoEuropean, Uralic*
		**BBW**	*Altaic, IndoEuropean, Uralic*
		**BBH**	*Indo-European, Uralic, Mongolic, Turkic*
		**BPE**	*Altaic ,IndoEuropean, Uralic*
		**BPW**	*Altaic, IndoEuropean, Uralic*
		**BPH**	*Indo-European, Uralic, Mongolic, Turkic*
**Nostratic v2**	A variant definition of Nostratic.	**MBE**	*Afro-Asiatic, Dravidian, Indo-European, Uralic*
		**MBW**	*Afro-Asiatic, Dravidian, Indo-European, Uralic*
		**MBH**	*Indo-European, Uralic, Dravidian, Afro-Asiatic*
		**MPE**	*Afro-Asiatic, Dravidian, Indo-European, Uralic*
		**MPW**	*Afro-Asiatic, Indo-European, Uralic*
		**MPH**	*Indo-European, Uralic, Dravidian, Afro-Asiatic*
		**BBE**	*AfroAsiatic, Dravidian, IndoEuropean, Uralic*
		**BBW**	*AfroAsiatic, Dravidian, IndoEuropean, Uralic*
		**BBH**	*Indo-European, Uralic, Dravidian, Afro-Asiatic*
		**BPE**	*AfroAsiatic, Dravidian, IndoEuropean, Uralic*
		**BPW**	*AfroAsiatic, Dravidian, IndoEuropean, Uralic*
		**BPH**	*Indo-European, Uralic, Dravidian, Afro-Asiatic*
**PNG**	Do the Papuan language families form a group?	**MBE**	*Sepik, Trans-New-Guinea, West-Papuan*
		**MBW**	**[not enough Papuan language families]**
		**MBH**	*North Halmahera, Sepik, Trans-New Guinea*
		**MPE**	*Sepik, Trans-New-Guinea*
		**MPW**	**[not enough Papuan language families]**
		**MPH**	*North Halmahera, Sepik, Trans-New Guinea*
		**BBE**	*Sepik, TransNew Guinea, West Papuan*
		**BBW**	**[not enough Papuan language families]**
		**BBH**	*North Halmahera, Sepik, Trans-New Guinea*
		**BPE**	**[not enough Papuan language families]**
		**BPW**	**[not enough Papuan language families]**
		**BBH**	*North Halmahera, Sepik, Trans-New Guinea*

Macro-area	Interpretation	Dataset	Composition
**PNG + Australia**	Do the Papuan and Australian language families form a group?	**MBE**	*Australian,Sepik,Trans-New-Guinea,West-Papuan*
		**MBW**	*Australian, Sepik*
		**MBH**	*Gunwinyguan, North Halmahera, Pama-Nyungan, Sepik, Trans-New Guinea*
		**MPE**	*Australian,Sepik,Trans-New-Guinea*
		**MPW**	*Australian, Trans-New Guinea*
		**MPH**	*Gunwinyguan, North Halmahera, Pama-Nyungan, Sepik, Trans-New Guinea*
		**BBE**	*Australian,Sepik,TransNew Guinea,West Papuan*
		**BBW**	*Australian, Trans-New Guinea*
		**BBH**	*Gunwinyguan, North Halmahera, Pama-Nyungan, Sepik, Trans-New Guinea*
		**BPE**	*Australian, Tran-sNew Guinea*
		**BPW**	*Australian, Sepik*
		**BPH**	*Gunwinyguan, North Halmahera, Pama-Nyungan, Sepik, Trans-New Guinea*
**South-East Asia and Oceania**	A set of languages of SE Asia and Austronesian.	**MBE**	*Austro-Asiatic,Austronesian,Sino-Tibetan,Tai-Kadai*
		**MBW**	*Austro-Asiatic,Austronesian,Sino-Tibetan*
		**MBH**	*Austro-Asiatic, Austronesian, Sino-Tibetan, Tai-Kadai*
		**MPE**	*Austro-Asiatic,Austronesian,Sino-Tibetan,Tai-Kadai*
		**MPW**	*Austro-Asiatic,Austronesian,Sino-Tibetan*
		**MPH**	*Austro-Asiatic, Austronesian, Sino-Tibetan, Tai-Kadai*
		**BBE**	*Austro-Asiatic,Austronesian,SinoTibetan,TaiKadai*
		**BBW**	*Austro-Asiatic, Austronesian*
		**BBH**	*Austro-Asiatic, Austronesian, Sino-Tibetan, Tai-Kadai*
		**BPE**	*AustroAsiatic,Austronesian,SinoTibetan*
		**BPW**	*AustroAsiatic,Austronesian,SinoTibetan*
		**BPH**	*Austro-Asiatic, Austronesian, Sino-Tibetan, Tai-Kadai*
**Austro-Tai**	Do Austronesian and Tai-Kadai families form a group?	**MBE**	*Austro-Asiatic, Tai-Kadai*
		**MBW**	**[not enough language families]**
		**MBH**	*Austro-Asiatic, Tai-Kadai*
		**MPE**	*Austro-Asiatic, Tai-Kadai*
		**MPW**	**[not enough language families]**
		**MPH**	*Austro-Asiatic, Tai-Kadai*

Macro-area	Interpretation	Dataset	Composition
		**BBE**	*Austro-Asiatic, Tai-Kadai*
		**BBW**	**[not enough language families]**
		**BBH**	*Austro-Asiatic, Tai-Kadai*
		**BPE**	**[not enough language families]**
		**BPW**	**[not enough language families]**
		**BPH**	*Austro-Asiatic, Tai-Kadai*
**Australia**[2]	Do the Australian families form a group?	**MBE**	**[N/A (see footnote [2])]**
		**MBW**	**[N/A (see footnote [2])]**
		**MBH**	*Gunwinyguan, Pama-Nyungan*
		**MPE**	**[N/A (see footnote [2])]**
		**MPW**	**[N/A (see footnote [2])]**
		**MPH**	*Gunwinyguan, Pama-Nyungan*
		**BBE**	**[N/A (see footnote [2])]**
		**BBW**	**[N/A (see footnote [2])]**
		**BBH**	*Gunwinyguan, Pama-Nyungan*
		**BPE**	**[N/A (see footnote [2])]**
		**BPW**	**[N/A (see footnote [2])]**
		**BPH**	*Gunwinyguan, Pama-Nyungan*

Table S15*:The sets of language families considered with their interpretation and composition for each dataset.*

2 Only classification **H** proposes more than a single language family for **Australia** (the other two have a single controversial construct "*Australian*"), making the testing of this hypothesis possible only for the four datasets using **H** (**MBH**, **MPH**, **BBH** and **BPH**).

Macro-area	Geo	*P*-values for each dataset												Paired *t*-test	Methods for combining *p*-values						
		BBE	BBW	BBH	BPE	BPW	BPH	MBE	MBW	MBH	MPE	MPW	MPH		F	Z	H		S	M	
																	p	*r*		*p*	*r*
**Africa**	No	0.51	0.40	0.53	*0.008*	*0.012*	0.22	0.72	0.13	0.42	0.57	-	0.28	t_{10}= **-3.90**<br>p= 0.0029	*0.029*	*0.020*	**0.074**	0.06	No	*0.029*	0.00
	Yes	0.87	0.71	0.55	0.052	*0.032*	0.19	0.88	0.36	0.60	0.74	-	0.44		0.36	**0.39**	**0.39**	-0.05	No	0.36	0.00
**America**	No	*$< 10^{-4}$*	*$< 10^{-4}$*	*0.0059*	*0.016*	*0.004*	*0.010*	*0.0076*	*0.036*	*0.034*	*0.001*	0.079	*0.03*	t_{11}= **-2.64**<br>p= 0.023	*0.00†*	*0.00*	***0.0003***	0.45	*Yes*	*$1.89 \bullet 10^{-15}$*	0.00
	Yes	0.30	*0.0002*	*0.0018*	0.37	*0.022*	*0.0023*	0.11	0.32	0.14	*0.03*	0.052	0.11		***$2.69 \bullet 10^{-8}$***	*$6.53 \bullet 10^{-9}$*	*$1.2 \bullet 10^{-14}$*	-0.09	*Yes*	***$2.69 \bullet 10^{-8}$***	0.00
**S America**<br>(vs world)	No	*0.044*	*0.0018*	*0.038*	0.30	0.28	0.12	*0.0098*	*0.032*	*0.022*	*0.0037*	-	*0.026*	t_{10}= -1.95<br>p= 0.079	*$5.05 \bullet 10^{-8}$*	*$1.98 \bullet 10^{-9}$*	***0.0054***	0.42	*Yes*	*$5.05 \bullet 10^{-8}$*	0.00
	Yes	0.63	*0.0087*	*0.032*	0.75	0.38	0.10	*0.031*	0.12	0.066	*0.0125*	-	0.0605		*$9.89 \bullet 10^{-5}$*	*$3.47 \bullet 10^{-5}$*	***0.00018***	-0.02	No	*$9.89 \bullet 10^{-5}$*	0.00
**S America**<br>(vs America)	No	0.53	0.077	0.24	0.73	0.64	0.54	*0.032*	0.051	0.073	*0.032*	-	0.12	t_{10}= ***+2.71***<br>p= 0.022	*0.0075*	*0.0032*	***0.049***	0.14	No	*0.0075*	0.00
	Yes	0.13	0.16	0.082	0.20	0.49	*0.04*	*0.016*	*0.049*	*0.0052*	*$7.6 \bullet 10^{-5}$*	-	*$6.8 \bullet 10^{-5}$*		***$1.20 \bullet 10^{-9}$***	*$7.07 \bullet 10^{-10}$*	*$8.1 \bullet 10^{-17}$*	-0.10	*Yes*	***$1.20 \bullet 10^{-9}$***	0.00
**C America**<br>(vs world)	No	0.25	0.11	0.05	0.41	0.37	0.31	0.42	0.48	0.84	0.71	-	0.94	t_{10}= **-4.15**<br>p= 0.002	0.37	0.29	**0.38**	0.17	No	**0.38**	0.29
	Yes	0.65	0.21	0.12	0.65	0.47	0.31	0.66	0.76	0.91	0.88	-	0.96		0.86	0.86	0.73	0.16	No	**0.90**	0.58
**C America**<br>(vs America)	No	0.78	0.70	0.09	0.60	0.80	0.47	0.82	0.7	0.92	0.97	-	0.94	t_{10}= *+2.07*<br>p= 0.066	0.98	**0.99**	0.88	0.23	No	0.73	0.58
	Yes	0.62	0.78	0.11	0.54	0.70	0.35	0.80	0.70	0.95	0.79	-	0.96		**0.96**	0.95	0.79	0.30	No	0.67	0.64
**N America**<br>(vs world)	No	*0.012*	*0.029*	0.13	*0.0054*	*0.033*	0.085	*0.036*	*0.012*	*0.023*	*0.015*	0.17	*0.020*	t_{11}= **-2.25**<br>p= 0.046	*$9.05 \bullet 10^{-9}$*	*$6.19 \bullet 10^{-11}$*	***0.018***	0.77	*Yes*	*$9.05 \bullet 10^{-9}$*	0.00
	Yes	0.23	0.071	0.088	0.057	0.065	*0.046*	0.12	0.071	0.059	0.069	0.18	*0.038*		*$4.59 \bullet 10^{-5}$*	*$6.83 \bullet 10^{-7}$*	**0.072**	0.91	No	0.06	0.75
**N America**<br>(vs America)	No	0.15	0.39	0.67	0.05	0.21	0.44	0.20	*0.012*	0.10	0.23	0.40	0.11	t_{11}= ***+3.05***<br>p= 0.011	*0.0093*	*0.0017*	**0.12**	0.47	No	*0.0093*	0.00
	Yes	*0.037*	0.55	0.53	*0.0038*	*0.034*	0.084	0.087	*0.0006*	*0.021*	*0.0001*	0.28	*0.0015*		***$4.61 \bullet 10^{-10}$***	*$4.09 \bullet 10^{-10}$*	*$9.0 \bullet 10^{-17}$*	-0.09	*Yes*	***$4.61 \bullet 10^{-10}$***	0.00
**America +**<br>**Siberia**	No	*0.0001*	-	*0.0026*<br>*0.0061‡*	-	-	*0.0070*<br>*0.0073*	*0.0035*	-	*0.021*<br>*0.031*	*0.0001*	-	*0.0056*<br>*0.0039*	t_6= -1.808<br>p= 0.12	*$8.72 \bullet 10^{-13}$*<br>*$1.96 \bullet 10^{-12}$*	*$2.29 \bullet 10^{-14}$*<br>*$6.30 \bullet 10^{-14}$*	***0.00022***<br>***0.00017***	0.59<br>0.53	*Yes*<br>*Yes*	*$8.72 \bullet 10^{-13}$*<br>*$1.96 \bullet 10^{-12}$*	0.00<br>0.00
	Yes	0.17	-	*0.0009*<br>*0.0017*	-	-	*0.0015*<br>*0.0014*	0.059	-	0.074<br>0.10	*0.0049*	-	*0.023*<br>*0.014*	t_6= -1.78<br>p= 0.12	*$1.47 \bullet 10^{-7}$*<br>*$1.81 \bullet 10^{-7}$*	*$1.69 \bullet 10^{-8}$*<br>*$2.11 \bullet 10^{-8}$*	***0.00096***<br>***0.001***	0.33<br>0.33	*Yes*<br>*Yes*	*$1.47 \bullet 10^{-7}$*<br>*$1.81 \bullet 10^{-7}$*	0.00<br>0.00
**S America +**<br>**Siberia**	No	0.11	-	*0.023*<br>*0.044*	-	-	0.07<br>0.095	0.34	-	*0.018*<br>*0.031*	0.11	-	*0.0038*<br>*0.0040*	t_6= *+0.99*<br>p= 0.36	*$8.24 \bullet 10^{-5}$*<br>*0.00025*	*$1.29 \bullet 10^{-5}$*<br>*$3.93 \bullet 10^{-5}$*	***0.02***<br>***0.028***	0.47<br>0.53	*Yes*<br>*Yes*	*$8.24 \bullet 10^{-5}$*<br>*0.00025*	0.00<br>0.00
	Yes	0.14	-	*0.013*<br>*0.025*	-	-	*0.043*<br>0.054	0.316	-	*0.032*<br>0.057	*0.042*	-	*0.0042*<br>*0.0043*	t_6= *+0.99*<br>p= 0.36	*$3.46 \bullet 10^{-5}$*<br>*0.0001*	*$4.59 \bullet 10^{-6}$*<br>*$1.40 \bullet 10^{-5}$*	***0.014***<br>***0.023***	0.50<br>0.55	*Yes*<br>*Yes*	*$3.46 \bullet 10^{-5}$*<br>*0.0001*	0.00<br>0.00
**C America +**<br>**Siberia**	No	0.42	-	0.26<br>0.37	-	-	0.45<br>0.46	0.29	-	0.60<br>0.72	0.10	-	0.59<br>0.45	t_6= -1.51<br>p= 0.18	**0.37**<br>**0.41**	0.19<br>0.22	0.35<br>0.37	0.71<br>0.69	No<br>No	0.35<br>0.37	0.66<br>0.67

† A combined *p*-value of exactly 0.00 is an artifact of combining very small randomization *p*-values (smaller than the lower limit given by the number of randomizations used here, 10^{-4}) and represents in fact an extremely small (but still greater than 0) *p*-value.

‡ For tests involving **Siberia** (N-E Eurasia), the **first** entry in the **H** cells represents *Chukotko-Kamchatkan* & *Tungusic* while the **second** entry represents *Chukotko-Kamchatkan* only; this extends to the combined *p*-value cells.

Macro-area	Geo	*P*-values for each dataset												Paired *t*-test	Methods for combining *p*-values						
		BBE	BBW	BBH	BPE	BPW	BPH	MBE	MBW	MBH	MPE	MPW	MPH		F	Z	H		S	M	
																	p	*r*		*p*	*r*
	Yes	0.52	-	0.22<br>0.34	-	-	0.43<br>0.44	0.28	-	0.71<br>0.81	0.11	-	0.70<br>0.56	t_6= -1.44<br>*p*= 0.20	**0.42**<br>**0.47**	0.27<br>0.31	0.38<br>0.41	0.55<br>0.56	No<br>No	0.38<br>0.40	0.58<br>0.62
**N America + Siberia**	No	0.22	-	0.083<br>0.13	-	-	0.075<br>0.052	0.078	-	*0.033*<br>*0.030*	*< 10^{-4}*	-	*0.02*<br>*0.0034*	t_6= -1.15<br>*p*= 0.30	*0.00*<br>*0.00*	*0.00*<br>*0.00*	***0.00039***<br>***$3.31 \cdot 10^{-6}$***	0.12<br>-0.02	*Yes*<br>*Yes*	*$3.72 \cdot 10^{-6}$*<br>*$8.64 \cdot 10^{-7}$*	0.00<br>0.00
	Yes	0.54	-	*0.049*<br>0.096	-	-	*0.039*<br>*0.029*	0.11	-	0.073<br>0.080	*0.019*	-	*0.043*<br>*0.0063*	t_6= -1.17<br>*p*= 0.28	*0.00062*<br>*0.00022*	*0.00012*<br>*$5.18 \cdot 10^{-5}$*	***0.034***<br>***0.013***	0.49<br>0.31	No<br>*Yes*	*0.00062*<br>*0.00022*	0.00<br>0.00
**Core Eurasia**	No	0.48	0.64	0.42	0.62	0.72	0.77	0.178	*0.0083*	*$9.9 \cdot 10^{-5}$*	0.24	0.056	*0.0026*	t_{11}= **-3.09**	*0.00011*	***0.0013***	*$2.84 \cdot 10^{-5}$*	-0.09	*Yes*	*0.00011*	0.00
	Yes	0.89	0.91	0.45	0.90	0.84	0.77	0.34	*0.039*	*0.0003*	0.44	*0.047*	*0.0052*	*p*= 0.01	*0.0039*	**0.094**	*0.039*	-0.09	*Yes*	*0.0039*	0.00
**Nostratic v1**	No	0.53	0.59	0.64	0.77	0.52	0.87	0.30	*0.035*	*0.002*	0.15	0.066	*0.0025*	t_{11}= **-3.45**	*0.0025*	***0.011***	*0.001*	-0.09	*Yes*	*0.0025*	0.00
	Yes	0.79	0.83	0.67	0.92	0.61	0.87	0.46	0.10	*0.004*	0.25	0.07	*0.004*	*p*= 0.0054	*0.025*	**0.13**	0.069	-0.09	*Yes*	*0.025*	0.00
**Nostratic v2**	No	0.76	0.75	0.74	0.84	0.67	0.82	0.36	0.07	0.093	0.22	*0.037*	0.13	t_{11}= **-5.08**	0.21	**0.24**	0.17	-0.09	No	0.21	0.00
	Yes	0.95	0.91	0.79	0.96	0.76	0.83	0.50	0.16	0.14	0.33	*0.033*	0.17	*p*= 0.0004	0.50	0.71	**0.77**	-0.09	No	0.50	0.00
**Eurasia**	No	0.73	0.93	0.43	0.62	0.67	0.76	0.41	*0.026*	*0.0002*	0.38	0.19	*0.0029*	t_{11}= **-2.82**	*0.0027*	***0.036***	*0.0077*	-0.09	*Yes*	*0.0027*	0.00
	Yes	0.99	0.99	0.44	0.93	0.82	0.72	0.71	0.17	*0.001*	0.70	0.12	*0.0043*	*p*= 0.017	*0.045*	0.65	**0.70**	-0.09	*Yes*	*0.0451*	0.00
**PNG**	No	0.64	-	0.72	-	-	0.82	*0.017*	-	*0.012*	0.19	-	0.12	t_6= -2.14	*0.023*	***0.042***	*0.0056*	-0.17	No	*0.023*	0.00
	Yes	0.93	-	0.76	-	-	0.83	*0.038*	-	*0.027*	0.292	-	0.19	*p*= 0.076	0.11	**0.22**	0.13	-0.17	No	0.11	0.00
**PNG + Australia**	No	0.88	0.84	0.62	0.58	0.82	0.77	0.26	0.65	0.10	0.67	0.78	0.17	t_{11}= **-5.82**	**0.87**	0.81	0.65	0.37	No	0.59	0.55
	Yes	0.99	0.94	0.68	0.71	0.88	0.77	0.50	0.81	0.20	0.83	0.82	0.30	*p*= 0.0001	**0.99**	**0.99**	0.89	0.23	No	0.69	0.79
**Austro-Tai**	No	0.20	-	0.078	-	-	0.27	0.08	-	0.24	*0.031*	-	*0.022*	t_6= **-2.82**	*0.0025*	*0.00041*	**0.070**	0.69	No	*0.0027*	0.00
	Yes	0.36	-	0.14	-	-	0.27	0.15	-	0.32	*0.049*	-	*0.037*	*p*= 0.030	*0.018*	*0.0041*	**0.12**	0.68	No	*0.063*	0.22
**South-East Asia and Oceania**	No	0.39	0.58	0.38	0.68	0.34	0.19	0.55	0.39	0.59	0.29	0.34	0.15	t_{11}= **-4.47**	**0.48**	0.18	0.39	0.79	No	0.39	0.82
	Yes	0.74	0.75	0.39	0.84	0.42	0.19	0.75	0.59	0.73	0.46	0.38	0.20	*p*= 0.0009	**0.83**	0.64	0.55	0.61	No	0.52	0.77
**Australia**	No	-	-	0.65	-	-	0.45	-	-	0.22	-	-	0.28	t_3= -0.62	**0.42**	0.29	0.38	0.72	No	0.38	0.79
	Yes	-	-	0.61	-	-	0.42	-	-	0.30	-	-	0.36	*p*= 0.58	**0.51**	0.34	0.42	0.88	No	0.41	0.92

***Table S16**: Statistical robustness of sets of language families. Actual p-values for each of the datasets and the combined p-values using the five methods (**F**isher, **Z**-transform, **H**artung, **S**imes and **M**akambi) applied to all datasets for raw (**Geo** is "No") and geography-corrected (**Geo** is "Yes") stability distances. Also showing the paired t-tests between the raw and geography-corrected p-values (**bold**=significant t-test, italic=positive t-test). For **S** we show if H_0 was rejected ("Yes" or "No") for α=0.05. For **H** and **M** the estimated inter-datasets correlations are also shown. The most **conservative** combined p-value among the methods is in bold. Significant p-values at α=0.05 are in italic. See **Table S15** for the actual composition of the sets of families.*

Method	R implementation

Fisher [30]

```
library(survcomp)
combine.test( ..., method="fisher" )
```

Z-transform [31]

```
library(survcomp)
combine.test( ..., method="z.transform" )
```

Hartung [52]

```
# Hartung 1999: assumes constant correlation across tests:
hartung.1999 <- function( pi, lowest.p=10^-16 )
{
  # The number of tests:
  N <- length(pi);

  # Truncate the smallest p-values to the lowest possible (to avoid qnorm(0) basically):
  pi <- pmax( pi, rep(lowest.p,N) );

  # Compute the probits t_{i} = phi^{-1}(p_{1}), where phi(.) = standard normal cumulative distribution function (probit) =
qnorm(.) in R:
  ti <- qnorm( pi );

  # The mean of t_{i}:
  t.mean <- mean(ti, na.rm=TRUE );

  # rho_hat:
  rho.hat <- 1 - (1/(N-1)) * sum((ti - t.mean)^2);

  # the rho estimate:
  rho.star.hat <- max( -(1/(N-1)), rho.hat );

  # correction factor kappa:
  kappa <- 0.1 * (1 + 1/(N-1) - rho.star.hat);

  # the modified inverse normal test statistics:
  Zm <- sum(ti) / sqrt(N + N*(N-1)*(rho.star.hat + kappa*sqrt(2/(N+1))*(1-rho.star.hat)));

  # Zm should be distributed as N(0,1):
  p.val <- pnorm(Zm);

  # Return value:
  list( "p.value"=p.val, "estim.corr"=rho.star.hat );
}
```

Makambi [53]

```
# Makambi 2003: extension of Fisher's method for positively correlated dependent cases and assumed homogeneity of
correlations
# the weights must sum up to 1 and could reflect sample size for the tests (by default they are all equal to 1/N):
makambi.2003 <- function( pi, alpha=0.05, weights=rep(1/length(pi),length(pi)), lowest.p=10^-16 )
{
  # The number of tests:
```

Method	R implementation
	<pre> N <- length(pi);<br><br># Truncate the smallest p-values to the lowest possible (to avoid log(0) basically):<br>pi <- pmax(pi, rep(lowest.p,N));<br><br># Estimate the positive homogenous correlation among tests, rho.hat:<br><br># compute si:<br>si <- -2*log(pi);<br># and their average s.bar:<br>s.bar <- mean(si,na.rm=TRUE);<br><br># the quadratic form qt:<br>qt <- sum((si-s.bar)^2) / (N-1);<br>if(4*qt/3 < 10.028)<br>{<br># rho.hat:<br>rho.hat <- -2.167 + sqrt(10.028 - 4*qt/3);<br><br># the estimated positive homogenous correlation among tests rho.hat.star:<br>rho.hat.star <- max(rho.hat, 0);<br>}<br>else<br>{<br>rho.hat.star <- 0;<br>}<br><br># Compute MF, the weighted Fisher's statistic:<br>MF <- sum(-2*weights*log(pi));<br><br># The estimated variance of MF:<br>var.MF <- 4*sum(weights^2) + sum(as.numeric(sapply(1:N, function(i){ sapply(1:N, function(j){ ifelse(i != j,<br>weights[i]*weights[j]*(3.25*rho.hat.star + 0.75*rho.hat.star^2), 0) }) })), na.rm=TRUE);<br><br># And the estimated degrees of freedom of the chi-square test, nu.hat:<br>nu.hat <- 8/var.MF;<br><br># Reject the null?<br>reject.null <- (MF > 2*qchisq(1-alpha, nu.hat)/nu.hat);<br><br># and the associated p-value:<br>p.value <- 1 - pchisq(nu.hat * MF / 2, nu.hat);<br><br>## Print the results:<br>#cat("MF=", MF, " distributed as 2*chisq(df=", nu.hat, ")/", nu.hat, " rejects the null ", reject.null, " with p=",<br>p.value, "\n", sep="");</pre>

Method	R implementation
	` # Return value:`<br>` list( "p.value"=p.value, "estim.corr"=rho.hat.star );`<br>`}`
**Simes** [54]	`# Simes 1986: robust to dependence but the resulting p-value cannot be smaller than the minimum p-value inputted:`<br>`simes.1986 <- function( pi, alpha=0.05 )`<br>`{`<br>` # The number of tests:`<br>` N <- length(pi);`<br><br>` # Sort the p-values:`<br>` pi.sorted <- sort( pi );`<br><br>` # Check the rejection criterion:`<br>` pi.reject <- (pi.sorted < (1:N)*alpha/N);`<br><br>` # If just one is true, then reject the null:`<br>` (sum( pi.reject ) > 0);`<br>`}`

Table S17*: The* `R` *[47] code implementing the methods for combining p-values used here.* ***Fisher*** *[30] and* ***Z-Transform*** *[31] are implemented by function* `combine.test` *in library* `survcomp`*.* ***Hartung*** *[52],* ***Makambi*** *[53] and* ***Simes*** *[54] were implemented by the first author in* `R` *from descriptions in the primary literature and are released under GLPv3.*

Family	MBE		MPE		MBH		MPH		BBE		BPE		BBH		BPH	
	r	*ρ*	*r*	*ρ*	*r*	*ρ*	*r*	*ρ*	*r*	*ρ*	*r*	*ρ*	*r*	*ρ*	*r*	*ρ*
**All**	**0.78**	**0.72**	**0.65**	**0.62**	**0.80**	**0.78**	**0.74**	**0.67**	**0.74**	**0.70**	**0.79**	**0.70**	**0.77**	**0.75**	**0.77**	**0.76**
**Afro-Asiatic**	0.53	0.54	0.63	0.66	0.50	0.50	0.55	0.55	0.31	0.19	0.23	-0.03	0.05	-0.07	0.41	0.38
**Algic**	0.34	0.35	0.03	0.03	0.52	0.50	0.40	0.40	0.52	0.55	0.52	0.52	0.79	0.80	0.77	0.80
**Altaic**	0.45	0.48	0.40	0.44	-	-	-	-	0.42	0.56	0.43	0.64	-	-	-	-
**Arawakan**	0.64	0.68	0.59	0.60	0.77	0.79	0.79	0.81	-	-	-	-	0.41	0.32	0.30	0.35
**Athapaskan-Eyak-Tlingit**	-	-	-	-	0.30	0.31	0.49	0.48	-	-	-	-	0.66	0.70	0.70	0.69
**Atlantic-Congo**	-	-	-	-	0.58	0.60	0.40	0.44	-	-	-	-	0.53	0.51	0.64	0.67
**Australian**	0.58	0.59	0.67	0.67	-	-	-	-	0.22	0.24	0.28	0.32	-	-	-	-
**Austro-Asiatic**	0.58	0.59	0.55	0.57	0.73	0.72	0.58	0.57	-0.06	0.11	0.08	0.09	0.26	0.27	0.24	0.28
**Austronesian**	0.86	0.88	0.81	0.81	0.78	0.78	0.81	0.82	0.84	0.87	0.83	0.85	0.74	0.77	0.73	0.79
**Cariban**	-0.06	0.08	-0.09	0.12	0.24	0.26	0.09	0.17	0.76	0.75	-	-	0.75	0.82	0.62	0.63
**Central Sudanic**	-	-	-	-	0.20	0.24	0.27	0.27	-	-	-	-	0.62	0.62	0.61	0.59
**Chibchan**	0.56	0.58	0.47	0.50	0.55	0.58	0.59	0.61	-	-	-	-	0.76	0.73	0.78	0.62
**Chukotko-Kamchatkan**	-0.06	-0.02	-0.29	-0.29	-0.25	-0.22	-0.39	-0.40	0.47	0.48						
**Cochimi-Yuman**	-	-	-	-	-0.18	-0.06	-0.16	-0.04	-	-	-	-	-	-	-	-
**Dravidian**	0.09	0.16	0.41	0.45	0.41	0.44	0.38	0.42	-0.13	-0.29	0.70	0.71	0.58	0.49	0.76	0.71
**Eskimo-Aleut**	-	-	0.27	0.28	-0.04	0.02	0.00†	0.05	-	-	0.16	0.13	0.50	0.50	0.50	0.50
**Gunwinyguan**	-	-	-	-	0.32	0.33	0.40	0.41	-	-	-	-	-	-	-	-
**Hokan**	0.51	0.54	0.32	0.34	-	-	-	-	-0.12	-0.15	-0.24	-0.27	-	-	-	-
**Indo-European**	0.70	0.72	0.71	0.72	0.74	0.73	0.69	0.70	0.42	0.47	0.80	0.73	0.59	0.69	0.63	0.71
**Iroquoian**	-	-	0.51	0.52	0.57	0.60	0.62	0.64	0.68	0.72	-	-	0.75	0.73	0.80	0.78
**Je-Jabuti**	-	-	-	-	-0.29	-0.15	-0.36	-0.18	-	-	-	-	0.48	0.49	0.40	0.41
**Khoisan**	0.22	0.23	0.33	0.32	-	-	-	-	0.40	0.41	0.03	0.02	-	-	-	-
**Macro-Ge**	0.10	0.18	-0.08	0.00†	-	-	-	-	0.71	0.75	0.55	0.57	-	-	-	-
**Mande**	-	-	-	-	0.04	0.17	-0.03	0.12	-	-	-	-	0.58	0.56	0.36	0.40
**Mayan**	0.22	0.26	-0.03	0.06	0.13	0.15	-0.07	-0.02	0.53	0.55	-	-	0.66	0.66	0.74	0.77
**Mongolic**	-	-	-	-	0.44	0.54	0.37	0.48	-	-	-	-	0.49	0.52	0.52	0.55

Family	MBE		MPE		MBH		MPH		BBE		BPE		BBH		BPH	
	r	*ρ*	*r*	*ρ*	*r*	*ρ*	*r*	*ρ*	*r*	*ρ*	*r*	*ρ*	*r*	*ρ*	*r*	*ρ*
**Muskogean**	-	-	-	-	-	-	-0.32	-0.09	-	-	-	-	-	-	-	-
**Na-Dene**	0.37	0.41	0.52	0.53	-	-	-	-	0.72	0.76	0.66	0.74	-	-	-	-
**Nakh-Daghestanian**	-	-	-	-	-0.30	-0.01	-0.27	0.06	-	-	-	-	0.36	0.33	0.46	0.46
**Niger-Congo**	0.59	0.59	0.50	0.45	-	-	-	-	0.47	0.44	0.72	0.67	-	-	-	-
**Nilo-Saharan**	0.65	0.67	0.61	0.63	-	-	-	-	0.75	0.77	0.81	0.85	-	-	-	-
**Nilotic**	-	-	-	-	0.33	0.37	0.25	0.31	-	-	-	-	0.29	0.31	0.38	0.44
**North Caucasian**	0.00	0.22	0.08	0.17	-	-	-	-	-0.02	-0.01	-0.44	-0.42	-	-	-	-
**North Halmahera**	-	-	-	-	-0.09	0.03	-0.28	-0.07	-	-	-	-	0.71	0.79	0.72	0.79
**Oto-Manguean**	0.56	0.57	0.25	0.30	0.20	0.27	0.06	0.22	0.64	0.66	0.71	0.75	0.53	0.58	0.33	0.37
**Pama-Nyungan**	-	-	-	-	0.63	0.65	0.60	0.61	-	-	-	-	0.66	0.56	0.60	0.45
**Panoan**	-	-	-	-	0.09	0.10	-0.09	-0.04	-	-	-	-	0.47	0.46	0.42	0.42
**Penutian**	0.67	0.70	0.71	0.72	-	-	-	-	0.47	-0.03	-0.18	-0.29	-	-	-	-
**Salishan**	-0.34	-0.13	-0.20	-0.02	-0.25	0.01	-0.21	-0.06	0.25	0.26	-	-	0.39	0.36	0.60	0.57
**Sepik**	0.26	0.27	-0.01	0.06	0.29	0.27	0.22	0.25	-0.20	-0.07	-	-	0.40	0.40	0.19	0.20
**Sino-Tibetan**	0.53	0.57	0.40	0.46	0.67	0.69	0.68	0.69	0.55	0.52	0.51	0.49	0.73	0.69	0.53	0.47
**Tacanan**	-	-	-	-	-	-	-	-	0.31	0.30	-	-	-	-	-	-
**Tai-Kadai**	-0.30	-0.13	-0.38	-0.12	-0.02	0.15	-0.29	-0.03	0.36	0.36	-	-	0.08	0.10	0.32	0.32
**Trans-New Guinea**	0.66	0.68	0.66	0.68	0.73	0.74	0.78	0.79	0.48	0.44	0.15	0.26	0.44	0.44	0.46	0.47
**Tucanoan**	-0.20	-0.09	0.12	0.18	0.20	0.23	0.38	0.41	0.64	0.68	-	-	0.67	0.69	0.65	0.68
**Tungusic**	-	-	-	-	0.05	0.14	0.11	0.22	-	-	-	-	0.50	0.45	0.44	0.41
**Tupian**	0.28	0.32	-0.02	0.09	0.36	0.35	-0.18	-0.16	-	-	-	-	0.31	0.31	0.30	0.30
**Turkic**	-	-	-	-	0.57	0.59	0.26	0.42	-	-	-	-	0.69	0.64	0.70	0.65
**Uralic**	0.50	0.50	0.19	0.23	0.63	0.66	0.63	0.65	0.00[†]	-0.04	-0.01	-0.01	0.36	0.35	0.07	0.05
**Uto-Aztecan**	0.33	0.38	0.25	0.28	0.55	0.58	0.57	0.58	0.62	0.64	0.51	0.51	0.60	0.62	0.61	0.58
**Wakashan**	-0.39	-0.32	-0.66	-0.54	-	-	-	-	-	-	-	-	-	-	-	-
**West Papuan**	-0.08	-0.02	-	-	-	-	-	-	-	-	-	-	-	-	-	-

Table S18*: The correlations between branch length and number of nodes for all language families amalgamated (first line) and each family separately for each of the 8*

datasets containing the family. Both Pearson's r and Spearman's ρ are given and the vast majority is very highly significant due to the very large number of observations in the posterior distribution, except for those marked with † which are non-significant at an α-level of 0.05. We considered for this test only the datasets using the Ethnologue and Harald Hammarstöm's classifications given that they have an unconstrained number of levels.

Category	MBH		MPH		BBH		BPH	
	mean(r)	*sd(r)*	*mean(r)*	*sd(r)*	*mean(r)*	*sd(r)*	*mean(r)*	*sd(r)*
**N=1**								
**Nominal Categories**	**0.54**	0.23	**0.51**	0.26	**0.56**	0.15	**0.63**	0.16
**Verbal Categories**	**0.56**	0.15	**0.58**	0.17	**0.52**	0.16	**0.59**	0.15
**Simple Clauses**	**0.52**	0.21	**0.50**	0.23	**0.45**	0.18	**0.59**	0.18
**Nominal Syntax**	**0.56**	0.16	**0.54**	0.17	**0.42**	0.15	**0.42**	0.17
**Morphology**	**0.54**	0.17	**0.54**	0.16	**0.45**	0.18	**0.41**	0.17
**Phonology**	**0.40**	0.31	**0.42**	0.30	**0.47**	0.18	**0.45**	0.18
**Word Order**	**0.41**	0.32	**0.38**	0.34	**0.37**	0.23	**0.40**	0.24
**N=5**								
**NominalCategories**	**0.58**	0.19	**0.58**	0.19	**0.57**	0.16	**0.62**	0.17
**SimpleClauses**	**0.56**	0.19	**0.56**	0.20	**0.47**	0.18	**0.62**	0.17
**WordOrder**	**0.58**	0.25	**0.55**	0.28	**0.35**	0.25	**0.39**	0.24
**VerbalCategories**	**0.56**	0.15	**0.58**	0.17	**0.52**	0.16	**0.59**	0.15
**NominalSyntax**	**0.61**	0.14	**0.59**	0.14	**0.44**	0.16	**0.42**	0.18
**Morphology**	**0.56**	0.17	**0.57**	0.15	**0.45**	0.18	**0.40**	0.18
**Phonology**	**0.45**	0.30	**0.48**	0.28	**0.46**	0.19	**0.44**	0.19
**N=7**								
**SimpleClauses**	**0.59**	0.17	**0.64**	0.12	**0.47**	0.18	**0.60**	0.18
**NominalCategories**	**0.63**	0.13	**0.59**	0.18	**0.56**	0.16	**0.62**	0.17
**WordOrder**	**0.67**	0.15	**0.65**	0.17	**0.35**	0.23	**0.39**	0.26
**NominalSyntax**	**0.65**	0.11	**0.62**	0.13	**0.44**	0.16	**0.42**	0.19
**VerbalCategories**	**0.57**	0.15	**0.58**	0.17	**0.51**	0.15	**0.59**	0.15
**Phonology**	**0.57**	0.21	**0.58**	0.20	**0.46**	0.19	**0.43**	0.20
**Morphology**	**0.57**	0.16	**0.58**	0.16	**0.45**	0.18	**0.44**	0.16

Table S19: *The correlations between branch length and number of nodes for all types of categories separately for each of the 4 datasets considered. Reported are the mean and standard deviation of Pearson's r across language families and outgroups. We considered for this test only the datasets using Harald Hammarstöm's classification given the computational cost. N=1 considers all families, while N=5 and N=7 only those with data for at least 5 or for all 7 categories.*

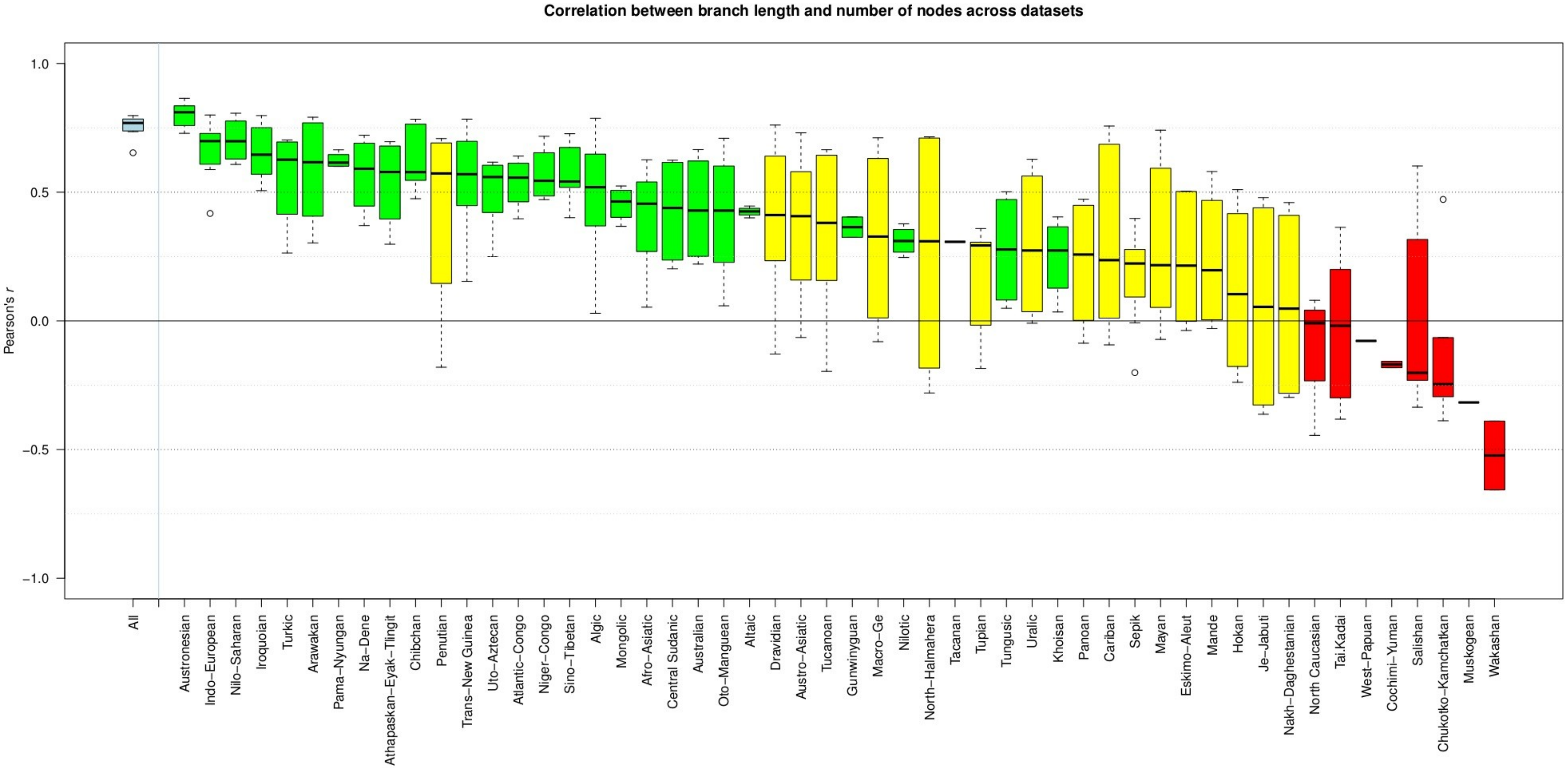

Figure S15*: Correlation between branch length and number of nodes for all families together (leftmost) and for each family individually. The boxplots summarize the Pearson's r across the 8 datasets, red means the median r is below 0, yellow that the minimum r is below 0.*

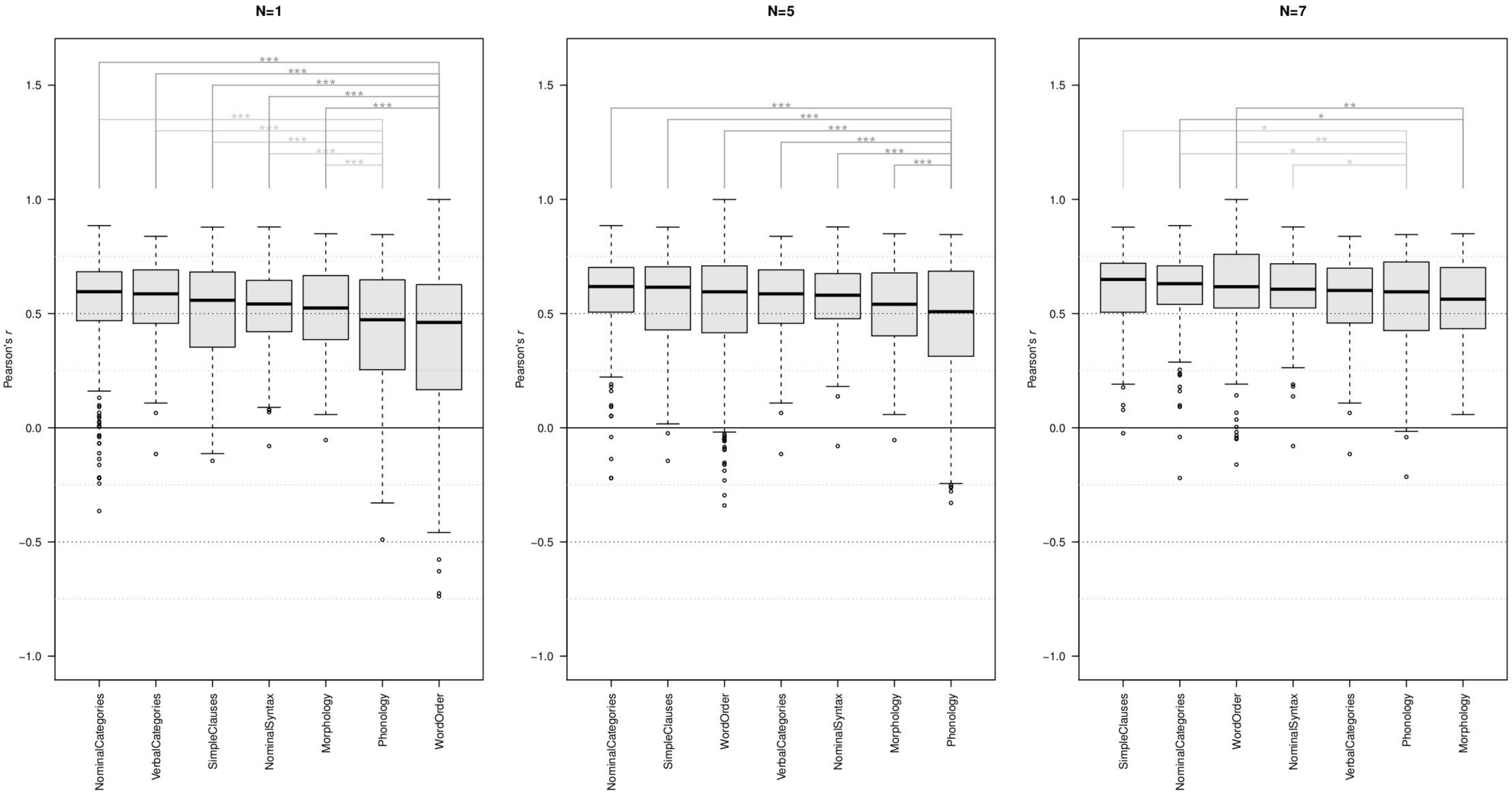

Figure S16: *Boxplots of the correlation (Pearson's r) between branch length and number of nodes for each category across 4 datasets (MBH, MPH, BBH and BPH) and all outgroups. The categories are ordered from highest median correlation (left) to the lowest (right) Shown are the boxplots for all families with data for at least one (N=1), five (N=5) or all (N=7) categories of features. Shown are also the significant pairwise differences (corrected using Tukey's HSD) between categories (different shades of grey are for visual effect only; alpha levels are signified as *=0.05, **=0.01, ***=0.001).*

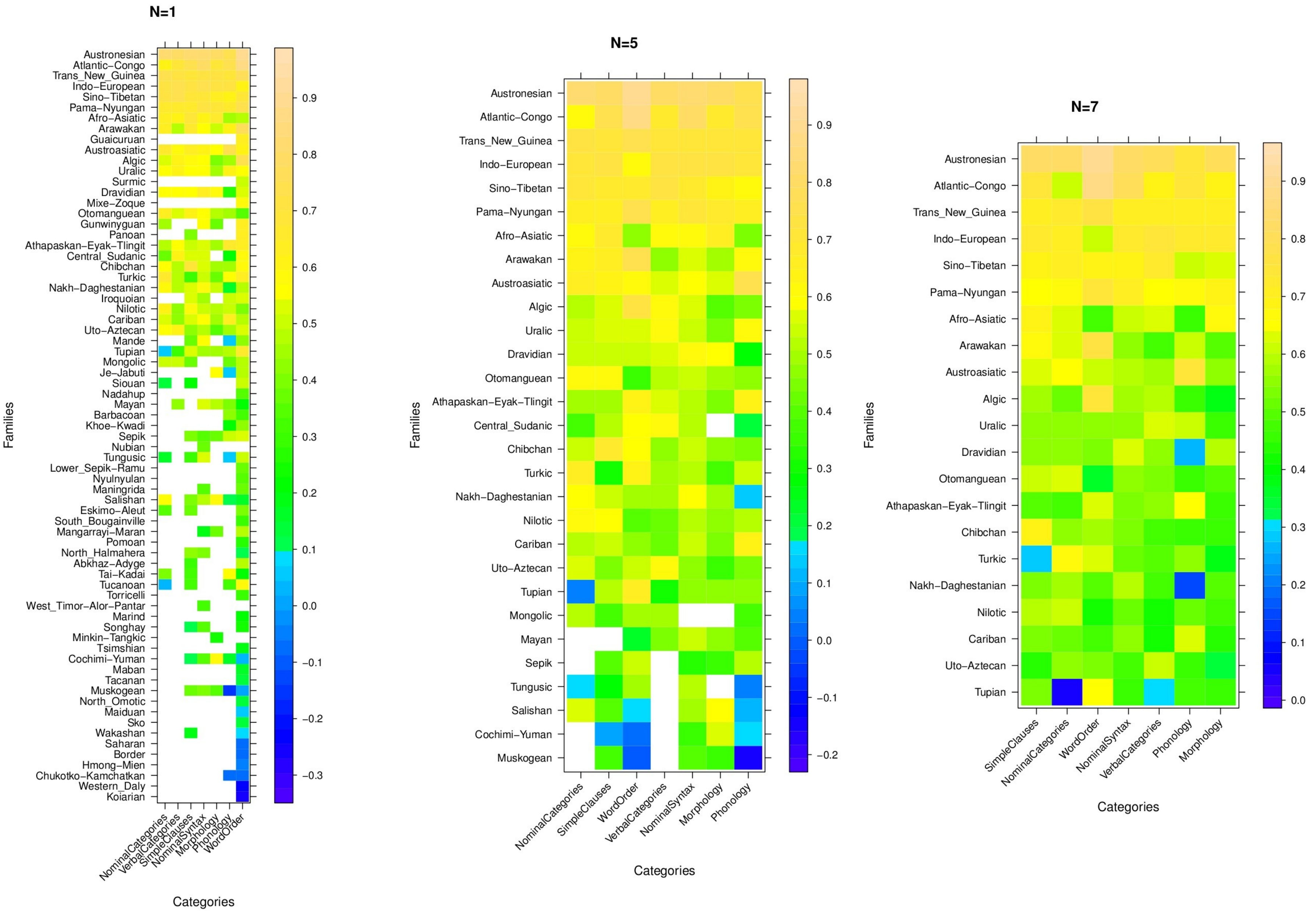

Figure S17*: Intensity plots of the mean correlation (Pearson's r) between branch length and number of nodes for each category and family across 4 datasets (MBH, MPH, BBH and BPH) and all outgroups. The categories are ordered from highest mean correlation (left) to the lowest (right), and the families from top to bottom. Shown are the boxplots for all families with data for at least one (N=1), five (N=5) or all (N=7) categories of features.*

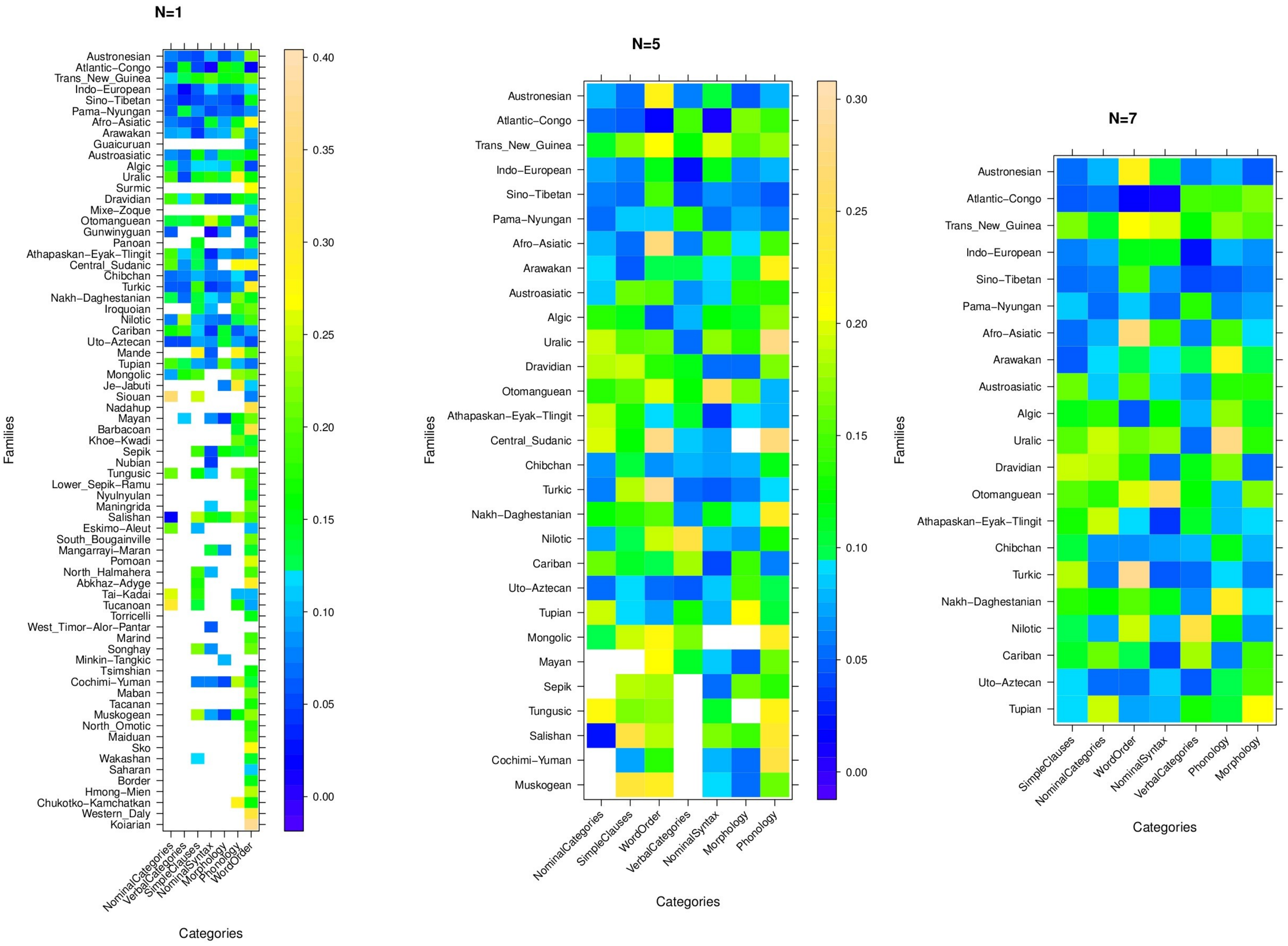

***Figure S18**: Intensity plots of the standard deviation of correlations (Pearson's r) between branch length and number of nodes for each category and family (see Figure S17 for details).*
